# Genetic influences on human blood metabolites in the Japanese population

**DOI:** 10.1016/j.isci.2022.105738

**Published:** 2022-12-06

**Authors:** Takeshi Iwasaki, Yoichiro Kamatani, Kazuhiro Sonomura, Shuji Kawaguchi, Takahisa Kawaguchi, Meiko Takahashi, Koichiro Ohmura, Taka-Aki Sato, Fumihiko Matsuda

**Affiliations:** 1 Center for Genomic Medicine, Kyoto University Graduate School of Medicine, Kyoto 606-8507, Japan; 2 Department of Rheumatology and Clinical Immunology, Kyoto University Graduate School of Medicine, Kyoto 606-8507, Japan; 3 Life Science Research Center, Shimadzu Corporation, Kyoto 604-8511, Japan

**Keywords:** Human Genetics, Human metabolism, Human Physiology

## Abstract

An increase in ethnic diversity in genetic studies has the potential to provide unprecedented insights into how genetic variations influence human phenotypes. In this study, we conducted a quantitative trait locus (QTL) analysis of 121 metabolites measured using gas chromatography-mass spectrometry with plasma samples from 4,888 Japanese individuals. We found 60 metabolite-gene associations, of which 13 have not been previously reported. Meta-analyses with another Japanese and a European study identified six and two additional unreported loci, respectively. Genetic variants influencing metabolite levels were more enriched in protein-coding regions than in the regulatory regions while being associated with the risk of various diseases. Finally, we identified a signature of strong negative selection for uric acid ($\hat{S}$ = −1.53, p = 6.2 × 10^−18^). Our study expanded the knowledge of genetic influences on human blood metabolites, providing valuable insights into their physiological, pathological, and selective properties.

## Highlights

• A Japanese metabolite QTL study identified 60 metabolite-gene associations
• Genetic variants influencing metabolite levels were enriched in the coding region
• Metabolite QTL was associated with disease risk
• A signature of strong negative selection was detected for uric acid

## Graphical abstract

**Figure:**
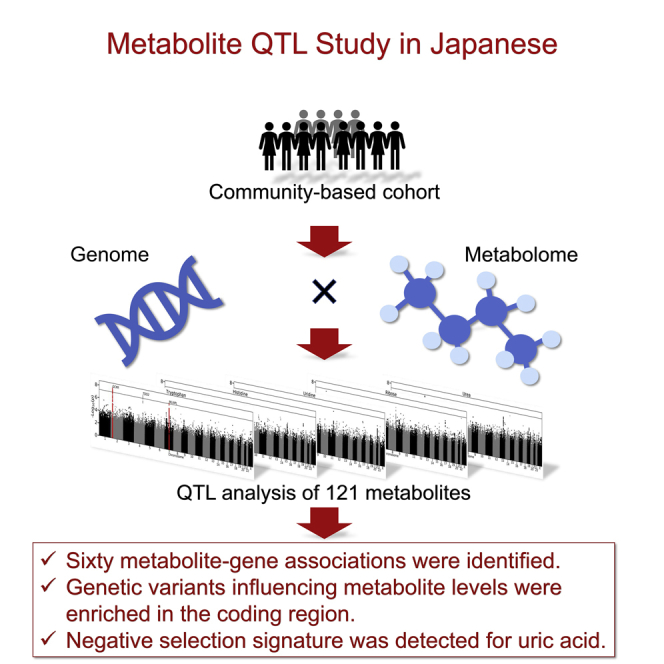

## Introduction

Genome-wide quantitative trait locus (QTL) analysis of metabolites (mQTL) in the European population (EUR) has identified hundreds of common to rare genetic variants associated with human blood metabolites.^1^^,^^2^^,^^3^^,^^4^^,^^5^^,^^6^^,^^7^^,^^8^^,^^9^^,^^10^ These studies have provided heritability estimates of multiple metabolites, insights into the biochemical pathways, and downstream functional implications of disease-associated variants. However, an imbalance in the study population remains a significant limitation of these studies. As in most genome-wide association studies (GWASs), most of such studies target EURs.^11^ This imbalance limits our understanding of the genetic influence on metabolites for two reasons. One reason is that the analysis of complex traits generally strongly depends on genetic architecture.^12^^,^^13^ The other reason is that metabolite levels are affected by environmental factors such as diet, lifestyle, and physical activity, which are often different among ethnicities. The comparison of QTL profiles among multiple ethnicities and the meta-analysis across populations will lead to a better understanding of genetic influences on human blood metabolites. Recent large-scale GWAS of lipids has indicated that a study comprising multiple ancestries could detect candidate genes or variants more efficiently than a study comprising single ancestry.^14^

Furthermore, studies on non-EURs enable us to understand the downstream effects of genetic variants associated with diseases or other traits identified in those populations. Although some studies have focused on non-EURs, such as the Latino,^15^ Middle Eastern,^16^ African American,^17^ and Japanese,^18^^,^^19^ they employed a relatively small number of participants. There is still an unmet need for large-scale studies focusing on non-EURs.

Previous studies have identified thousands of metabolite-disease associations to date^20^; however, their relationship to fitness has not been explored. If the level of a metabolite is beneficial or detrimental to adaptation, the allele of the genetic variants influencing the metabolite level should be under selection pressure. We can infer such natural selection signatures by uncovering the genetic impacts on metabolites.

Overall, analyzing genetic influences on metabolites in the Japanese population has the potential to obtain unprecedented physiological, pathological, and selective insights into them. With these goals in mind, we conducted a GWAS of the blood levels of 121 metabolites quantified by gas chromatography-mass spectrometry (GC-MS) in a Japanese community-based cohort.

## Results

### Identification of 60 pairs of 46 genes and 44 metabolites

We performed a genetic association analysis of 121 metabolites in 4,888 healthy individuals enrolled in the Nagahama Prospective Genome Cohort for the Comprehensive Human Bioscience (the Nagahama study).^21^ We identified 8,905 genetic variants associated with the blood levels of 44 metabolites (p < 4.1 × 10^−10^). The values of the genomic control lambda (λ GC) varied between 0.99 and 1.05 in all metabolites (Table S1). For each metabolite, associated variants located within 500 kb were merged as a single genetic locus (defined as mQTL), and one gene was assigned to each locus (see STAR Methods). In total, 46 genes were assigned, of which ten showed an association with two or more metabolites. *CPS1* was associated with four metabolites, *PPM1K* and *DPEP1* with three, and the remaining seven genes with two metabolites. On the other hand, 13 out of the 44 metabolites were associated with two or more genes. Uric acid, glycine, and 3-hydroxyisovaleric acid showed association with three genes, and the other ten were associated with two genes. Altogether, we identified 60 gene-metabolite pairs (Table 1, Figures S1–S10).

**Table 1:** Result of the GWAS

Putative causal gene	Metabolite	Lead SNP information									Previous reports^d^
		CHR	Position	Reference	ALT	ID	Freq.	Effect	SE	p -value	
*NBPF3*	O-Phosphoethanolamine	1	21817126	G	GGT	rs80212518	0.46	0.20	0.02	4.7E-22	1
	Phosphoric acid	1	21820042	A	G	rs12132412	0.23	0.20	0.03	8.5E-15	2
*THEM4*	3-Hydroxyisovaleric acid	1	151890958	A	C	rs61817697	0.30	−0.16	0.02	3.1E-13	3
*ACP1*	Ribulose	2	217,560	AG	A	rs56350804	0.23	−0.20	0.03	2.3E-14	a
	Arabinose	2	268191	G	T	rs56321614	0.22	−0.24	0.02	2.6E-21	b
*GCKR*	Mannose	2	27730940	T	C	rs1260326	0.42	0.54	0.02	8.3E-181	3,4,5,6,7,8,9
	Threonine	2	27744364	ATT	A	rs373060500	0.43	0.13	0.02	2.8E-11	3,4,6,10,11,12
*SLC1A4*	2-Aminobutyric acid	2	65225088	G	GC	rs72538440	0.73	0.24	0.03	1.1E-20	3,4,5,6
*CPS1*	Acetylglycine	2	211540507	C	A	rs1047891	0.15	0.42	0.03	1.9E-45	3,4,5,6,13
	Glycine	2	211540507	C	A	rs1047891	0.15	1.08	0.02	1.2E-410	3,4,5,6,8,9,11,12,13,14,15,16,17,18,19,20,21,22,23,24,25,26,27,28,29,30
	Serine	2	211540507	C	A	rs1047891	0.15	0.37	0.03	1.5E-38	3,4,5,6,11,12,21,23,24
	Creatinine	2	211595900	C	T	rs72933889	0.16	0.27	0.03	1.7E-22	2,12,31,32,33,34
*GADL1*	beta-Alanine	3	30768667	T	TTTCCCA AATTTG	rs147693330	0.52	−0.45	0.02	7.0E-120	3,6
*SLC2A9*	Uric acid	4	9984541	G	T	rs9994216	0.58	0.17	0.02	5.1E-17	3,5,6,8,9,29,31,35,36,37,38,39,40,41,42,43,44,45,46,47,48,49,50,51,52,53
*SOD3*^c^	Threonic acid	4	24801834	C	G	rs1799895	0.04	−0.87	0.06	1.4E-45	b
*ABCG2*	Uric acid	4	89045331	A	G	rs75544042	0.71	−0.16	0.02	2.5E-13	3,31,35,36,37,39,40,41,42,43,44,46,49,50,54
*PPM1K*	Valine	4	89226422	T	C	rs1440581	0.47	0.13	0.02	1.2E-10	3,5,11,12,16,26,55,56
	2-Oxoisocaproic acid	4	89228383	T	C	rs7660693	0.46	0.13	0.02	3.6E-10	3,5,11
	3-Methyl-2-oxobutyric acid	4	89228383	T	C	rs7660693	0.46	0.15	0.02	1.1E-13	3,5,11
*AGA*	Aspartic acid	4	178413876	T	A	rs12642803	0.45	−0.16	0.02	2.3E-13	3,12
*SDHA*	Succinic acid	5	195139	C	T	rs118046653	0.02	0.68	0.07	1.9E-20	3
*AGXT2*	3-Aminoisobutyric acid	5	35030922	G	A	rs6882350	0.80	1.13	0.02	3.9E-677	1,3,4,6,15,22,57,58,59,60,61,62
*NADK2*	Lysine	5	36266859	CTT	C	rs10556207	0.63	0.13	0.02	3.5E-10	3,11,12
*REV3L*	Tyrosine	6	111545540	T	C	rs354551	0.69	−0.17	0.02	5.6E-15	3,4,5,11,12,16,22,24,26,63,64
*PSPH*	Serine	7	56080426	C	G	rs62457261	0.67	0.32	0.02	1.9E-51	3,4,5,6,11,12,13,17,21,23,24
	Glycine	7	56081213	A	AAAAT	rs36126335	0.67	0.14	0.02	6.7E-11	3,11,12,25,28
*ADHFE1*	2-Deoxytetronic acid	8	67378275	GT	G	rs35896820	0.41	−0.17	0.02	1.9E-15	5
*PYCRL*	Pipecolinic acid	8	144687092	T	C	rs896962	0.35	−0.23	0.02	6.7E-26	b
*CCBL1*	Indolelactic acid	9	131567995	G	A	rs7854319	0.32	−0.15	0.02	2.6E-12	3,4,5,6,24
*DHTKD1*	2-Aminoadipic acid	10	12074109	A	G	–	0.01	0.82	0.13	1.3E-10	3,12
*LMO1*	Threonine	11	8255106	G	A	rs204926	0.25	−0.16	0.03	2.3E-10	3
*SAA1*	2-Hydroxyisovaleric acid	11	18338682	A	C	rs10741740	0.56	−0.22	0.02	1.8E-27	3,4,5,6,8,24,29
*EXT2*	Xylose	11	44085687	C	A	rs7127924	0.21	0.23	0.03	1.7E-19	b
*GLYAT*	Acetylglycine	11	58462882	T	C	rs11229584	0.83	0.22	0.03	5.8E-15	3,13
*SLC22A12*	Uric acid	11	64361219	G	A	rs121907892	0.03	−1.48	0.06	8.0E-130	11,13,31,36,37,40,41,46,50,53
*NOX4*	Cystine	11	89224453	A	C	rs2289125	0.52	0.21	0.02	4.3E-24	3
*PAH*	Phenylalanine	12	103306579	C	T	rs118092776	0.04	1.04	0.05	7.7E-96	3,5,11,12,13,16,21,23,26
*ALDH2*	2-Hydroxyisovaleric acid	12	112230019	G	C	rs4646776	0.27	−0.24	0.02	1.2E-25	b
	2-Oxoisocaproic acid	12	112241766	G	A	rs671	0.27	−0.17	0.02	4.8E-14	11
*HPD*	2-Hydroxyisobutyric acid	12	122289655	G	A	rs372273603	0.82	0.36	0.03	5.3E-41	5,59,60,65
	3-Hydroxyisovaleric acid	12	122303755	C	T	rs2928283	0.23	−0.19	0.02	5.6E-15	3,4,10
*ASPG*	Asparagine	14	104561998	T	C	rs1770983	0.87	−0.34	0.03	2.2E-26	3,4,5,9,11,12,13,14,21,24
*ACSM2A*	3-Indolepropionic acid	16	20466487	T	G	rs117928765	0.17	0.35	0.04	2.3E-21	3,4,5,6,13,24,66
*ACSM2B*	3-Hydroxyisovaleric acid	16	20563528	T	C	rs77863699	0.20	0.37	0.03	1.4E-49	3,4,10,24
*GCSH*	Glycine	16	81147730	C	T	rs4258631	0.79	0.19	0.03	8.2E-15	3,11,12,16,17,21,24,25,26,28
*SLC7A5*	Kynurenine	16	87878076	G	A	rs4843715	0.12	0.22	0.03	7.8E-13	3,4,5,6,12,13,24,57
*DPEP1*	Cystine	16	89693149	C	T	rs79068217	0.48	0.15	0.02	2.9E-12	b
	Hypotaurine	16	89704365	G	C	rs1126464	0.32	0.16	0.02	1.4E-11	b
	Cysteinylglycine	16	89722390	G	A	rs164752	0.40	0.44	0.02	5.6E-101	3,4,10
*SLC13A5*	Citric acid	17	6619817	T	C	rs170148	0.59	0.16	0.02	7.2E-11	5,11,26
*PRPSAP2*	1,5-Anhydro-D-sorbitol	17	18772654	C	CT	rs11460734	0.25	0.17	0.02	3.1E-12	3,6,67
*DCXR*	Ribulose	17	79996451	G	A	rs56001523	0.06	−0.54	0.05	8.4E-32	a
*TYMS*	Arabinonic acid	18	680520	A	C	rs7239738	0.40	−0.31	0.02	6.6E-52	3
*CEP89*	Hypotaurine	19	33423691	T	A	–	0.01	−0.66	0.10	5.3E-11	b
*PRODH2*	4-Hydroxyproline	19	36290977	C	T	rs3761097	0.16	0.29	0.03	6.2E-23	12
	Pipecolinic acid	19	36290977	C	T	rs3761097	0.16	0.45	0.03	2.7E-56	b
*FUT2*	Fucose	19	49206631	A	T	rs1047781	0.41	−0.85	0.02	9.2E-483	65
*TP53RK*	2-Oxoglutaric acid	20	45236778	T	C	rs6124830	0.46	0.17	0.02	3.7E-16	b
*PRODH*	Proline	22	18910844	A	G	rs4269009	0.14	0.68	0.03	2.5E-119	3,4,5,6,8,11,12,13,14,17,21,22,23,24,29,30,68
*MPST*	Creatinine	22	37463858	G	GCAAGGCCCTG TCTGCCATTCTG GTTGCTCCTCGC CCAAGGCCCTG TCTGCCATTCTG GTTGCTCCTCGCC	–	0.42	−0.14	0.02	2.6E-10	b

Frequency and effect size are based on the ALT allele.

a Metabolites for which QTL was found for the first time in this study.

b Newly identified pair of locus and metabolite.

c Metabolites associated with this gene have not been reported.

d References are shown in supplemental information.

For each of the 60 mQTLs, we searched for the putative causal variant which altered the amino acid or expression level of the assigned gene and found it at 43 loci (Table S2). Among the 43 variants, six changed the amino acid and expression levels, 16 changed the amino acid but not the expression levels, and the other 21 altered the expression levels only.

Of the 60 gene-metabolite pairs, 13 pairs consisting of 11 genes and ten metabolites were not reported (Figure S11). Among the 11 genes, *SOD3* was not reported to be associated with metabolites (Figure S11, orange). The remaining ten genes have been reported for their association with other metabolites but not with those identified in this study (Figure S11, green/cyan-colored). Two out of the ten genes, namely *ACP1* and *DCXR*, were associated with ribulose levels for which no QTLs have been shown (Figure S11 in green).

Second, to explore additional variants independently influencing the metabolite levels, we performed a conditional analysis on the variant showing the strongest association (referred to as a lead variant) at each mQTL. We identified 15 such variants for eight metabolites (p < 4.1 × 10^−10^) (Table S3, Figure S12–S21). We assigned a gene to each of those variants and found that five genes had two or more variants associated with five metabolites (Table S3, shown in bold). The sum of the allele dosage of the lead and additional variants that increased the level of a metabolite increased the level of that metabolite (Figure S22). The proportion of variance explained by the lead and additional variants was greater than 10% in four metabolites (3-aminoisobutyric acid, fucose, mannose, and proline) (Table S1).

### Fourteen gene-metabolite pairs were localized in known biochemical pathways

Of the 60 gene-metabolite pairs, 14 were mapped to known biochemical pathways (Table S4). The *DCXR*-ribulose pair, the only pair newly identified in this study, was on the pentose and glucuronate interconversion pathway. DCXR is an enzyme which reduces xylulose to xylitol in the multiple biochemical reactions toward ribulose on that pathway. Rs60208666 is located 497-bp upstream of the transcription initiation site of *DCXR* and is predicted to be in the promoter region.^22^ The G allele of rs60208666, which was reported to increase the expression level of *DCXR*,^23^ decreased ribulose level (beta = −0.51).

### Comparison and the multi-population analysis with the EUR

**Figure 1:**
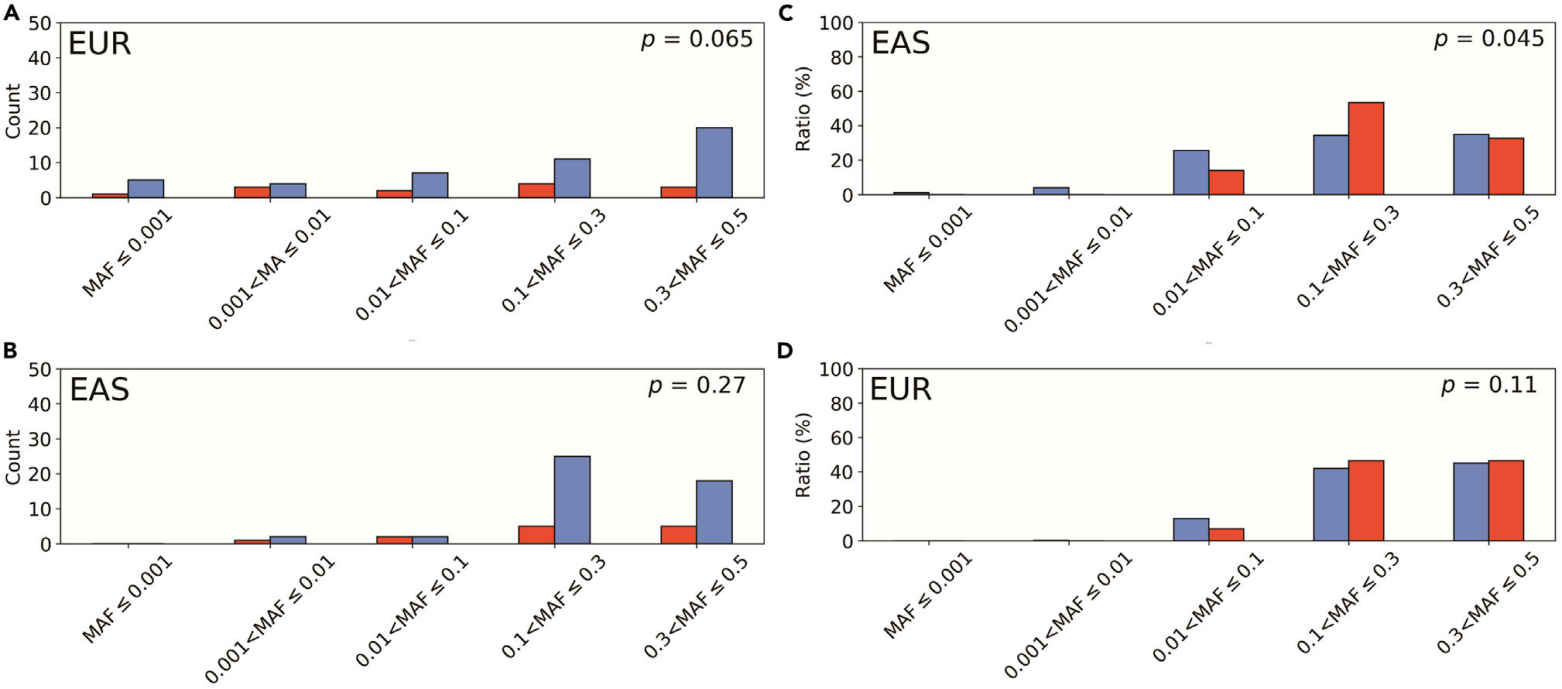
Comparison of minor allele frequencies between the novel and known variants in the European and East Asian populations (A and B) The number of variants falling in the indicated MAF ranges in EUR (A) and EAS (B) is shown with a vertical bar. The red and blue bars indicate novel and known variants, respectively. (C and D) The distribution of variants according to the MAF range in EAS (C) and EUR (D) is shown with vertical bars. The red and blue bars correspond to those with a p value smaller and equal to or larger than 5.0 × 10^−5^, respectively.

To clarify the genetic background of different association signals between the European and Japanese populations, we compared the allele frequencies of the lead variants in 47 known and 13 novel mQTLs. As a result, minor allele frequencies (MAF) of novel variants in the EUR of the 1000 Genomes Project (1 KG) Phase3 were lower than those of known variants (p = 0.065, Mann-Whitney U test; Figure 1A). On the other hand, there was no difference in their MAF in the East Asian population (EAS) (p = 0.27; Figure 1B). Furthermore, we examined whether previously reported variant-metabolite associations in the EUR were observed in the Japanese population. First, we extracted 556 variant-metabolite pairs showing association in Europeans and examined their associations in Japanese (see STAR Methods) (Table S5). One hundred forty pairs showed association (p < 0.05), of which 116 showed directional consistency (82.9%, p for sign test = 1.2 × 10^−15^). When we set the association p value lower than 5.0 × 10^−5^, 43 variants remained, and all showed directional consistency with those of Europeans. MAF of the 43 variants in 1 KG Phase3 were significantly higher than those of the remaining 513 variants in EAS (p = 0.045, Figure 1C) but not in EUR (p = 0.11; Figure 1D).

In addition, we estimated the genetic correlations for 29 of the 77 metabolites measured in both Japanese and European studies.^4^ Forty-eight metabolites were not included in the analysis due to their low heritability (less than 1.0 × 10^−3^) either in Europeans or in Japanese. We found that 13 metabolites showed a significant difference in genetic effects (p < 6.5 × 10^−4^) between the two populations (Table 2). The 13 metabolites included tryptophan (an essential amino acid) and pyruvic acid (the end product of glycolysis), both of which are influenced by dietary habits.

**Table 2:** Result of genetic correlation estimates with the European population

Metabolite	ρ_ge_	SE	*Z*	p value (ρ_ge_ < 1)
**Citrulline**	0.06	0.07	13.99	1.89E-44
**Myo-inositol**	0.23	0.06	12.19	3.32E-34
**1,5-Anhydro-D-sorbitol**	0.14	0.07	11.50	1.29E-30
**Tryptophan**	0.18	0.08	10.85	1.92E-27
**Lauric acid**	0.26	0.08	9.22	3.02E-20
**2-Hydroxyisobutyric acid**	0.26	0.08	9.04	1.63E-19
**Acetylglycine**	0.01	0.12	8.47	2.36E-17
**3-Hydroxyisovaleric acid**	0.31	0.10	7.19	6.66E-13
**Pyruvic acid**	−0.42	0.21	6.76	1.42E-11
**3-Hydroxybutyric acid**	0.04	0.15	6.34	2.24E-10
**Aspartic acid**	−0.47	0.23	6.28	3.46E-10
**Glycine**	0.64	0.08	4.39	1.11E-05
**Valine**	0.44	0.16	3.59	3.31E-04
Uric acid	0.55	0.13	3.40	6.67E-04
Indoxyl sulfate	0.43	0.17	3.28	1.05E-03
Palmitoleic acid	0.50	0.19	2.63	0.01
Tyrosine	0.76	0.20	1.23	0.22
Threonic acid	0.69	0.27	1.17	0.24
Glyceric acid	0.77	0.20	1.14	0.25
Mannose	0.82	0.32	0.57	0.57
Glutamic acid	0.55	0.84	0.53	0.59
Citric acid	0.90	0.20	0.50	0.61
1,6-Anhydroglucose	−0.07	2.38	0.45	0.65
2-Hydroxyisovaleric acid	0.65	2.34	0.15	0.88
Myristic acid	0.26	5.30	0.14	0.89
2-Hydroxybutyric acid	1.00	0.08	0.00	1.00
2-Oxoglutaric acid	1.00	0.10	0.00	1.00
Serine^a^	1.00	–	–	–
Glutamine^a^	1.00	–	–	–

Metabolites are listed in bold font if ρ_ge_ is significantly smaller than 1 (p < 6.5E-04).

a The SE of ρ_ge_ could not be estimated by the jackknife test because all partitions gave the same estimates.

### Meta-analyses discovered additional gene-metabolite pairs

We performed a meta-analysis to identify more mQTLs. First, we used the summary statistics of 55 metabolites in the Tohoku Medical Megabank Organization (ToMMo),^24^ a Japanese community-based cohort (Table S1). We identified 62 gene-metabolite pairs with a significant association (p < 4.1 × 10^−10^, Figures S23–S33, Table S6). Of these, 36 pairs were not detected in our own dataset, and six were not reported. Ten of the 36 pairs were on the known biological pathways. Subsequently, we conducted a multi-population meta-analysis with a European study.^4^ We tested 77 metabolites that were included either in the three datasets (44 metabolites) or in the European and our own studies (33 metabolites) (Table S1). We identified 98 gene-metabolite pairs (p < 4.1 × 10^−10^, Table S7, Figures S34–S50), of which 30 were not found in either our own study or the above Japanese meta-analysis. Among the 30 gene-metabolite associations, two have not been reported previously. Eight out of them were mapped to the known biological pathways.

### Genetic variants influencing metabolite levels were enriched in the coding region

**Figure 2:**
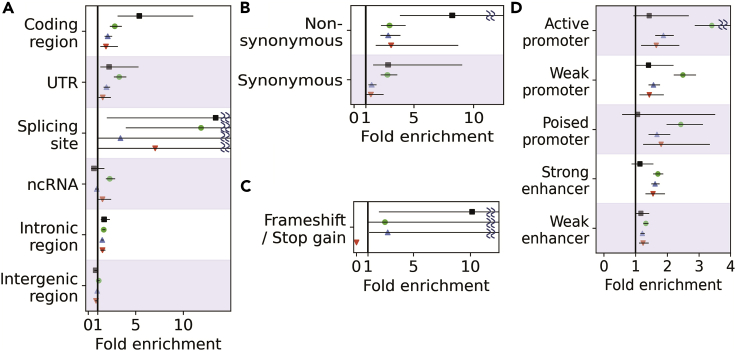
The functional enrichment tests of mQTL, eQTL, clinical measurement QTL, and disease-associated loci (A–D) The results are shown for different portions of the gene (A), amino acid substitution (B), frameshift/stopgain (C) and regulatory elements (D). Black rectangle, green circle, blue triangle, and red inverted triangle correspond to mQTL, eQTL, clinical measurement QTL, and disease-associated loci, respectively. The error bar with a horizontal line indicates a 95% confidence interval. See also Table S8.

We annotated the function of the variants in linkage disequilibrium (LD) (r^2^ > 0.8) with 60 lead (Table 1) and 15 additional variants obtained by the conditional analysis (Table S3) in the 1 KG EAS Phase3. We found that as many as 44 variants (58.7%) had two or more proxy variants in the coding regions. To investigate whether such a high percentage of coding proxy variants exist in the genome, we conducted an enrichment analysis by a random permutation (1,000 times) of chromosome and MAF-matched 75 variants. On average, 8.2 variants (10.9%) had proxy variants in the coding regions, demonstrating a significant enrichment of 5.4-fold (p = 2.0 × 10^−3^) (Figure 2A, Table S8). In addition, we found enrichment in nonsynonymous (8.2-fold, p = 2.0 × 10^−3^) (Figure 2B) and frameshift or stop gain (10.2-fold, p = 0.02) (Figure 2C) variants. On the other hand, there was no significant enrichment in the regulatory region (Figure 2D). We compared the results with clinical measurement QTL,^25^ expression QTL (eQTL),^23^ and disease-associated loci^26^ of the Japanese population. The enrichment of coding region variants was significantly higher (5.4-fold) than that of clinical measurement QTL and disease-associated loci (2.1- and 1.9-fold, respectively) (Figure 2, Table S8). Similarly, the nonsynonymous variants were significantly more enriched (8.2-fold) compared to clinical measurement QTL (2.9-fold). On the contrary, regulatory region variants were more enriched in clinical measurement QTL, eQTL, and disease-associated loci. Such enrichment has been reported for eQTL of non-EAS^27^ and disease-associated loci.^28^

### mQTL was associated with multiple clinical measurements and diseases

To investigate the impact of mQTL on clinical measurements, we performed a phenome-wide association study (PheWAS) using summary statistics of a Japanese GWAS.^25^ We found 138 significant mQTL-clinical measurement associations (Table S9). Associations of variants in the *GCKR* region were the most abundant (43 associations), followed by the *ALDH2* region (40 associations). The *GCKR* region was reported as being associated with dietary habits^29^ in the Japanese population. The metabolites associated with *GCKR* were mannose (a sugar monomer) and threonine (an essential amino acid). 2-hydroxyisovaleric acid and 2-oxoisocaproic acid, which were associated with *ALDH2*, showed significant associations with drinking frequency (p = 2.4 × 10^−20^ and 3.7 × 10^−6^, respectively) (Table S1). Because the *ALDH2* genotypes are known to be associated with drinking habits in Japanese,^29^ differences in alcohol consumption might explain the pleiotropic effects of *ALDH2*.

**Figure 3:**
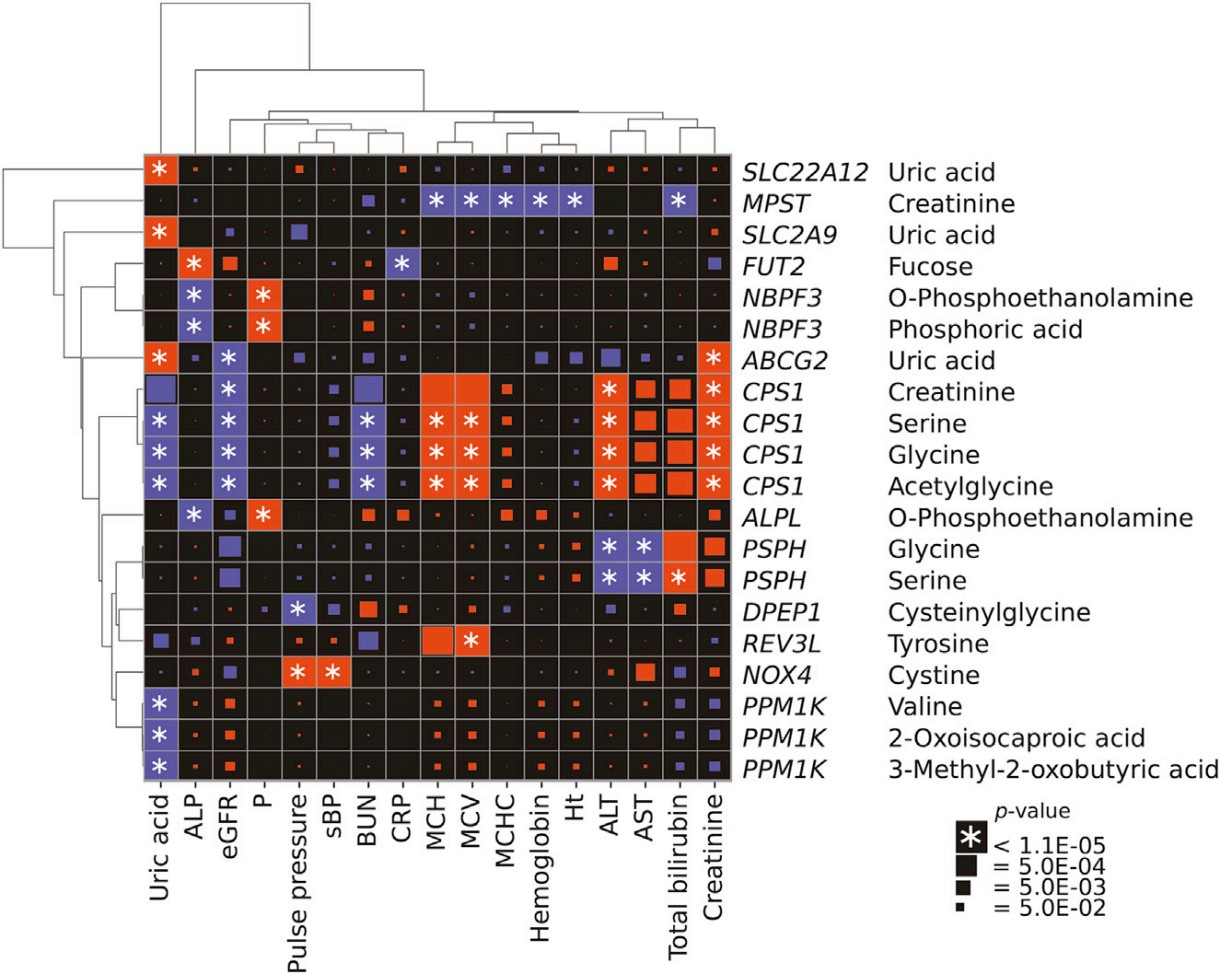
Associations between clinical measurement and mQTL Clinical measurement (horizontal) and mQTL (vertical) combinations with one or more significant associations are shown with the name of the putative causal gene. Red and blue rectangles indicate the allelic direction between the traits being the same and opposite, respectively. The size of rectangles is proportional to the -log_10_(p) value except those showing p < 1.1 × 10^−5^ (with an asterisk). The results of the *GCKR* and the *ALDH2* region are not included. See also Table S9.

Associations of variants in the *CPS1* region were the third most abundant (24 associations, Figure 3, Table S9). The A allele of rs1047891 in *CPS1* increased creatinine, alanine aminotransferase (ALT), mean corpuscular volume (MCV), and mean corpuscular hemoglobin (MCH). In contrast, it decreased blood urea nitrogen (BUN), uric acid, and estimated glomerular filtration rate (eGFR). Furthermore, the T allele of rs72933889 was in LD with rs1047891 (r^2^ = 0.79 in 1 KG EAS) and affected creatinine and ALT levels and eGFR in the same direction as the A allele of rs1047891. However, the association with BUN lost significance (p = 9.3 × 10^−5^).

Subsequently, we conducted an association analysis on 109 of the above 138 association pairs using the phenotype information of the Nagahama Study. We confirmed significant associations of 62 pairs (p < 0.05), and all of them showed a direction consistency (Table S9).

**Figure 4:**
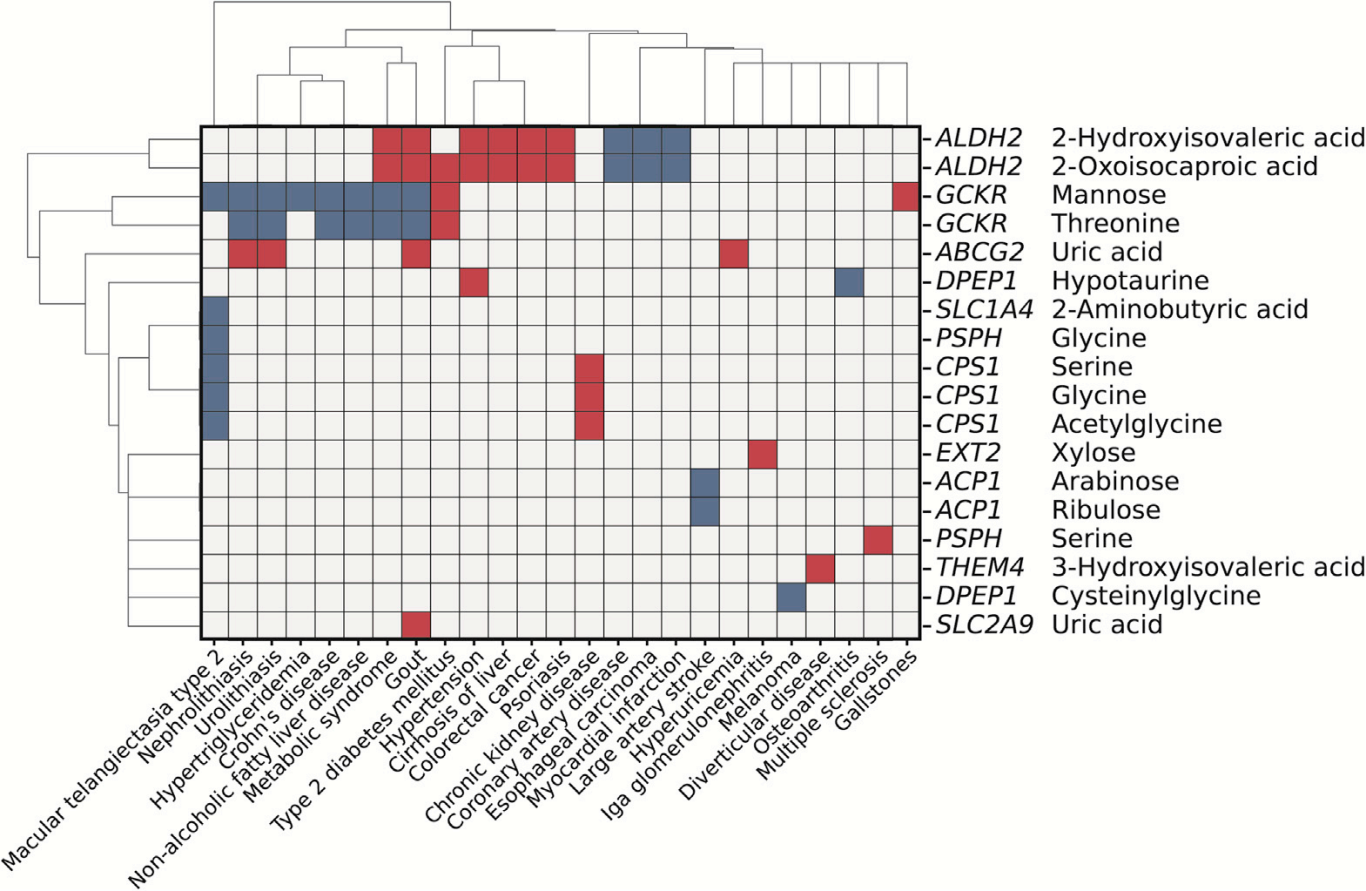
Association between mQTL and diseases Disease (horizontal) and mQTL (vertical) combinations with a significant association are shown with the name of the putative causal gene. Red and blue rectangles indicate the allelic direction between the traits being the same and opposite, respectively.

We next examined the effect of the 75 genetic variants representing mQTL (Tables 1 and S3) on diseases using the GWAS catalog database. We found 57 variant-disease association pairs comprising 18 variants (24%) with 25 disorders (Figure 4, Table S10), which was 6.0-fold (95% confidence interval [CI]: 3.0-Infinity) enriched compared with randomly selected variants (p = 2.0 × 10^−3^). Similar analyses using variants associated with clinical measurement and gene expression revealed that the enrichment was comparable for clinical measurement QTL (13.2-fold, 95% CI: 9.9–19.3) and eQTL (4.2-fold, 95% CI: 3.2–6.0).

Among the 57 mQTL-disease association pairs, the largest pair number was obtained for *ALDH2* (19 pairs), followed by *GCKR* (17 pairs). On the other hand, gout and macular telangiectasia type 2 were associated with the largest number of mQTL (n = 6), followed by metabolic syndrome (n = 4) (Figure 4, Table S10). *ABCG2* and *SLC2A9* were also identified as mQTL of uric acid, the causative agent of gout.^30^ Macular telangiectasia type 2 is a rare neurodegenerative retinal disease characterized by low blood glycine and serine levels.^31^ Indeed, the C allele of rs1047891 in *CPS1* decreased the blood levels of these amino acids and increased disease risk (Figure 4, Table S10).

To further investigate the association between mQTL and disease, we looked up the summary statistics of multi-population GWAS for 1,326 disease phenotypes in the UK BioBank. We found 34 mQTL-disease associations comprising six metabolites and 20 diseases (Table S11, *p*_meta_ < 6.3 × 10^−7^). The 20 diseases were classified into seven endocrine-metabolic, five digestive, and four neoplasms and dermatologic disorders. We examined whether diseases in a specific category were enriched in the 20 diseases by an over-representation analysis. As a result, endocrine and metabolic diseases showed significant enrichment (p = 4.0 × 10^−4^) (Table S12).

Among the 34 mQTL-disease associations, 26 were examined in Europeans and one or more non-EURs, corresponding to 88 European/non-European/disease combinations. We compared the direction of the allelic effect in these combinations and found that 66 (75.0%) showed a direction consistency (sign test; p = 2.9 × 10^−6^).

### Selection signature analysis revealed a strong negative selection signature for uric acid

**Figure 5:**
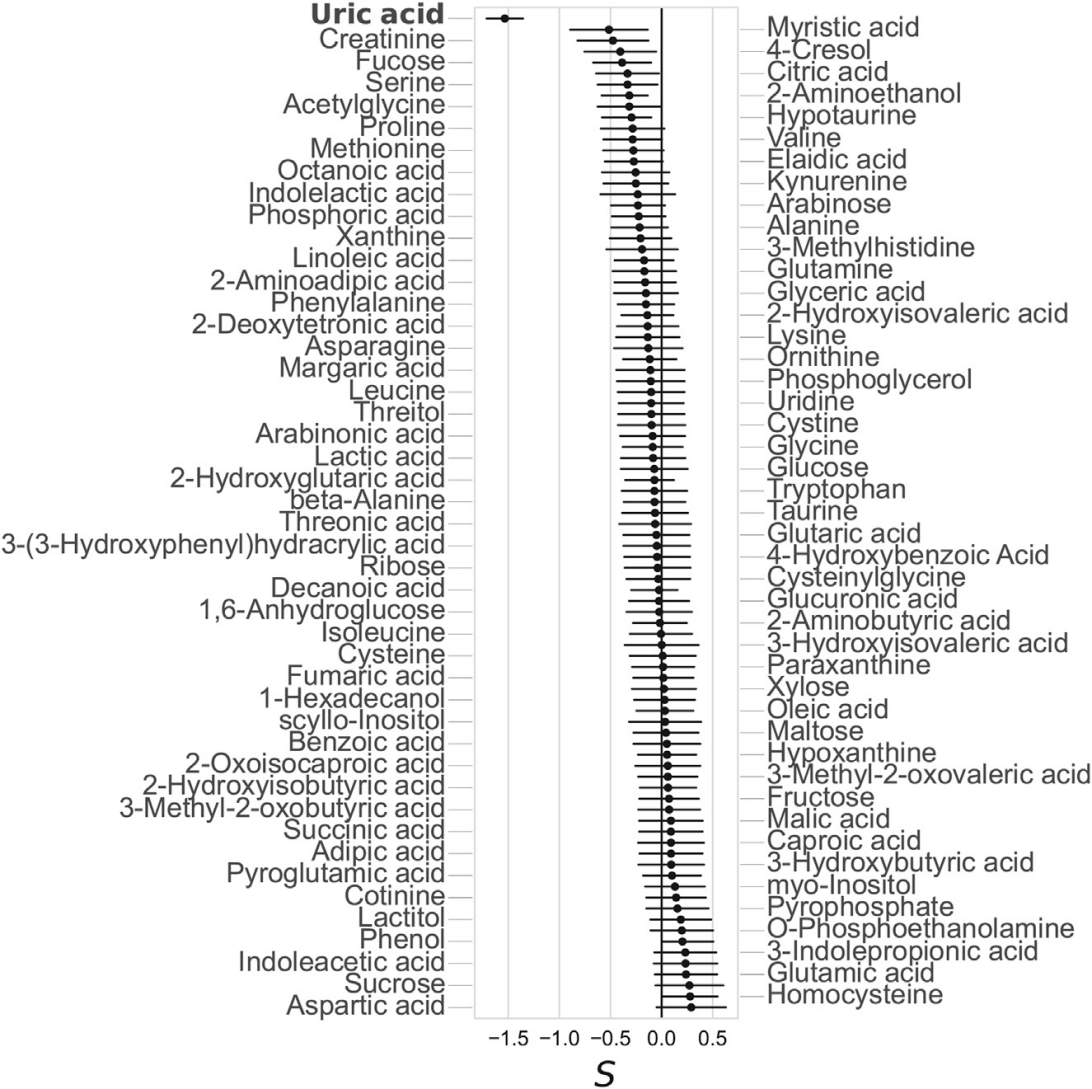
Selection signature analysis of metabolites Ninety-one metabolites that passed the Markov chain Monte Carlo (MCMC) convergence tests are shown. For each metabolite, the mode value of the MCMC chain is shown with a filled circle with an error bar with a horizontal line representing the 95% confidence interval.

We estimated the selection signatures of the genetic effects on metabolites using the BayesS model where *S* < 0 and *S* > 0 indicate the negative and positive selection, respectively.^32^ We obtained a strong negative selection signature in uric acid ($\hat{S}$ = −1.0, p = 1.5 × 10^−11^) and phenylalanine ($\hat{S}$ = −1.0, p = 1.9 × 10^−5^) (Figure S51A). Next, we applied the same method to assess the polygenetic effect on the selection signature after excluding the variants that strongly influenced metabolite levels. More specifically, we removed the lead mQTL variant, those located within 500 kb around it, and the variants in the *CPS1*, *PPM1K*, and *DPEP1* regions that showed pleiotropic effects on three or more metabolites. Only uric acid showed a strong negative selection signature ($\hat{S}$ = −1.5, p = 6.2 × 10^−18^) (Figure 5), suggesting the presence of multiple other variants under negative selection pressure. In contrast, the polygenicity of phenylalanine increased from 3.0 × 10^−3^ to 0.06 (Figures S51B and S51C).

## Discussion

A genome-wide mQTL analysis of 121 human blood metabolites in the Japanese population identified 60 gene-metabolite associations consisting of 46 genes and 44 metabolites. Of note, 13 genetic variants were identified for the first time. Allele frequencies of these variants were lower than those already reported in the EUR (Figure 1A). In contrast, allele frequencies of associated variants in Europeans but not in Japanese were lower in EAS than those showing associations in both populations (Figure 1C). The difference in statistical power due to allele frequencies might partly explain the population-specific genetic associations.

The direction of allelic effects on metabolites was consistent between the European and Japanese populations for most variants showing a strong association (Table S5). However, the allele effects on 13 metabolites estimated by genetic correlation analysis differed between the two populations (Table 2). Differences in environmental factors, such as dietary habits, might explain these.

Of the 13 newly identified gene-metabolite association pairs, only the *DCXR*-ribulose pair was mapped on the single pathway (Table S4). However, we found three other cases where the gene and the metabolite are linked through another metabolite involved in two or more metabolic pathways. In the *PYCRL*-pipecolinic acid association, 5-aminopentaonate is on the arginine and proline metabolic pathway in which *PYCRL* is engaged and on the lysine degradation pathway involving pipecolinic acid (Table S13). It also intermediates the *PRODH2*-pipecolinic acid association (Table S13). Another example is the association between *ACP1* and ribulose which is intermediated by ribulose-5P. The *DPEP1*-cystine association could be physiologically implicated by combining the present findings and the results of previous studies. The T allele of rs79068217 in *DPEP1* decreases *DPEP1* expression (Table S2). On the other hand, the expression levels of *DPEP1* and *SLC3A2*, a cystine transporter, were reported to correlate positively in the human kidney.^33^ These suggest that the T allele of rs79068217 reduces the expression level of *SLC3A2*, resulting in decreased cystine excretion into the urine and, thus, increased cystine levels in the blood.

Metabolite levels had stronger associations with genetic variants influencing the structure and function of gene products than with those regulating expression (Figure 2). This result is in contrast with the findings in the majority of disease-associated variants that are located in noncoding regions.^28^ However, some variants were associated with metabolite level and disease (Figure 4, Tables S10 and S11), highlighting the difference and overlap of mQTL with disease-associated loci.

The A allele of rs1047891 in *CPS1* increased serine, glycine, and acetylglycine and decreased uric acid and BUN in the blood. BUN is the end product of the urea cycle of which *CPS1* functions in the first step. The change of relevant clinical traits (increased ALT, creatinine, MCV, and MCH and decreased eGFR) and metabolite levels could be a consequence of the reduced activity of the urea cycle.

Selection signature analysis indicated that most human blood metabolites were unrelated to fitness. In contrast, many clinical phenotypes have been under selection.^32^ The variation in the level of some metabolites affects diseases but might be tolerant of fitness. Meanwhile, uric acid showed a remarkably negative selection signature. Hypouricemia is associated with several neurological disorders, such as Parkinson disease, Alzheimer’s disease, and multiple sclerosis.^34^ On the other hand, hyperuricemia can also cause various diseases, such as gout or renal disorder.^34^ The multidirectional effect of uric acid on human health might explain its strong negative selection signature. These suggest that uric acid can serve as an essential biomarker of such diseases, affecting the fitness of the Japanese population.

The knowledge of genetic influences on human blood metabolites has been expanded by this study, providing valuable insights into their physiological, pathological, and selective properties. Our results demonstrate the importance of studying genetic effects on the metabolites in diverse ancestries.

### Limitations of the study

This study has the limitation of a relatively small sample size compared to previous European studies. Approximately 75% of variant-metabolite associations detected in Europeans did not show associations in our dataset (p > 0.05, Table S5), which could be attributed to a low statistical power due to the sample size. The second limitation is that we selected 121 metabolites for mQTL analysis, which is only a part of those studied to date. A more comprehensive examination with a larger number of target metabolites would allow more accurate comparisons of mQTL among ethnicities. The third point is that we used only GC-MS to quantify metabolites, and no verification was performed using other technologies. Each technology has its advantages and disadvantages, and measurements by other platforms may reveal new associations not identified in this study.

## STAR★Methods

### Key resources table

**Table:** 

REAGENT or RESOURCE	SOURCE	IDENTIFIER
**Biological samples**		

Healthy individuals enrolled in the Nagahama study	The Nagahama Study	N/A

**Deposited data**		

Summary statistics of GWAS	This paper	https://www.hgvd.genome.med.kyoto-u.ac.jp/repository/HGV0000020.html
Genotype reference panel	1000 Genomes Project (RRID:SCR_006828)	https://www.internationalgenome.org/home
Summary statistics of GWAS for blood levels of metabolites in the Japanese population (ToMMo)	Sakaue et al., 2021^35^	https://jmorp.megabank.tohoku.ac.jp/202102/gwas/TGA000005
Summary statistics of GWAS for blood levels of metabolites in the European population	Shin et al., 2014^4^	http://metabolomics.helmholtz-muenchen.de/gwas/
Summary statistics of GWAS for 58 clinical measurements	Kanai et al., 2018^25^	http://jenger.riken.jp/en/result
Summary statistics of GWAS for 42 diseases	Ishigaki et al., 2020^26^	http://jenger.riken.jp/en/result
Summary statistics of expression QTL analysis on unfractionated peripheral blood	Ishigaki et al., 2017^23^	http://jenger.riken.jp/en/result
Summary statistics of UK Biobank Pan-Ancestry	Pan-UKB team	https://pan.ukbb.broadinstitute.org

**Software and algorithms**		

GCMS solution software	Shimadzu	Version 2.71
SHAPEIT	Delaneau et al., 2011^36^	https://mathgen.stats.ox.ac.uk/genetics_software/shapeit/shapeit.html
Minimac3	Das et al., 2016^37^	https://genome.sph.umich.edu/wiki/Minimac3
PLINK	Purcell et al., 2007^38^	https://www.cog-genomics.org/plink/1.9/, https://www.cog-genomics.org/plink2
METAL	Willer et al., 2010^39^	http://csg.sph.umich.edu/abecasis/metal/download/
ANNOVAR	Wang et al., 2010^40^	http://annovar.openbioinformatics.org/en/latest/
Popcorn	Brown et al., 2016^41^	https://github.com/brielin/Popcorn
GCTB	Zeng et al., 2018^32^	http://cnsgenomics.com/software/gctb/

**Other**		

Mass spectrometer	Shimadzu	GCMS-QP2010 Ultra

### Resource availability

#### Lead contact

Further information and requests for resources and reagents should be directed to and will be fulfilled by the lead contact, Fumihiko Matsuda (fumi@genome.med.kyoto-u.ac.jp).

#### Materials availability

This study did not generate new unique reagents.

### Experimental model and subject details

#### Study population

Plasma and DNA samples were obtained from participants who had taken an extensive health check between 2013 and 2015 in the Nagahama Prospective Genome Cohort for Comprehensive Human Bioscience (the Nagahama Study).^21^ We measured the blood metabolite levels of 8,270 participants and genotyped 7,040 participants. After quality control, 4,888 samples with the age range of 35 and 81 years (mean, 59.0 years) comprising 67.9% females were used for the mQTL analysis. All participants were fully informed of the purpose and procedures of this study, and written consent was obtained from each participant.

#### Metabolite measurement

We collected blood samples (5 mL) of participants who fasted overnight from forearm veins into tubes containing ethylenediaminetetraacetic acid (EDTA; Termo, Tokyo, Japan). We performed sample preparation and GC-MS analysis in the following steps, as described in our previous study.^42^ The internal standard solution (2-isopropylmalic acid, 0.1 mg/mL in purified water) and extraction solvent (methanol: water: chloroform = 2.5:1:1) were mixed at a ratio of 6:250, and added to 50 μL of each plasma sample. The resulting solution was mixed using a shaker at 1,200 rpm for 30 min at 37°C. After centrifugation at 16,000 × g for 5 min at 4°C, 150 μL of the supernatant was collected and mixed with 140 μL of purified water. The solution was thoroughly mixed and centrifuged at 16,000 ×g for 5 min at 4°C. Finally, 180 μL of the supernatant was collected and lyophilized. The lyophilized sample was dissolved in 80 μL of methoxyamine solution (20 mg/mL in pyridine) and agitated at 1,200 rpm for 30 min at 37°C. We added 40 μL of N-methyl-N-trimethylsilyltrifluoroacetamide solution (GL science, Tokyo, Japan) for trimethylsilyl derivatization, followed by agitation at 1,200 rpm for 30 min at 37°C. After centrifugation at 16,000 × g for 5 min at room temperature, 50 μL of the supernatant was transferred to a glass vial. We performed GC-MS analysis using a GCMS-QP2010 Ultra (Shimadzu Corp.). The derivatized metabolites were separated on a DB-5 column (30 m × 0.25 mm id, film thickness 1.0 mm) (Agilent Technologies, Palo Alto, CA). Helium was used as the carrier gas at a flow rate of 39 cm/s. The inlet temperature was 280°C. The column temperature was first held at 80°C for 2 min, then raised at a rate of 15°C/min to 330°C, and held for 6 min. One microliter of the sample was injected into the GC-MS in split mode (split ratio 1:3). The mass conditions were as follows: electron ionization mode with an ionization voltage of 70 eV, ion source temperature of 200°C, interface temperature of 250°C, full scan mode in the range of m/z 85–500, scan rate: 0.3 s/scan. Data acquisition and peak processing were performed using GCMS solution software version 2.71 (Shimadzu, Kyoto, Japan).

We identified low-molecular-weight metabolites as described previously.^42^ Chromatographic peaks were identified by comparing their mass spectral patterns to those in the NIST library or Shimadzu GC/MS Metabolite Database Ver. 1. The identification of metabolites was further confirmed through the coincidence of retention indices in samples with those in the corresponding authentic standards. Retention indices were determined and calibrated daily by measuring the n-alkane mixture (C8-40) (Restek, Tokyo, Japan), which was run at the beginning of the batch analysis. We quantified each metabolite peak using the area under the curve and then normalized using an internal standard.

We checked the linearity of the internal standard (IS; 2-isopropylmalic acid) in the concentration range of 0.03 to 300 μg/mL and confirmed a high correlation (Pearson’s r = 0.9997, Figure S52A). Based on the AUC value at the lowest concentration of IS (0.03 μg/mL), for which linearity was confirmed in the experiment described above, we set the detection limit at AUC = 1,000. We have not evaluated concentration dependence. We demonstrated the high correlation of uric acid and glucose concentrations (Pearson’s r = 0.94, 0.94, respectively, Figures S52B and S52C) measured by clinical laboratory test using 4,888 samples, validating the accuracy of our measurement.

We identified 127 metabolites with known chemical structures in 8,270 samples. Among them, four metabolites were excluded because they were detected in water samples, and two were excluded due to the high relative standard deviation (>1). The median call rate of the remaining 121 metabolites was 99.99 (ranged from 48.0 to 100.0) %. Detailed information for each metabolite, including the biochemical name and class based on its chemical structure, is provided in Table S1. We then conducted a principal component (PC) analysis using the 121 quantified metabolite data of 8,270 samples. We removed four samples as outliers (from −10×IQR (interquartile range) below the 25th percentile to 10×IQR above the 75th percentile in one of the top two inferred axes of variation). To normalize measurement variations caused by inter-day instrument tuning differences, the medians of each run were aligned to 1.0 ^4^^,^^43^, and the proportion of other values was taken. Normalization effects on the machines were visually confirmed (Figure S53). Moreover, PC was significantly associated with measurement dates and instruments before normalization (PC1: p < 1.1 × 10^−16^, PC2: p < 1.1 × 10^−16^, one-way ANOVA) but not after (PC1: p = 1.0, PC2: p = 1.0).

### Method details

#### Genotyping and imputation

Samples were genotyped using Illumina Human610-Quad (n = 1,735), HumanOmni2.5 (n = 1,941), HumanCoreExome-24 (n = 1,721), or Asian Screening Array (n = 1,643). The alleles of all datasets were aligned to GRCh37. After initial quality control of samples and genotypes, we performed the pre-phasing of autosomes and X chromosomes using SHAPEIT ver. 2.837 to treat the male and female samples. Pre-phased autosomes were imputed into the 1000 Genomes Phase3 v5 reference panel^44^ with minimac3 (ver. 1.0.14).^37^ For the X chromosome, we performed the imputation independently for males and females except for the pseudo-autosomal region (from 10,001 bp to 2.6 Mb, from 154.9 Mb to 156 Mb). After imputation, SNPs with MAF smaller than 0.005 or imputation quality R^2^ smaller than 0.3 were removed. Finally, 10,491,983 SNPs included in any of the four datasets were used for the association study. Details of the quality control of samples and genotypes are shown in Figure S54.

### Quantification and statistical analysis

#### Association analysis and meta-analysis

In the association analysis, we excluded outliers (from −3 × IQR below the 25^th^ percentile to 3 × IQR above the 75^th^ percentile) for each metabolite. We calculated residuals for the quantitative metabolite trait by linear regression analysis using age, sex, and top ten PCs as covariates in each group genotyped using four typing kits. Then, a rank-based inverse normal transformation was applied to the estimated residuals. We assumed an additive genetic model and carried out an association test on imputed allelic dosages for these residuals by a linear regression model using PLINK (v2.00)^45^ in each SNP array. For the X chromosome, we conducted GWAS separately for males and females and merged their results by inverse-variance meta-analysis. We combined the association results of four arrays using inverse-variance meta-analysis based on effect size estimates and standard errors, using METAL software (released March 25, 2011).^39^ We set the genome-wide significance threshold at p = 4.1 × 10^−10^; 5.0 × 10^−8^/121 (Bonferroni correction for the 121 metabolites).

#### Locus definition, putative causal gene assignment, and conditional analysis

For each metabolite, we combined variants with significant associations (p < 4.1 × 10^−10^) located within 500 kb and obtained independently associated loci at least 500-kb apart from each other. We refer to such independent associated loci for each metabolite as 'mQTL' (i.e., these could overlap other mQTL). We determined the lead variant with a minimal p value in each mQTL. For each mQTL, we assigned the most likely causal genes by PRoGeM.^46^ For this purpose, two databases for *cis*-eQTL were used. First, we used the significant eQTL (p < 5.0 × 10^−8^) data of peripheral blood in the Japanese population,^23^ and selected proxy variants (r^2^ > 0.8) from the locus of the lead variant in the EAS population of 1KG. We also used the significant cis-QTL prepared in the Genotype-Tissue Expression (GTEx) project (v7) across all tissues assayed (n = 48), and selected proxies (r^2^ > 0.8) of the lead variant from the EUR and the EAS in 1KG. We used the output of “co-occurring” candidates. When there were more than one candidate genes, the nearest gene from the lead variant among the candidate genes was assigned, and when there was no candidate genes, the nearest gene from the lead variant was assigned.

To identify a putative causal variant in each mQTL, we sought variants among proxies that change (i) the amino acid residue (r^2^ > 0.8 1KG EAS) or (ii) the expression level of the mapped gene. ANNOVAR^40^ was used to obtain functional information, and the same eQTL database and r^2^ threshold used for gene assignment were employed to obtain expression information. When multiple variants passed the above criteria, we sorted the variants in the order (i) to (ii) to select one variant. When multiple variants were within the same criteria, we chose the variant with the highest LD with the lead SNP in 1KG EAS.

To assess the biological plausibility between the metabolite and the assigned gene, we used the pathway information recorded in the KEGG database^47^ (http://rest.kegg.jp) released in July 2020. We excluded “Metabolic pathways” (map01100) which included more than 1,000 genes.

A stepwise conditional analysis was performed in the region within 1Mb to the lead variant using the genomic dosage of the top associated variants as a covariate. The stepwise analysis was repeated until no SNPs with significant associations (p < 4.1 × 10^−10^) appeared. The putative causal gene for additional variants was assigned in the same way as described above. The variance of metabolites explained by genome-wide significant SNPs was calculated by determining the coefficient of determination (R^2^) in the multiple linear regression model, in which the dosage of the lead and additional SNPs were set as explanatory variables and the level of the metabolite (age, sex, and top 10PC-adjusted) was selected as the objective variable. The analysis was conducted using the statsmodel package in Python (ver. 2.7.5).

#### Novel locus identification

A detected mQTL was considered novel when its lead variant was located at least 500-kb away from any variants whose association with the corresponding metabolite has been reported. We collected known variant-metabolite associations from the following three resources. The first one is a summary of 40 GWAS for metabolites.^11^ This summary lists SNP-metabolite associations (p < 5.0 × 10^−8^) from 40 metabolite GWAS published from November 2008 to October 2018. We matched the metabolites of our study with this summary by using HMDB identifiers.^20^ The second one is the GWAS catalog,^48^ released September 24, 2021. We first converted chromosomal positions from hg38 to hg19 using the liftOver tool.^49^ Then, we collected metabolite-SNP associations for each metabolite by searching its standard and alternate names registered in the HMDB database.^20^ The third resource is the manually collected papers that were not included in either (i) or (ii).^10^^,^^15^^,^^16^^,^^19^^,^^43^^,^^50^^,^^51^^,^^52^^,^^53^

#### Comparison with European studies

To compare our results with those in the European population, we extracted blood metabolite-variant associations detected in the European population from the following three resources. The first one is the summary of 40 metabolite GWAS ^11^ described above. From this summary, we selected metabolites whose HMDB identifiers ^20^ were given, excluded three studies^3^^,^^9^^,^^54^ which partially or totally lacked information on the direction of allelic effects, selected variants detected in both our study and previous studies, and selected variant-blood metabolite associations. The number of studies and associations in each step are shown in Figure S55. The second resource is the GWAS Catalog (released September 24, 2021)^48^. We chose SNPs that have genome-wide associations (p < 5.0 × 10^−8^) with the trait. Chromosomal positions were converted from hg38 to hg19 using the liftOver tool ^49^. For each metabolite, we searched variant-metabolite associations by searching standard and alternate names registered in the HMDB database^20^. We further excluded studies that do not include the European population. From these associations, we excluded the association included in the first resource, excluded studies that partially or totally lacked information on the direction of allelic effects, selected variants detected in our study and previous studies, and selected variant-blood metabolite associations. The number of studies and associations in each step are shown in Figure S56. The third resource is variant-metabolite associations reported in papers concerning GWAS for human blood metabolites conducted in the European population, published after October 2018 until January 2022, and have not been included in either the first or second resource.^10^ Finally, we combined the results of the three resources. If multiple SNPs existed within 500 kb distance for the same metabolite, the SNP having the lowest p-value among them was selected.

#### Multi-population correlation estimates

We used Popcorn (version 0.9.9)^41^ to estimate the correlation coefficient for the per-allele effect size ρ_ge_ between the current study and the European population^4^ for 77 metabolites measured in both studies. We used the LD scores and summary statistics of variants present in the HapMap3 database^55^ and with MAF above 5% in both 1KG EUR and 1KG EAS. Here, we excluded 46 metabolites whose estimated heritability was less than 1.0 × 10^−3^ in either one of the populations to calculate accurate multi-population genetic correlation.^56^ The significance threshold was set to p = 6.5 × 10^−4^ for the jackknife test of ρ_ge <_ 1 to correct for 77 multiple tests.

#### Meta-analysis with ToMMo and multi-population analysis

We performed a meta-analysis with another Japanese cohort (ToMMo) and a multi-population analysis with a fixed-effect model based on Stouffer’s Z-score method. METAL software^39^ was used for sample size weighting. We downloaded publicly available GWAS summaries of the Japanese^35^ and Europeans.^4^ For the European results, we converted the genome coordinates of hg18 to hg19 using the UCSC liftOver tool.^49^ For the meta-analysis with ToMMo, we analyzed the variants detected in both studies. As for multi-population analysis, we examined the variants identified in the Japanese (either the current study or ToMMo) and the European study. The significance threshold was set to p = 4.1 × 10^−10^. The same definition was used for the locus and assignment of the gene. The detected locus was considered “additional” when the lead variant was located at least 500-kb away from any variants showing genome-wide significance in the association with the corresponding metabolite.

#### Functional annotation, enrichment analysis, and heritability estimates

To obtain functional annotation of variants, we used ANNOVAR (April 16, 2018, released^40^) for the portion of the gene, amino acid substitution and frameshift/stopgain. For promoter and enhancer region annotation, we used a chromatin state database of nine cell types.^22^ In each type of function, we conducted enrichment analysis for mQTL using the following steps. 1) Let *N*_*v*_ be the count of the number of the lead (Table 1) and additional variants (Table S3) (in total, n = 75) in LD (r^2^ > 0.8) with a variant annotated in the selected function. The 1KG EAS population was used to calculate LDs. 2) We randomly selected 75 variants from the chromosome and MAF-matched (MAF difference <0.025) variants in 1 KG EAS data and counted the number of variants in the same manner as in step 1. 3) Step 2 was repeated 1,000 times, the counted number labeled as *N*_*1*_ … *N*_*1000*_. 4) Fold enrichment of the selected function type was estimated by a mean of *N*_*v*_*/N*_*1*_ to *N*_*v*_/*N*_*1000*_, and the empirical p value was calculated by sorting counts *Nv* and *N*_*1*_ to *N*_*1000*_.

We compared the results with 649 lead variants (at least 500 kb apart, p < 2.1 × 10^−12^; 5.0 × 10^−8^/23,018) of eQTL^23^ and 174 lead variants (at least 500 kb apart, p < 1.2 × 10^−9^; 5.0 × 10^−8^/42) of disease-associated loci in the Japanese population,^26^ and 960 lead variants (at least 500-kb apart, p < 8.6 × 10^−10^; 5.0 × 10^−8^/58) of clinical measurement-associated loci Japanese.^25^

#### Phenome-wide association study

We examined the associations of 60 lead (Table 1) and 15 additional (Table S3) variants with 58 clinical measurements, using summary statistics of a Japanese GWAS.^25^ When the variant was absent from the dataset, we selected the one from 1 KG EAS which showed the strongest LD with the metabolite-associated variant. The significance threshold was set to p = 1.1 × 10^−5^ to correct for 4,350 (75 × 58) multiple tests. To visualize the results, Z values were clustered by Euclidean distance.

The association between the number of drinking days per week and metabolite levels was tested for the same 4,888 samples by an ordinary linear regression model using the statsmodel package in Python (ver. 2.7.5). As for drinking habit data, we used the results of the questionnaire, “How many days do you drink per week?”. Age, sex, and top ten PC-adjusted metabolite quantification values were used.

The results were validated using the data from 4,888 samples of the Nagahama Study. Association analyses were performed using the same methods as those for the metabolites.

#### Association of mQTL with disease

We used the NHGRI GWAS catalog (released September 24, 2021)^48^ and converted chromosomal positions from hg38 to hg19 using the UCSC liftOver tool.^49^ First, we extracted variant-trait associations that passed all of the following criteria: i) the variant is present in our own dataset, ii) with allelic direction information, and iii) the trait was categorized as a disease based on the definition of experimental factor ontology.^57^ Next, we assigned the lead and the additional variants to the variant-trait association using the following two criteria: i) the variant is in high LD (r^2^ > 0.8) with the trait-associated variant in 1 KG EAS, and the study participants of that trait included East Asians, ii) the variant is in high LD (r^2^ > 0.8) with trait-associated variants in 1KG EAS and 1KG EUR, and the study participants of that trait included Europeans, but not East Asians. When multiple variants passed the above criteria, we chose the proxy variant in 1KG EAS.

The enrichment analyses of QTLs for disease-associated loci were conducted using the same procedure as the functional enrichment analysis. We matched the variant distance to the nearest gene (+/− 50% of the variant with the closest gene) as well as the chromosome and MAF.

We also examined the associations of those 75 variants with disease phenotypes using summary statistics of multi-population GWAS (https://pan.ukbb.broadinstitute.org). We used 1,326 phecode phenotypes. When the corresponding variant was absent in the GWAS, we chose the variants with high LD (r^2^ > 0.8) in EAS and other populations used in the study, prioritizing the variant with the highest LD (r^2^) in EAS when multiple variants satisfied that criterion. When no variants met that criterion, we did not test the association. LD was calculated in the nearest 1 KG population: African (AFR), EAS, EUR and Admixed American (AMR) were matched as is, while Central/South Asian (CSA) was matched to South Asian (SAS), and Middle Eastern (MID) was matched to EUR. The significance threshold was set to p = 6.3 × 10^−7^, correcting for 99,450 (1,326 × 75) multiple tests.

#### Estimation of negative selection signature of metabolite

We used the Bayesian mixed linear model (MLM) method with GCTB (ver. 2.0)^32^ for the estimation of a selection signature *S* (*S* < 0 indicates negative selection while *S* > 0 shows positive selection). For genotype preparation, we selected imputed genotypes with r^2^ over 0.9 in each typing kit and converted the allele dosage into the best-guess genotype ([0,0.1] to 0, [0.9,1.1] to 1, [1.9, 2.0] to 2). We further selected 1,191,918 SNPs with call rates of over 97%, MAF over 0.01, and Hardy-Weinberg p-value over 1.0 × 10^−6^ in all datasets with four different typing arrays. We estimated the selection signature for each metabolite by two sets of variants. The first set included all the 1,191,918 SNPs. The second set was generated from the first set by removing the QTLs associated with corresponding metabolite (p < 1.4 × 10^−10^ and within 500-kb distance to the lead variant) and pleiotropic QTLs associated with at least three metabolites which corresponded to *CPS1* (chr2 211-212 Mb), *PPM1K* (chr4 88.7-89.7Mb), and *DPEP1* (chr16 89.7-90.7Mb).

Considering the relatively small sample size (n = 4,888), we set the prior for *S* to 0.1 ^32^. We used the nested BayesS model, in which we partitioned the genome into 200-kb non-overlapping segments and skipped over the windows with zero effect. The chain length was 25,000 iterations, with the first 5,000 discarded as burn-in. In other points, the default parameters implemented in GCTB were used. The convergence of the Markov chain Monte Carlo (MCMC) chain was tested using the Geweke test and Heidelberger and Welch’s convergence diagnostic test.^32^ The significance threshold for each test was set to Z = 2 for the Geweke test and p = 0.05 for Heidelberger and Welch’s convergence diagnostic test. The CODA package in R was used to perform the tests. We used the posterior mode and standard deviation of the MCMC samples to estimate the parameters and their standard errors, respectively. Finally, metabolites with estimated parameters *S*, heritability, and polygenicity that passed all convergence tests were reported. We tested for *S* ≠ 0 using a two-sided Wald test. The significance threshold was set to p = 4.1 × 10^−4^ to correct for 121 multiple tests.

## Data Availability

• Summary statistics of GWAS were deposited in the Human Genetic Variation Database (https://www.hgvd.genome.med.kyoto-u.ac.jp/repository/HGV0000020.html). These data can be downloaded without restriction. • We used publicly available software for the analyses. • Any additional information required to reanalyze the data reported in this paper is available from the lead contact upon request.

## Supplemental information

Document S1. Figures S1–S54

Table S1. Details of the metabolites which were used in the genome-wide association study, related to STAR Methods

Table S2. Putative causal variant assigned to each metabolite-associated locus, related to Table 1

Table S3. Result of the conditional analysis, related to Table 1

Table S4. List of gene-metabolite associations in which the assigned genes are included in the biological pathway with which the paired metabolite was associated, related to Table 1

Table S5. Comparison of the results of the current study with previous studies in the European populations, related to Figure 1

Table S6. Result of the meta-analysis with ToMMo, related to Table 1

Table S7. Result of the multi-population meta-analysis with the European population, related to Table 1

Table S8. Result of functional enrichment analysis, related to Figure 2

Table S9. Result of the PheWAS, related to Figure 3

Table S10. Association of mQTL with disease, related to Figure 4

Table S11. Effect of metabolite-associated variants on disease phenotypes in UKBB, related to Figure 4

Table S12. Over-representation analysis of category of diseases which were associated mQTLs, related to Figure 4

Table S13. List of gene-metabolite associations which can be linked by using two or more pathways, related to Table 1
