# Supplementary material for: Genetic influences on human blood metabolites in the Japanese population
Source: iScience. 2022 Dec 6;26(1):105738. doi: 10.1016/j.isci.2022.105738 (PMC9792902; doi:10.1016/j.isci.2022.105738)
Supplement: Document S1. Figures S1–S54 [file mmc1.pdf]

iScience, Volume *26*

Supplemental information

Genetic influences on human blood metabolites in the Japanese population

Takeshi Iwasaki, Yoichiro Kamatani, Kazuhiro Sonomura, Shuji Kawaguchi, Takahisa Kawaguchi, Meiko Takahashi, Koichiro Ohmura, Taka-Aki Sato, and Fumihiko Matsuda

Figure S1. Regional association plots of identified loci in the current study, Related to Table 1

The calculated r^2 with the lead SNP in the 1KG Phase 3 East Asian (EAS) population are shown in color. The gray plots indicate the lead SNP or the corresponding SNP was a monomorphic variant.

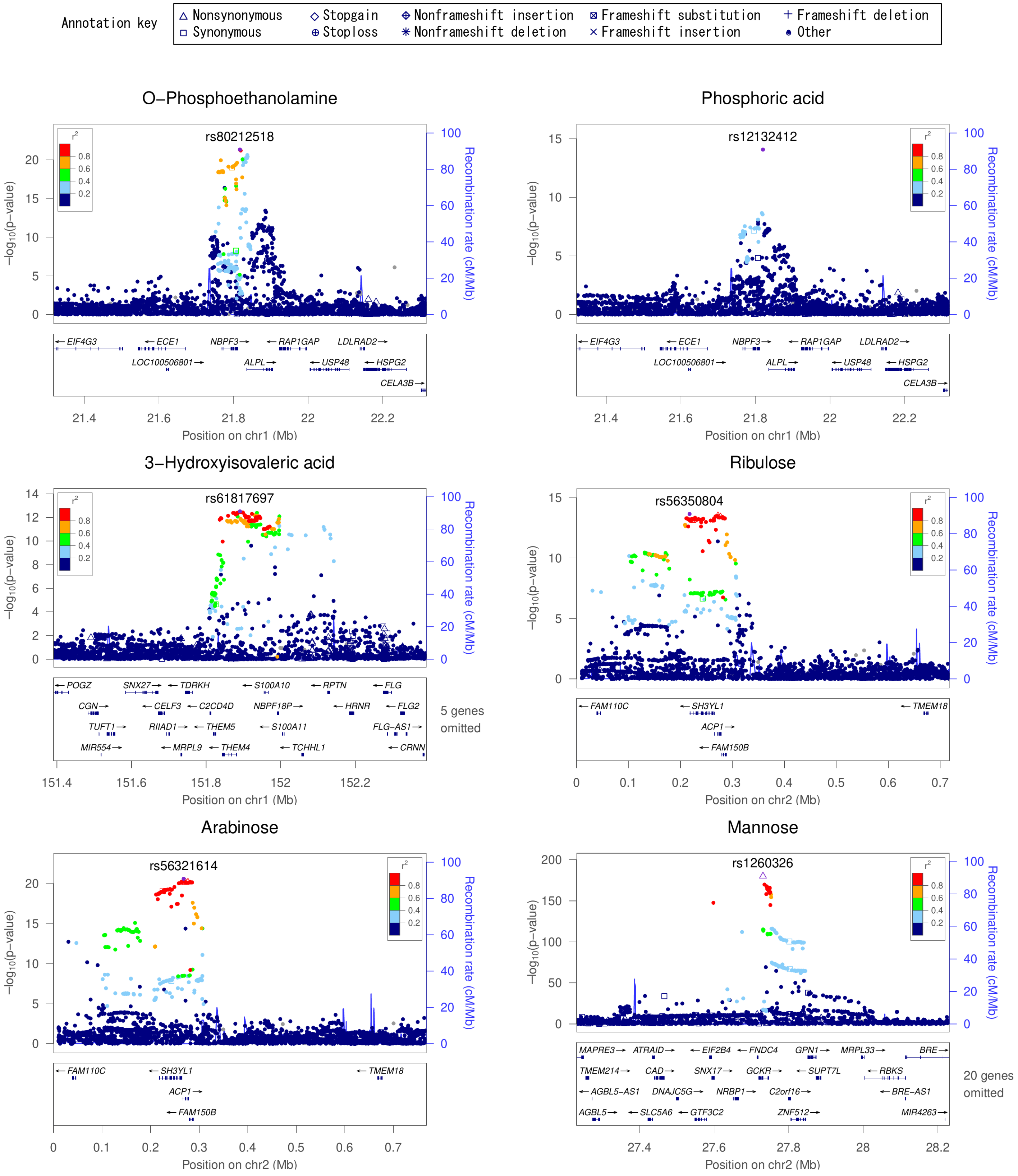

Figure S2. Regional association plots of identified loci in the current study, Related to Table 1

The calculated r^2 with the lead SNP in the 1KG Phase 3 East Asian (EAS) population are shown in color. The gray plots indicate the lead SNP or the corresponding SNP was a monomorphic variant.

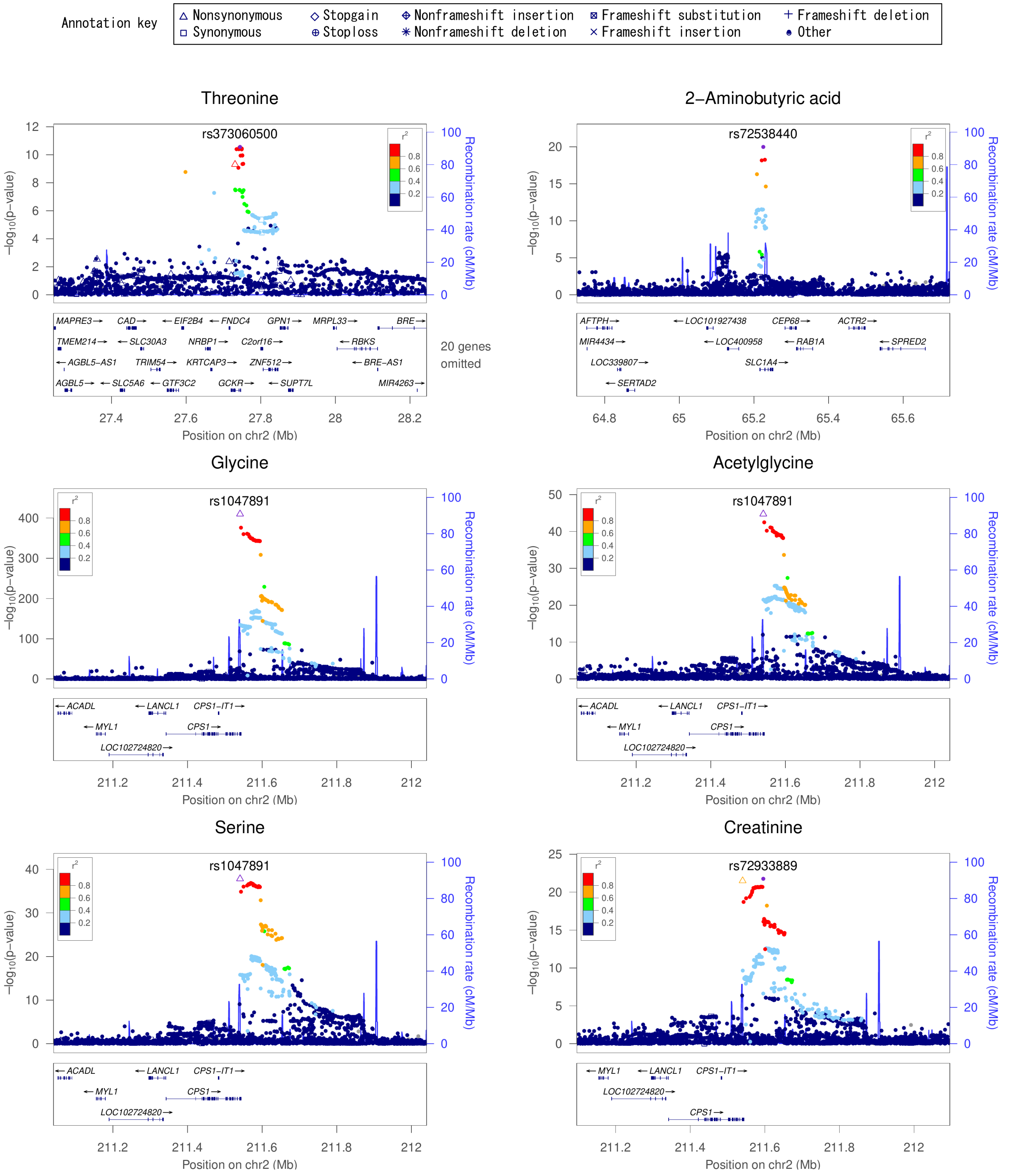

Figure S3. Regional association plots of identified loci in the current study, Related to Table 1

The calculated r^2 with the lead SNP in the 1KG Phase 3 East Asian (EAS) population are shown in color. The gray plots indicate the lead SNP or the corresponding SNP was a monomorphic variant.

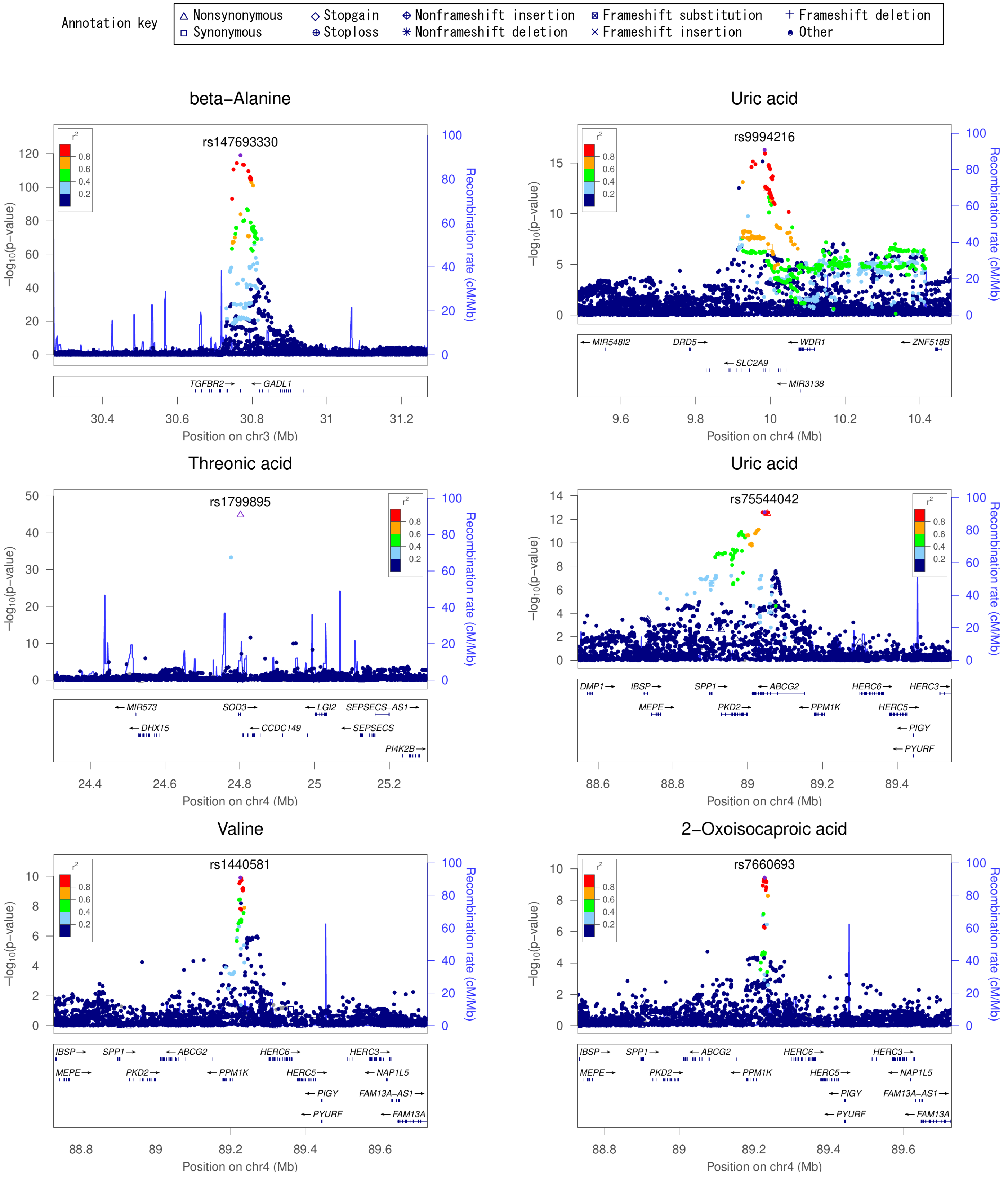

Figure S4. Regional association plots of identified loci in the current study, Related to Table 1

The calculated r^2 with the lead SNP in the 1KG Phase 3 East Asian (EAS) population are shown in color. The gray plots indicate the lead SNP or the corresponding SNP was a monomorphic variant.

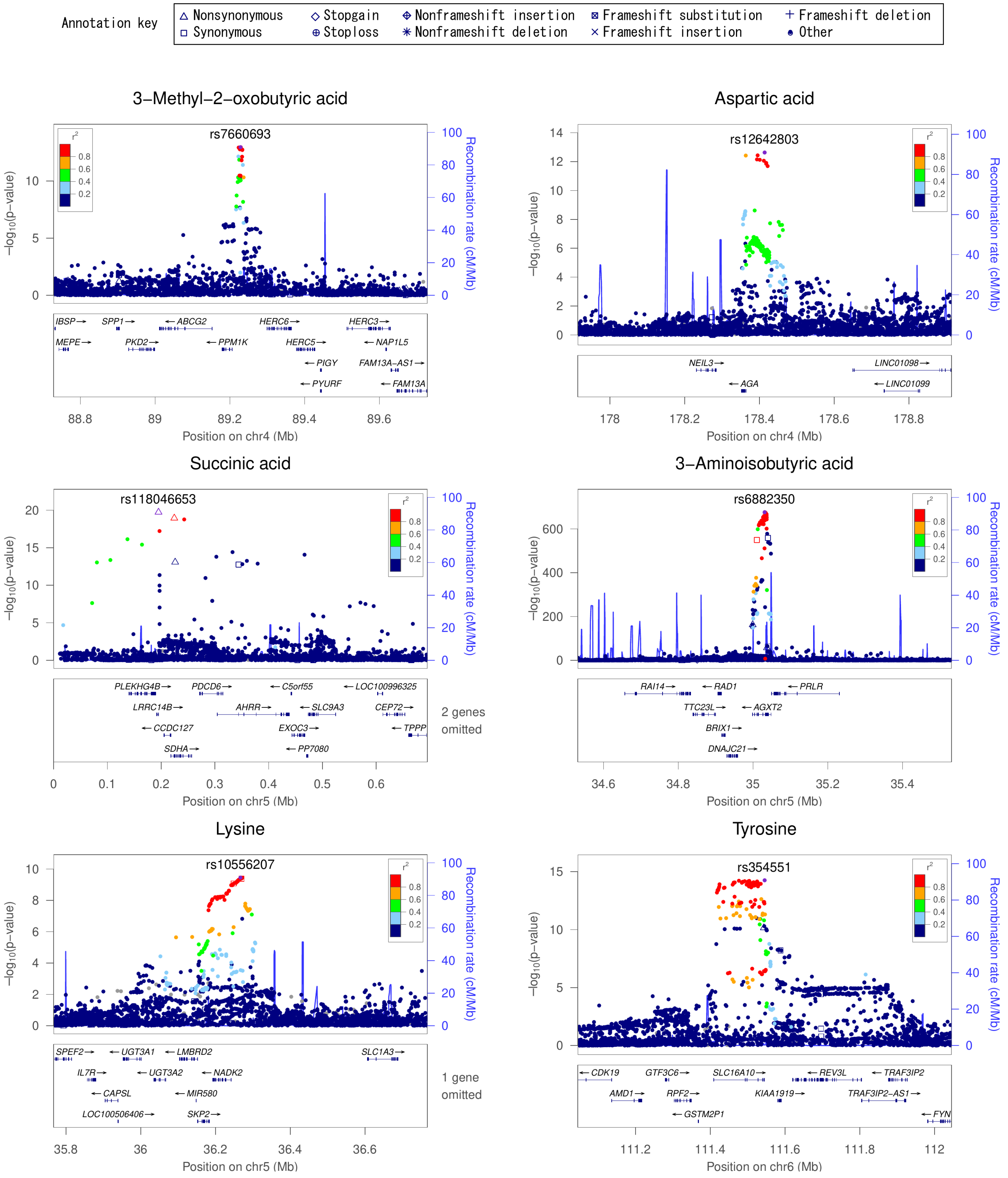

Figure S5. Regional association plots of identified loci in the current study, Related to Table 1

The calculated r^2 with the lead SNP in the 1KG Phase 3 East Asian (EAS) population are shown in color. The gray plots indicate the lead SNP or the corresponding SNP was a monomorphic variant.

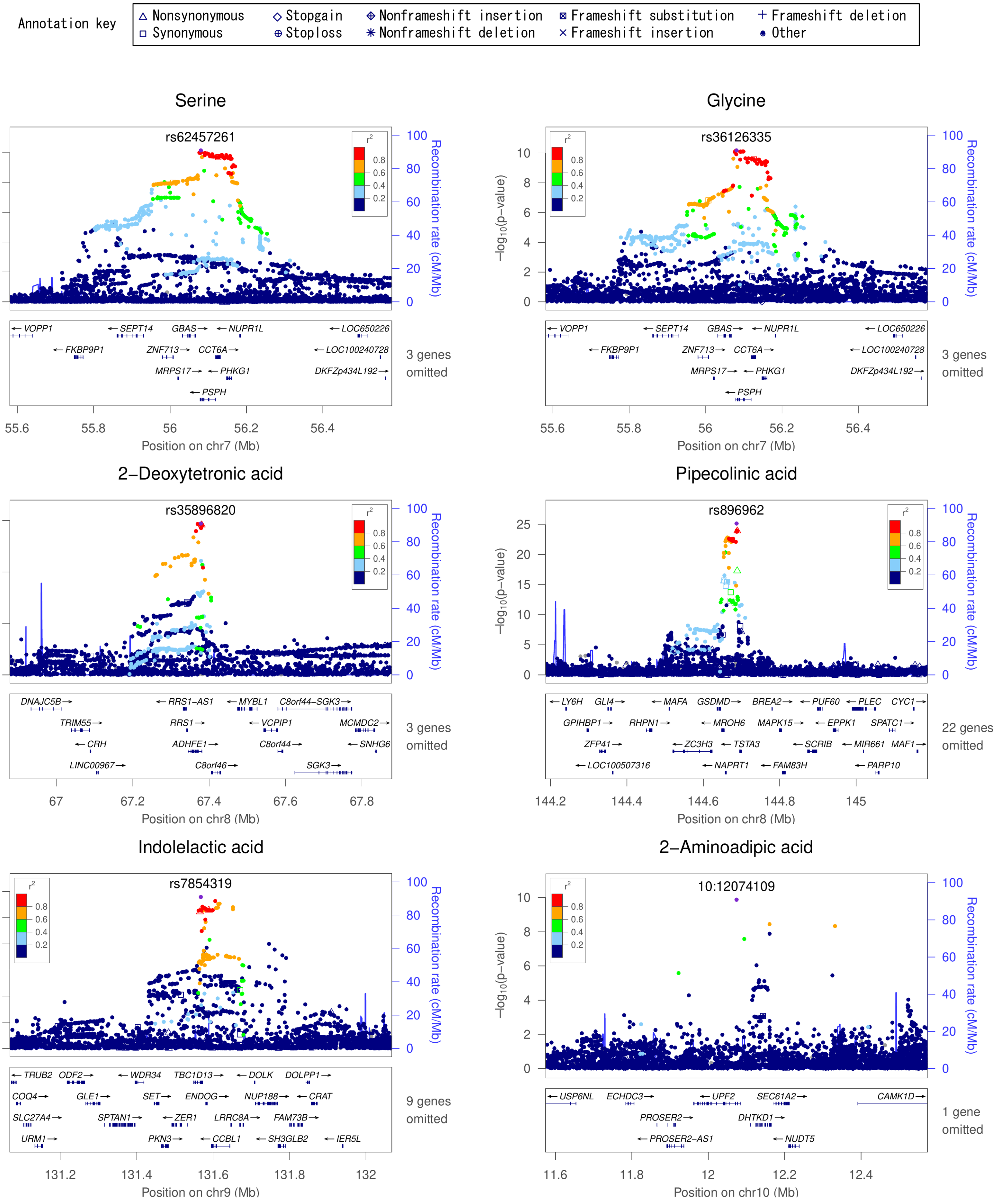

Figure S6. Regional association plots of identified loci in the current study, Related to Table 1

The calculated r^2 with the lead SNP in the 1KG Phase 3 East Asian (EAS) population are shown in color. The gray plots indicate the lead SNP or the corresponding SNP was a monomorphic variant.

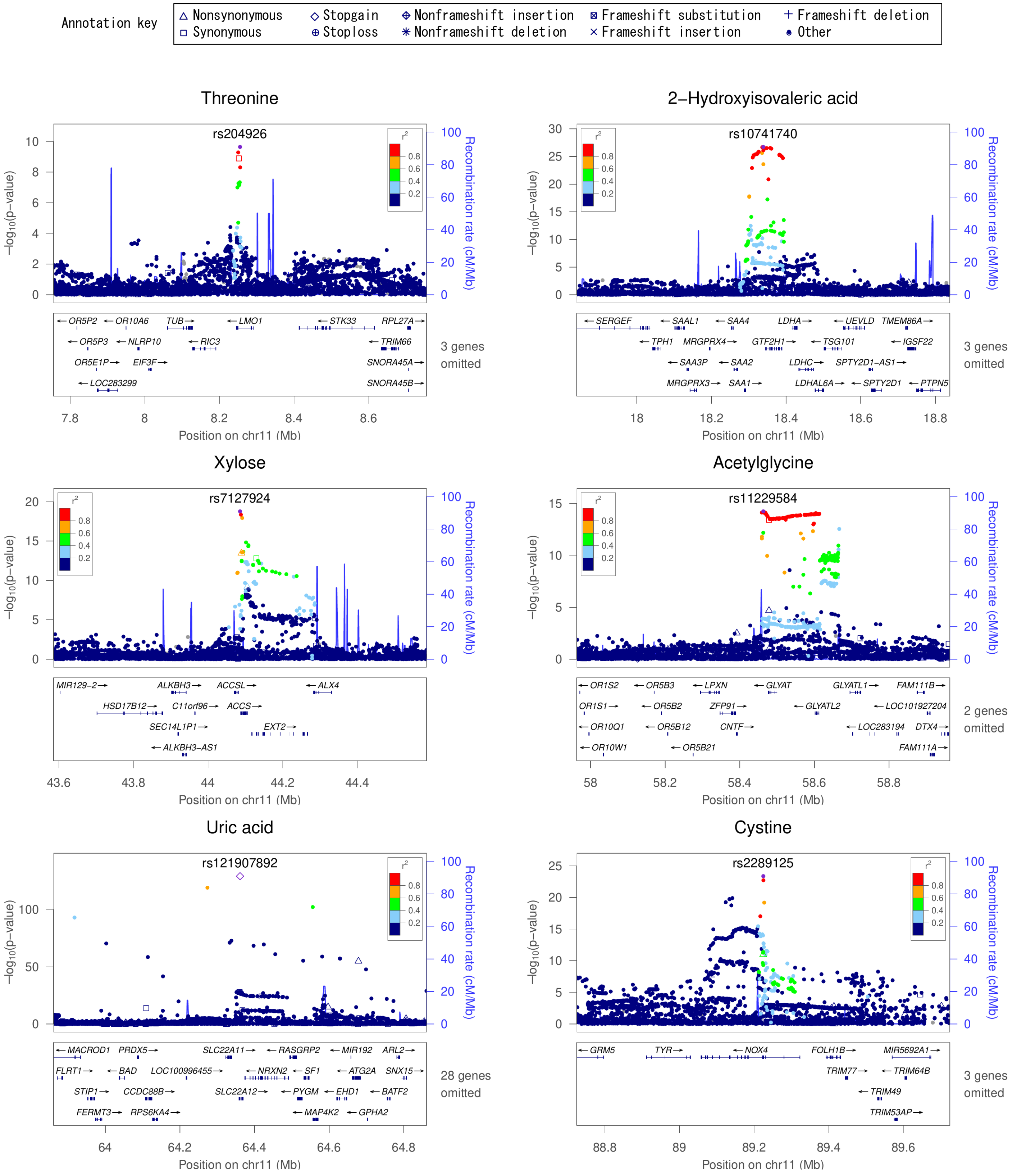

Figure S7. Regional association plots of identified loci in the current study, Related to Table 1

The calculated r^2 with the lead SNP in the 1KG Phase 3 East Asian (EAS) population are shown in color. The gray plots indicate the lead SNP or the corresponding SNP was a monomorphic variant.

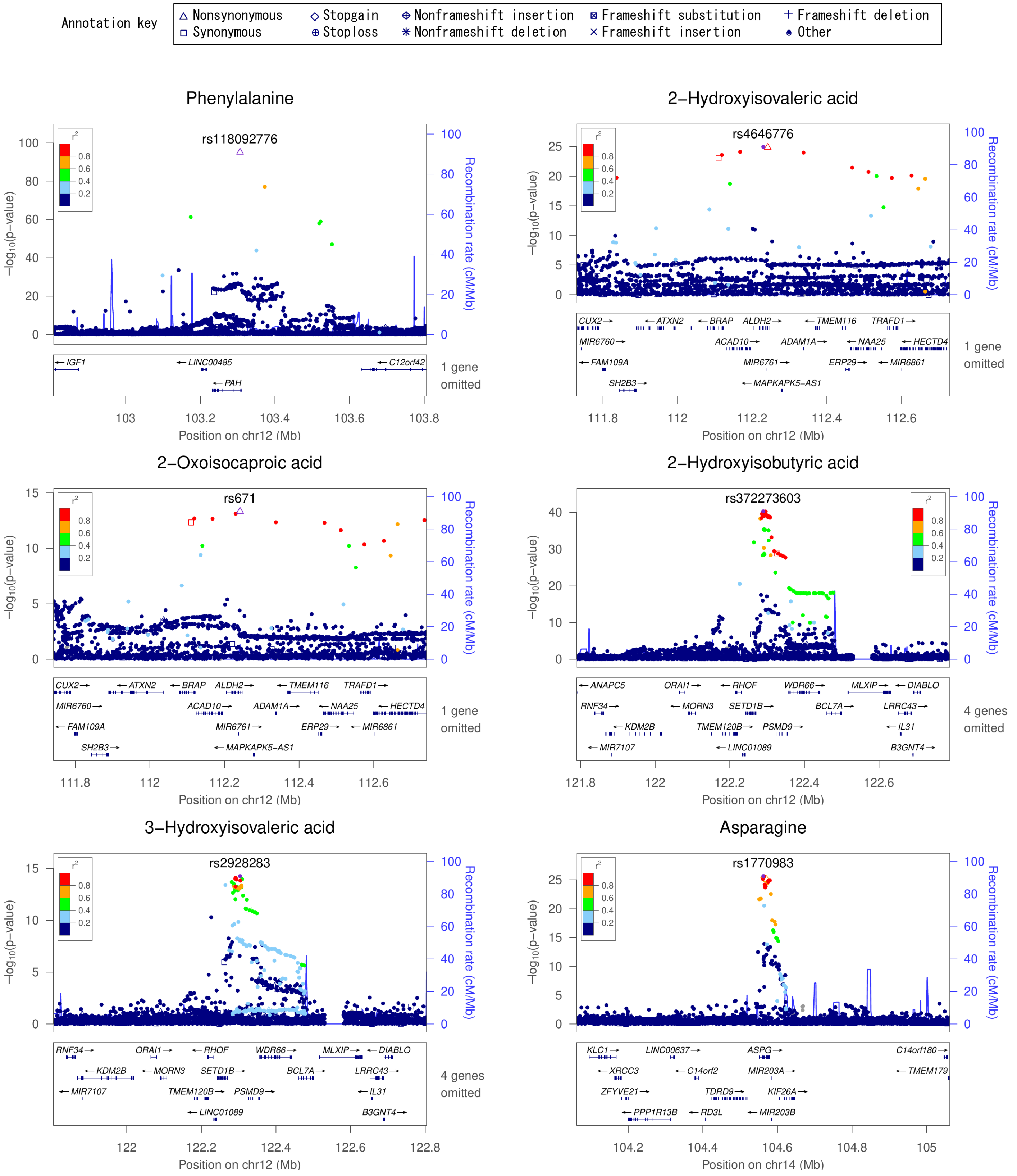

Figure S8. Regional association plots of identified loci in the current study, Related to Table 1

The calculated r^2 with the lead SNP in the 1KG Phase 3 East Asian (EAS) population are shown in color. The gray plots indicate the lead SNP or the corresponding SNP was a monomorphic variant.

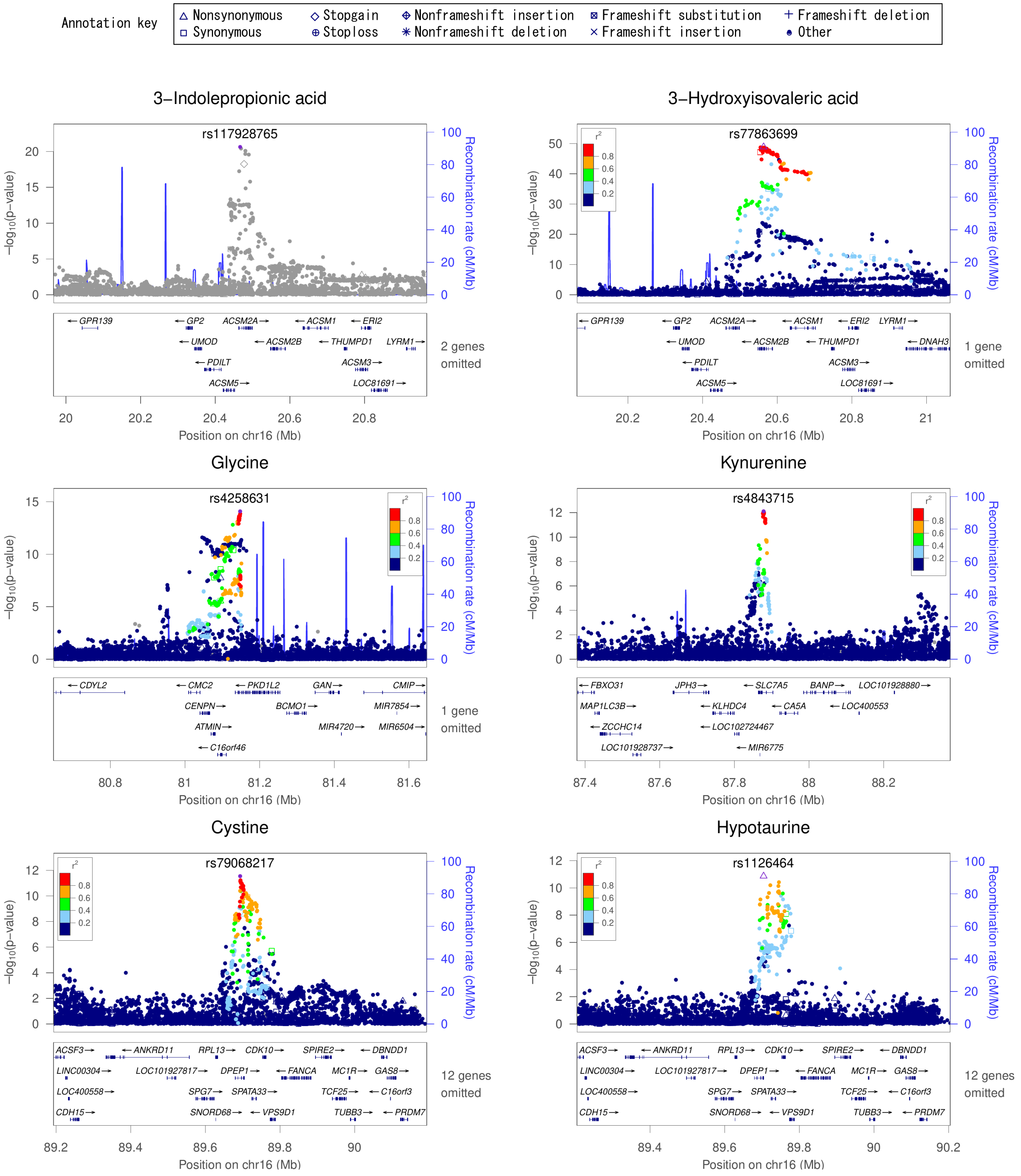

Figure S9. Regional association plots of identified loci in the current study, Related to Table 1

The calculated r^2 with the lead SNP in the 1KG Phase 3 East Asian (EAS) population are shown in color. The gray plots indicate the lead SNP or the corresponding SNP was a monomorphic variant.

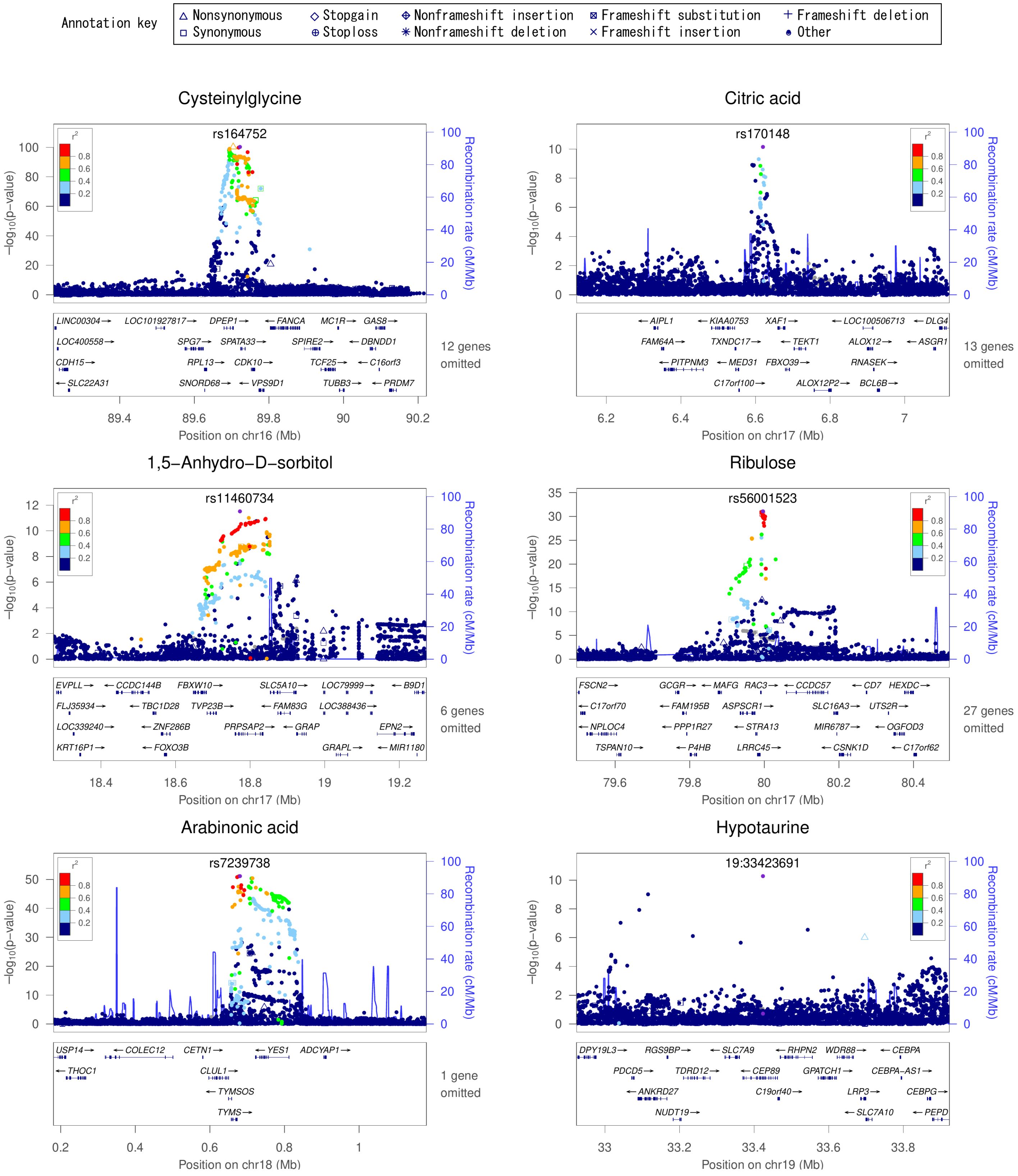

Figure S10. Regional association plots of identified loci in the current study, Related to Table 1

The calculated r^2 with the lead SNP in the 1KG Phase 3 East Asian (EAS) population are shown in color.
The gray plots indicate the lead SNP or the corresponding SNP was a monomorphic variant.

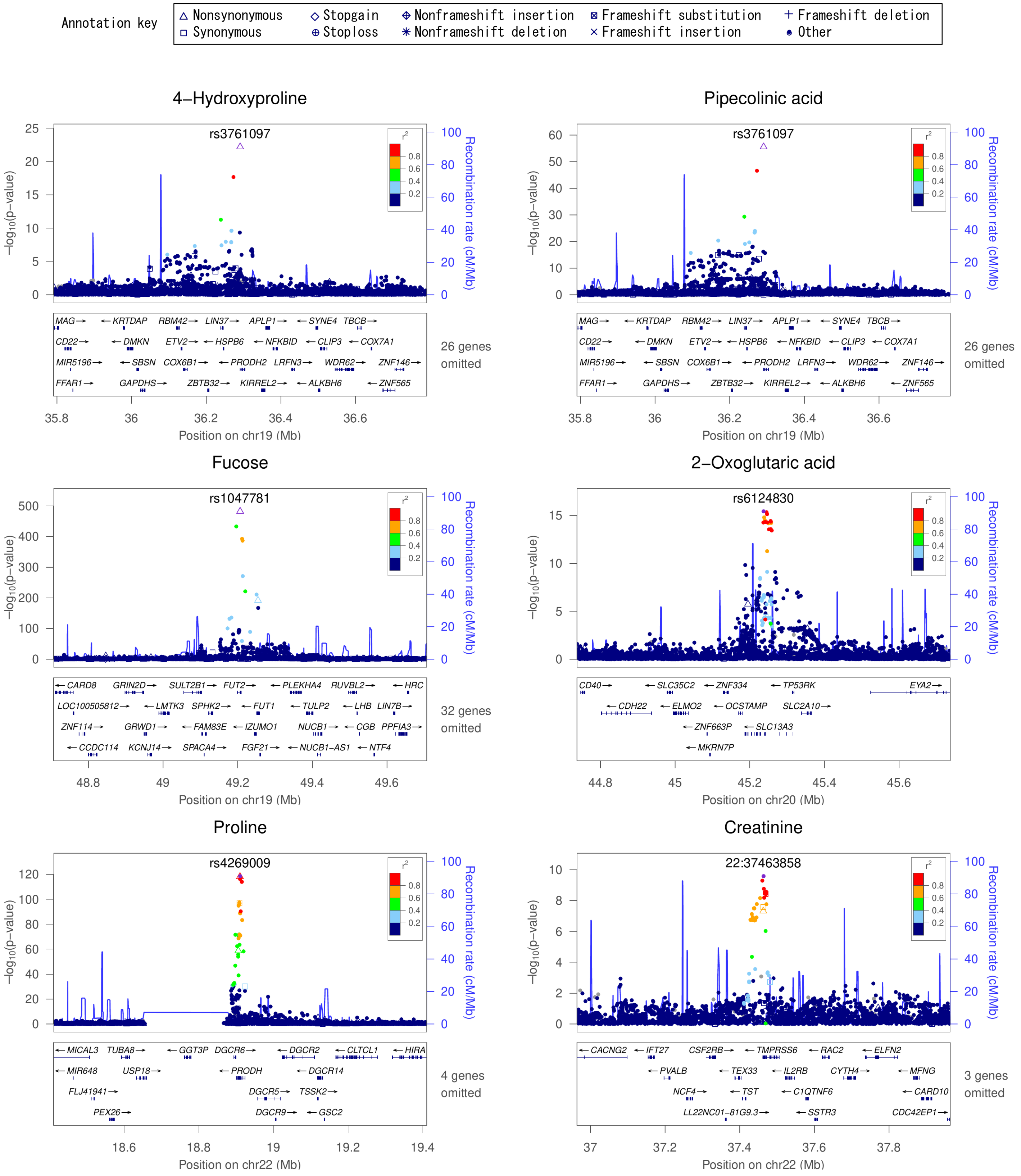

Figure S11. Matrix of metabolites and genes found to be associated, Related to Table 1

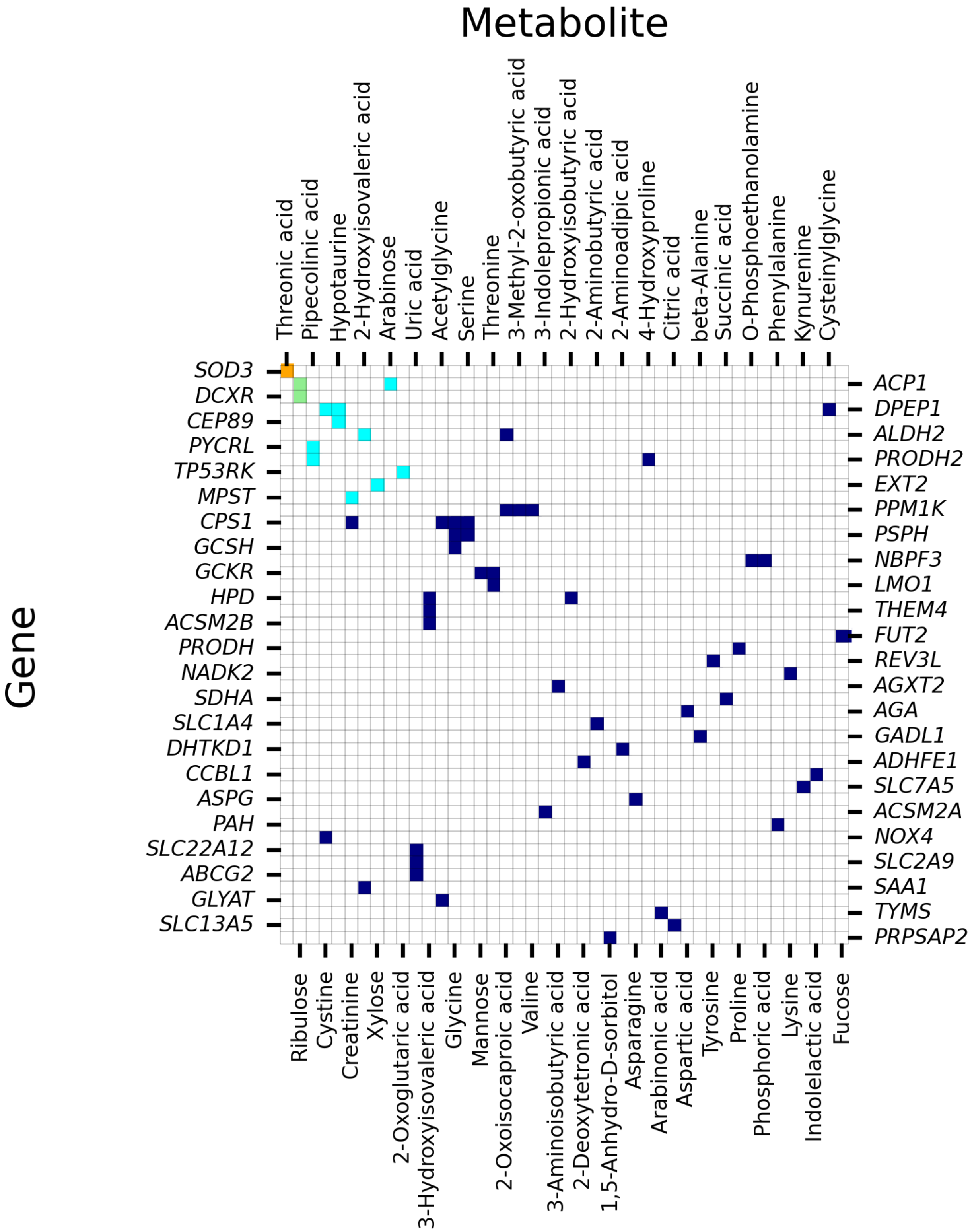

Here, an "association" refers to a genome-wide significant association between a human metabolite and a gene.
Orange: Other associations have been reported for this metabolite, and no association has been reported for this gene.
Green: No association has been reported for this metabolite, and other associations have been reported for this gene.
Cyan: Other associations have also been reported for this metabolite, and other associations have been reported for this gene, but as a pair it is novel.
Navy: This association between metabolites and genes has been previously reported.

Figure S12. Result of conditional analysis, Related to Table 1

The calculated r^2 with the lead SNP in the 1KG Phase3 East Asian (EAS) population are shown in color. The gray plots indicate the lead SNP or the corresponding SNP was a monomorphic variant.

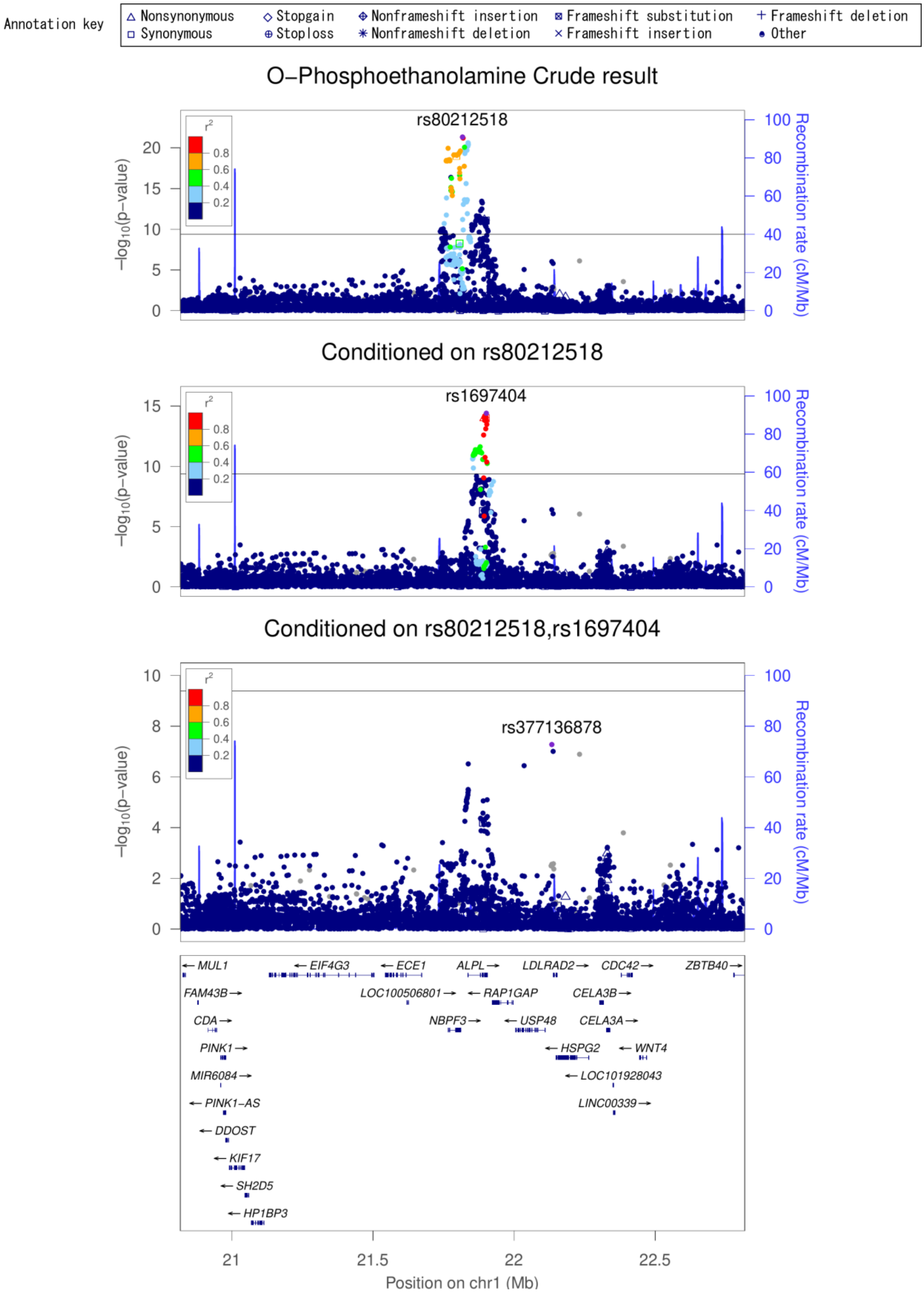

Figure S13. Result of conditional analysis, Related to Table 1

The calculated r^2 with the lead SNP in the 1KG Phase3 East Asian (EAS) population are shown in color. The gray plots indicate the lead SNP or the corresponding SNP was a monomorphic variant.

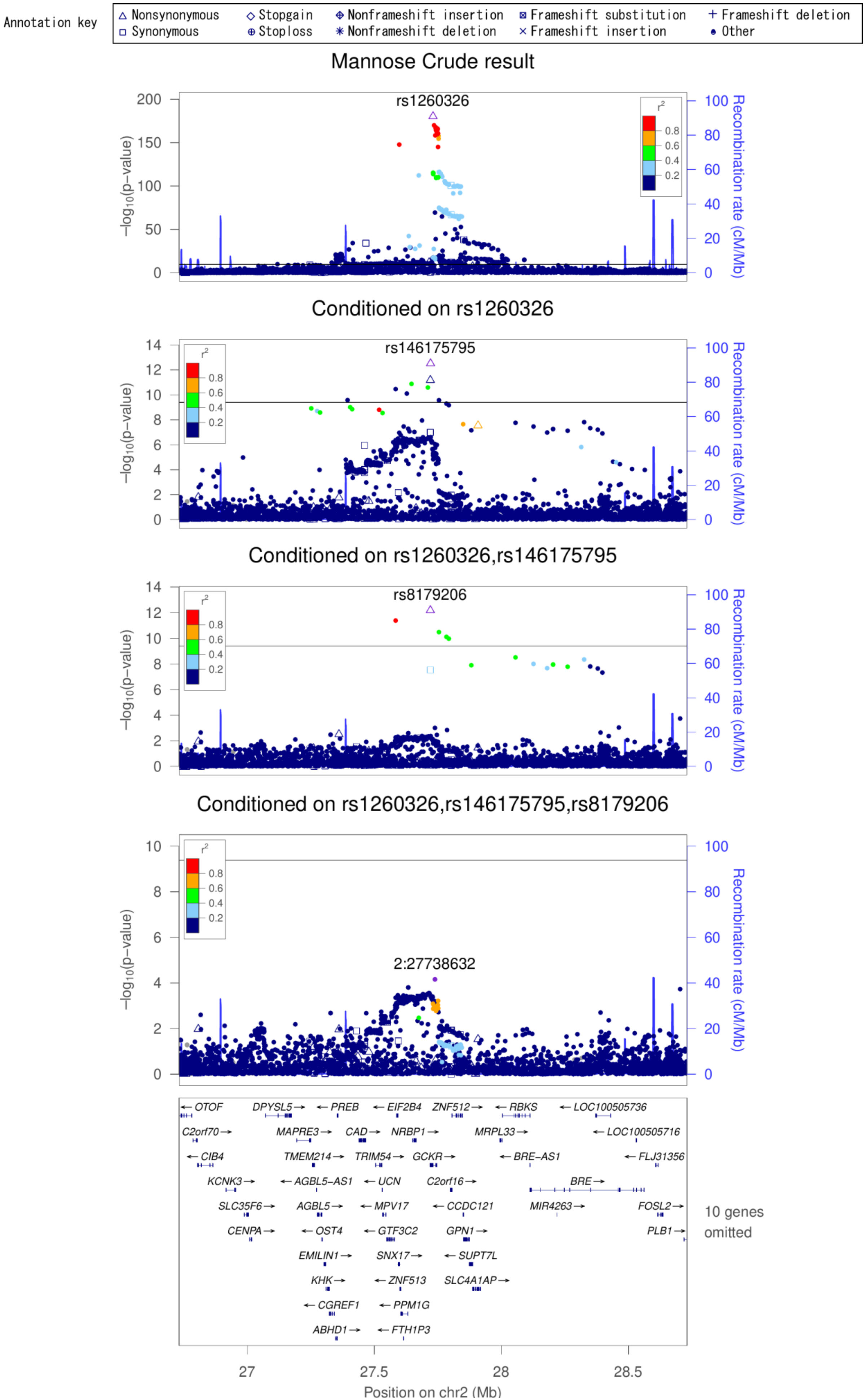

Figure S14. Result of conditional analysis, Related to Table 1

The calculated r^2 with the lead SNP in the 1KG Phase3 East Asian (EAS) population are shown in color. The gray plots indicate the lead SNP or the corresponding SNP was a monomorphic variant.

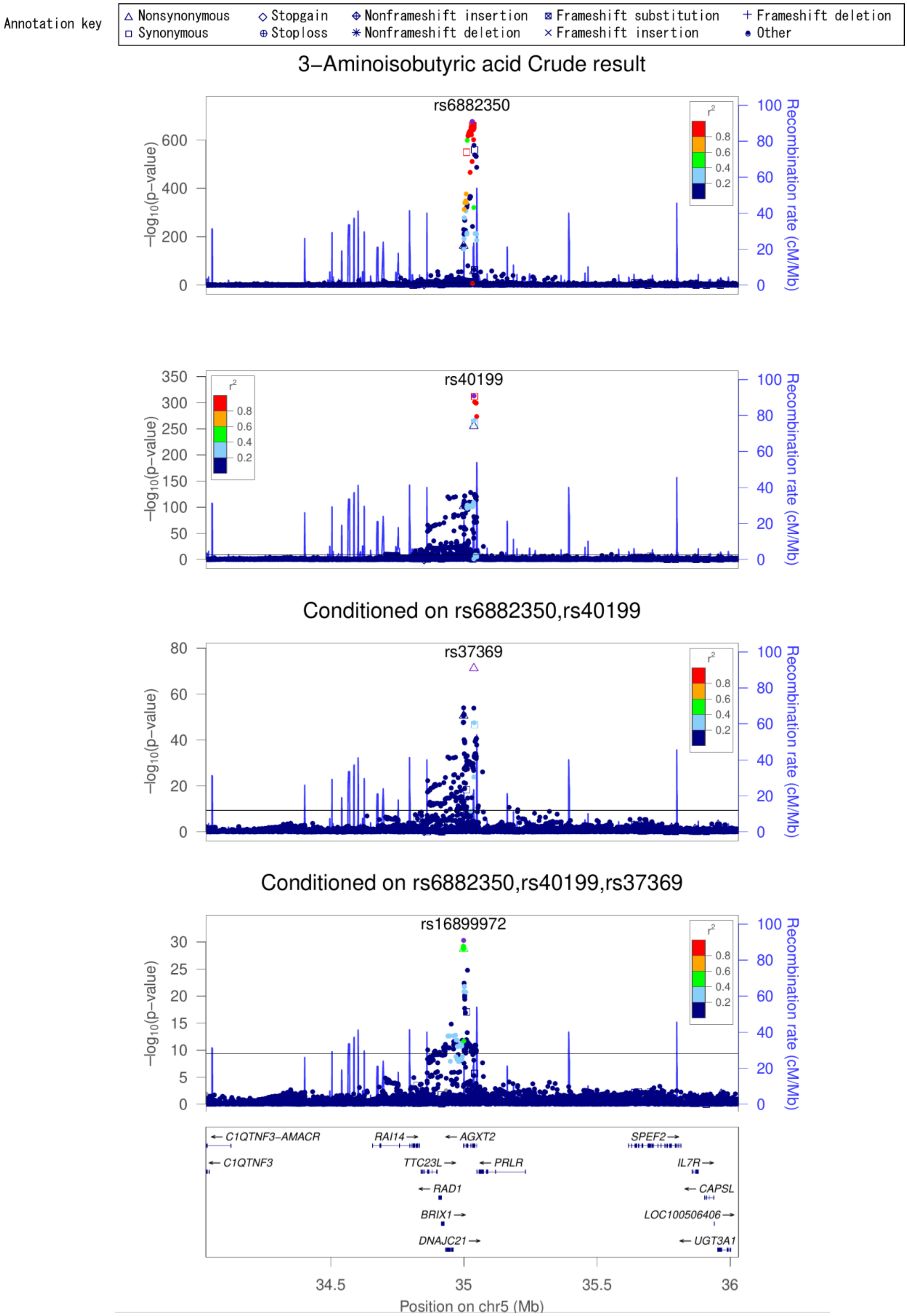

Figure S15. Result of conditional analysis, Related to Table 1

The calculated r^2 with the lead SNP in the 1KG Phase3 East Asian (EAS) population are shown in color. The gray plots indicate the lead SNP or the corresponding SNP was a monomorphic variant.

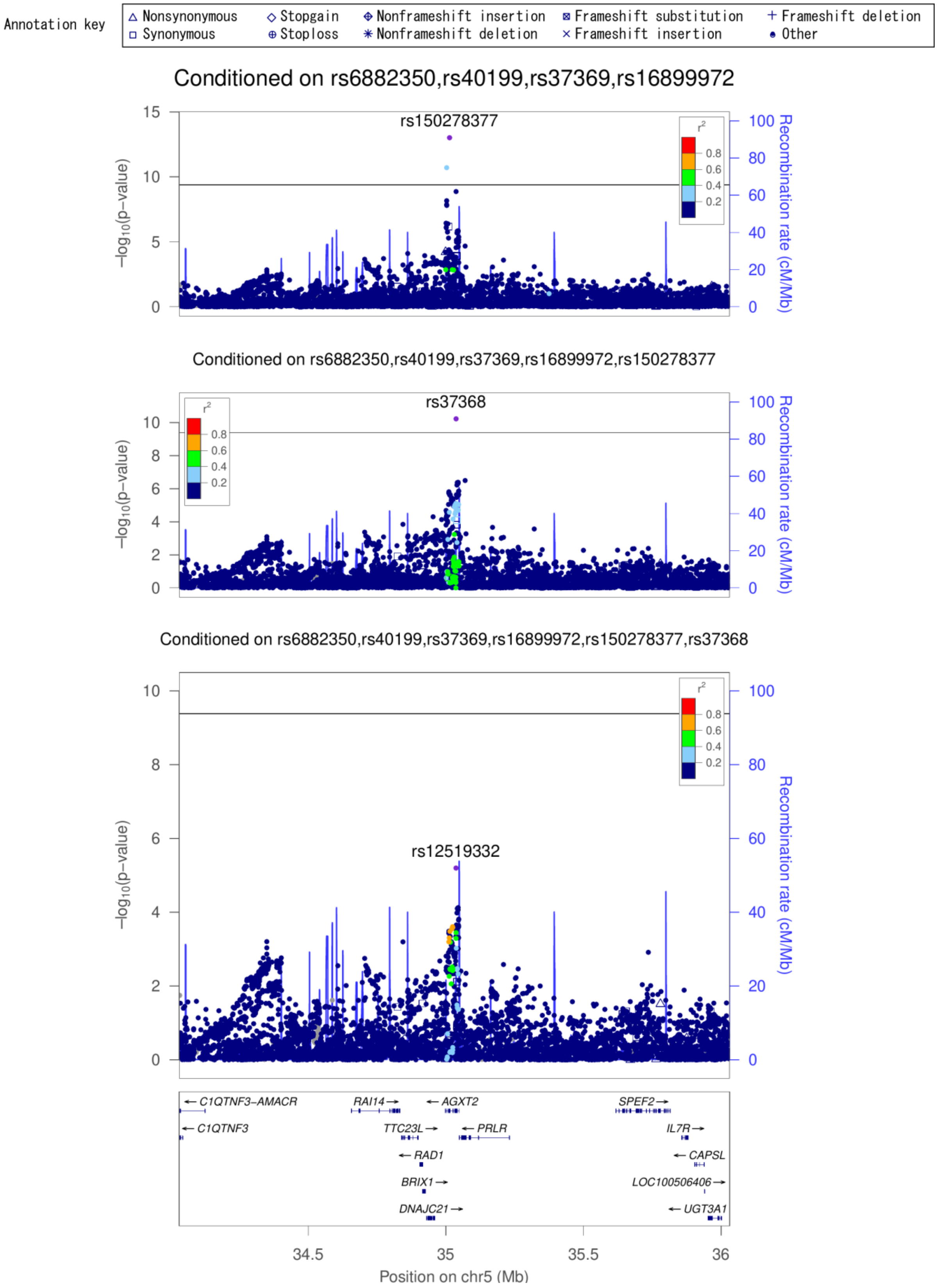

Figure S16. Result of conditional analysis, Related to Table 1

The calculated r^2 with the lead SNP in the 1KG Phase3 East Asian (EAS) population are shown in color. The gray plots indicate the lead SNP or the corresponding SNP was a monomorphic variant.

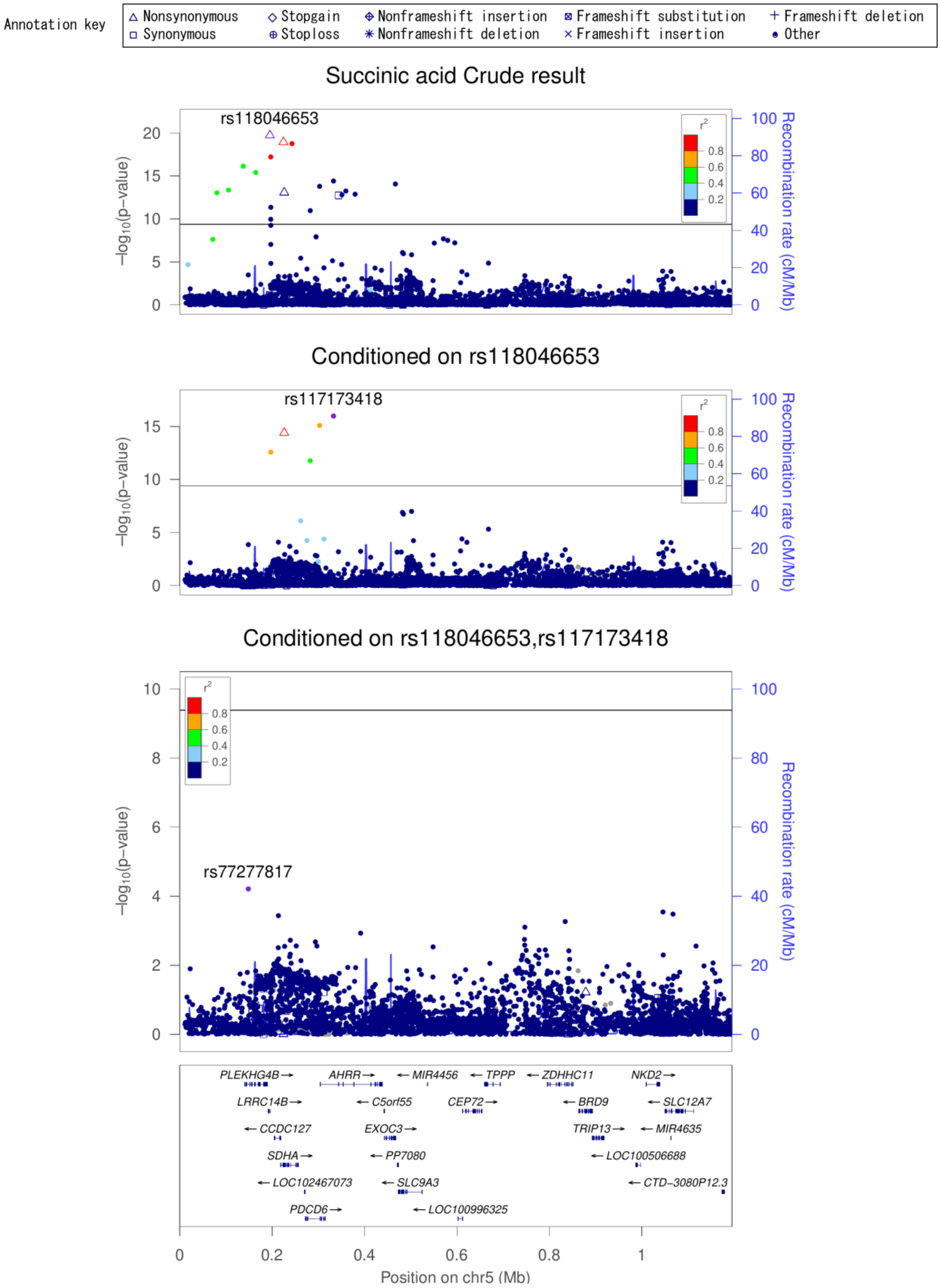

Figure S17. Result of conditional analysis, Related to Table 1

The calculated r^2 with the lead SNP in the 1KG Phase3 East Asian (EAS) population are shown in color. The gray plots indicate the lead SNP or the corresponding SNP was a monomorphic variant.

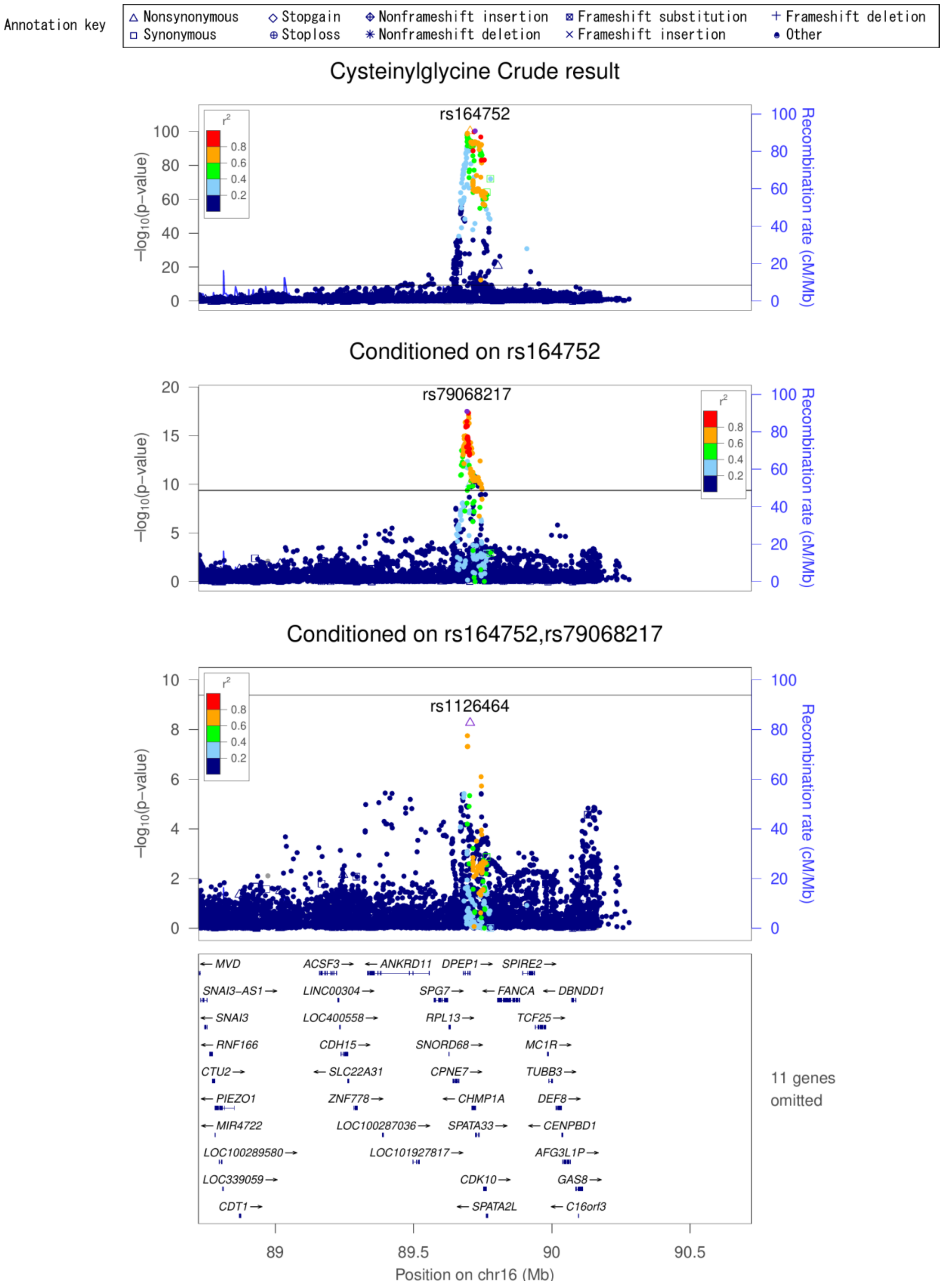

Figure S18. Result of conditional analysis, Related to Table 1

The calculated r^2 with the lead SNP in the 1KG Phase3 East Asian (EAS) population are shown in color. The gray plots indicate the lead SNP or the corresponding SNP was a monomorphic variant.

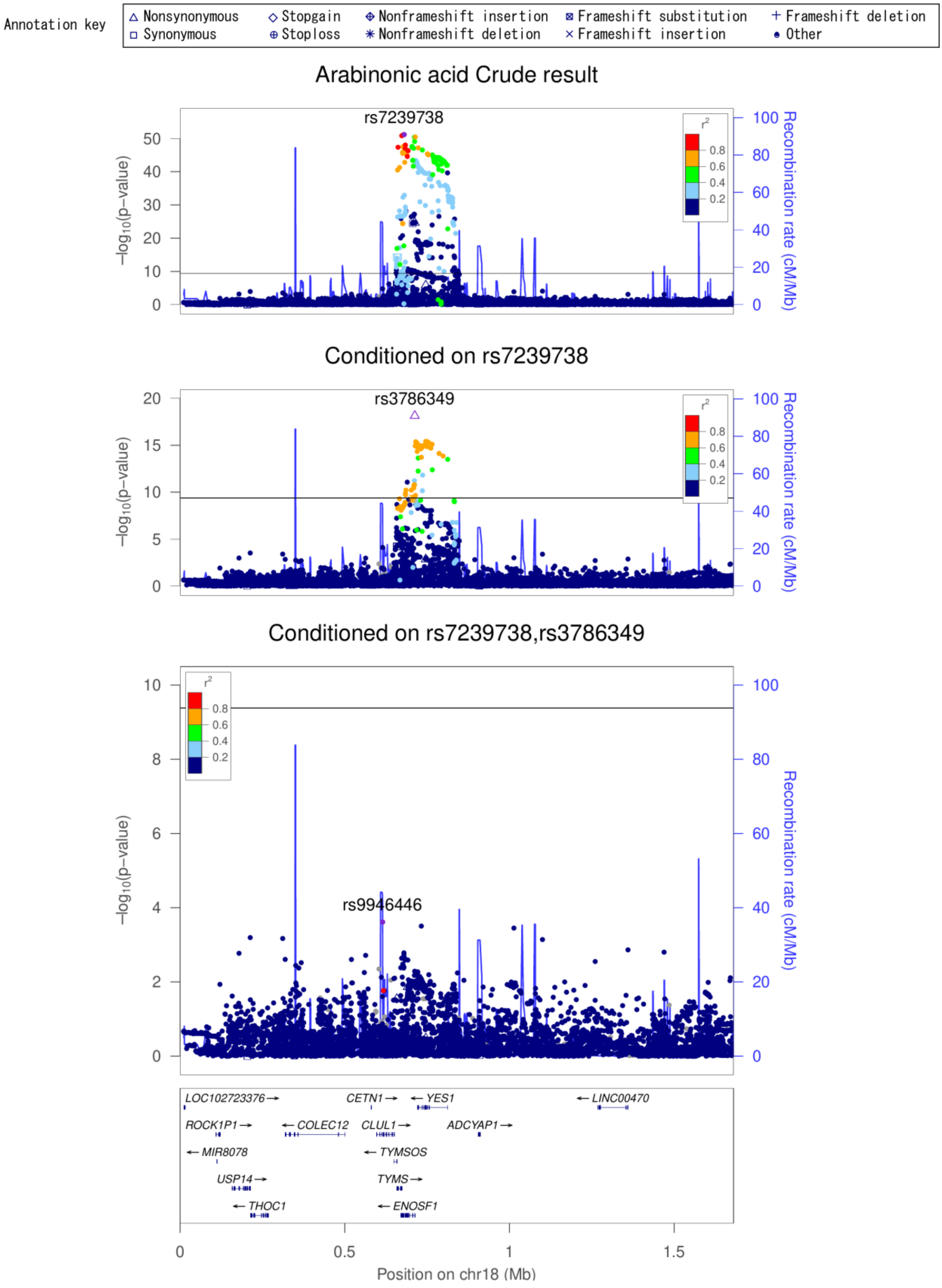

Figure S19. Result of conditional analysis, Related to Table 1

The calculated r^2 with the lead SNP in the 1KG Phase3 East Asian (EAS) population are shown in color. The gray plots indicate the lead SNP or the corresponding SNP was a monomorphic variant.

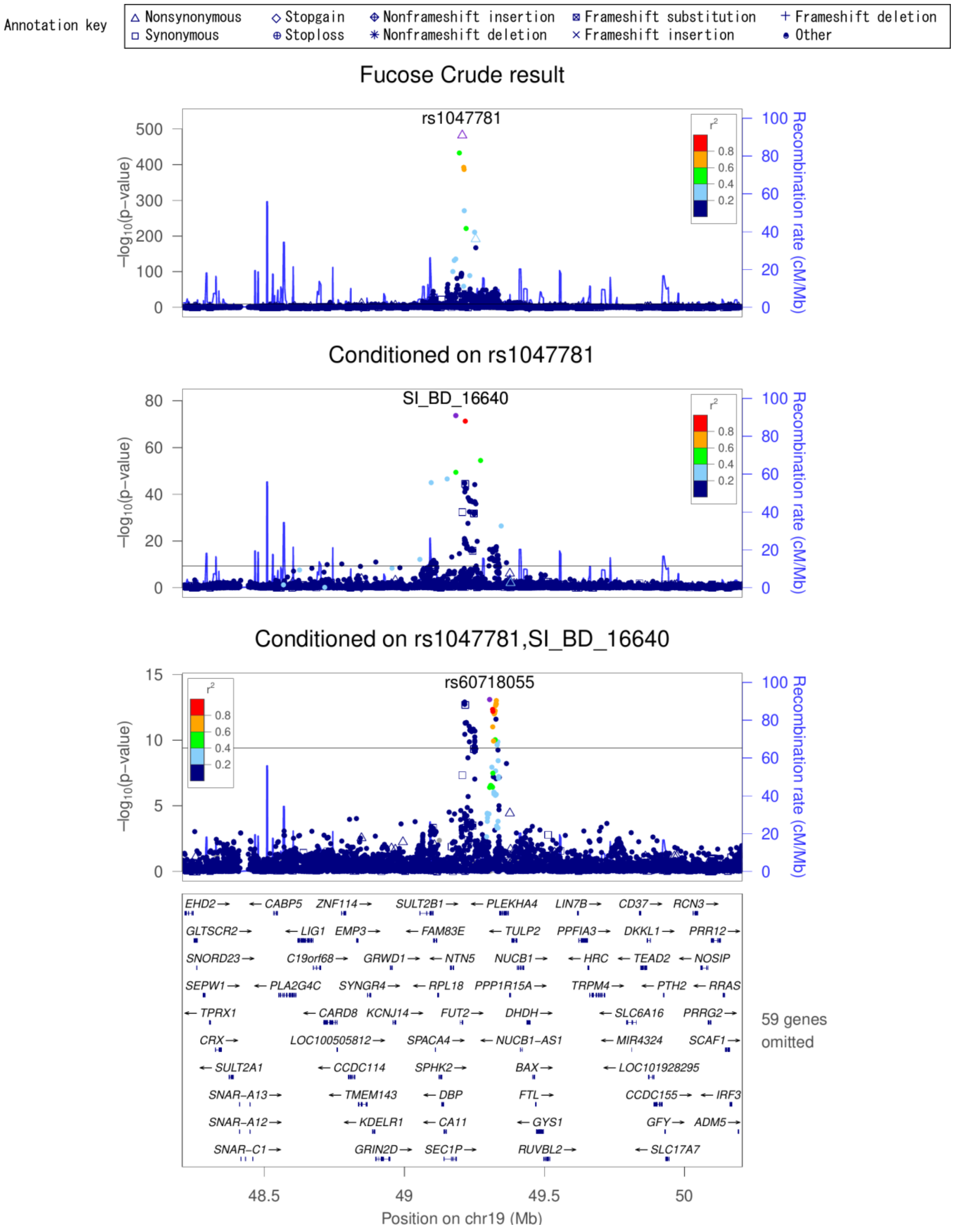

Figure S20. Result of conditional analysis, Related to Table 1

The calculated r^2 with the lead SNP in the 1KG Phase3 East Asian (EAS) population are shown in color. The gray plots indicate the lead SNP or the corresponding SNP was a monomorphic variant.

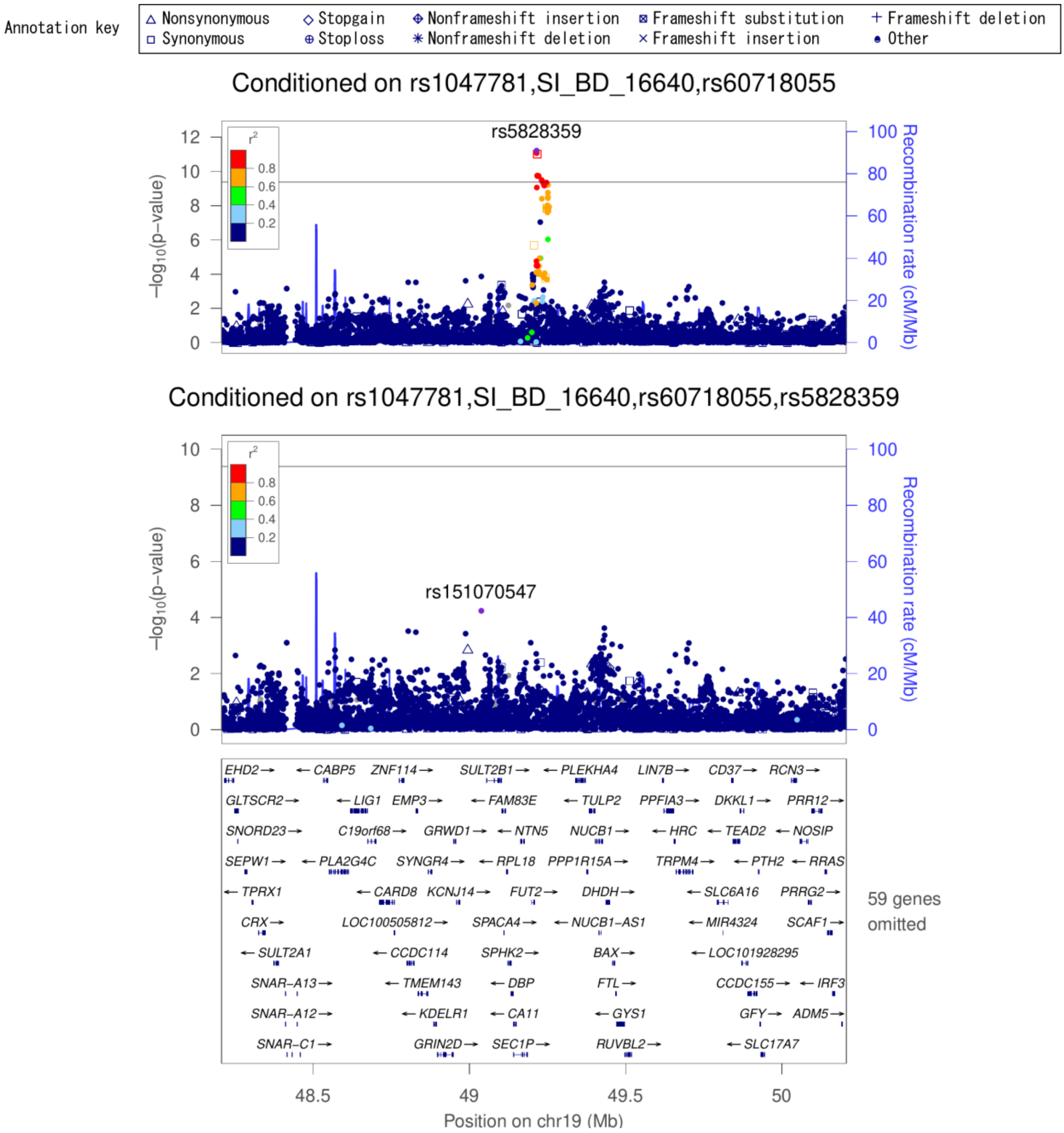

Figure S21. Result of conditional analysis, Related to Table 1

The calculated r^2 with the lead SNP in the 1KG Phase3 East Asian (EAS) population are shown in color. The gray plots indicate the lead SNP or the corresponding SNP was a monomorphic variant.

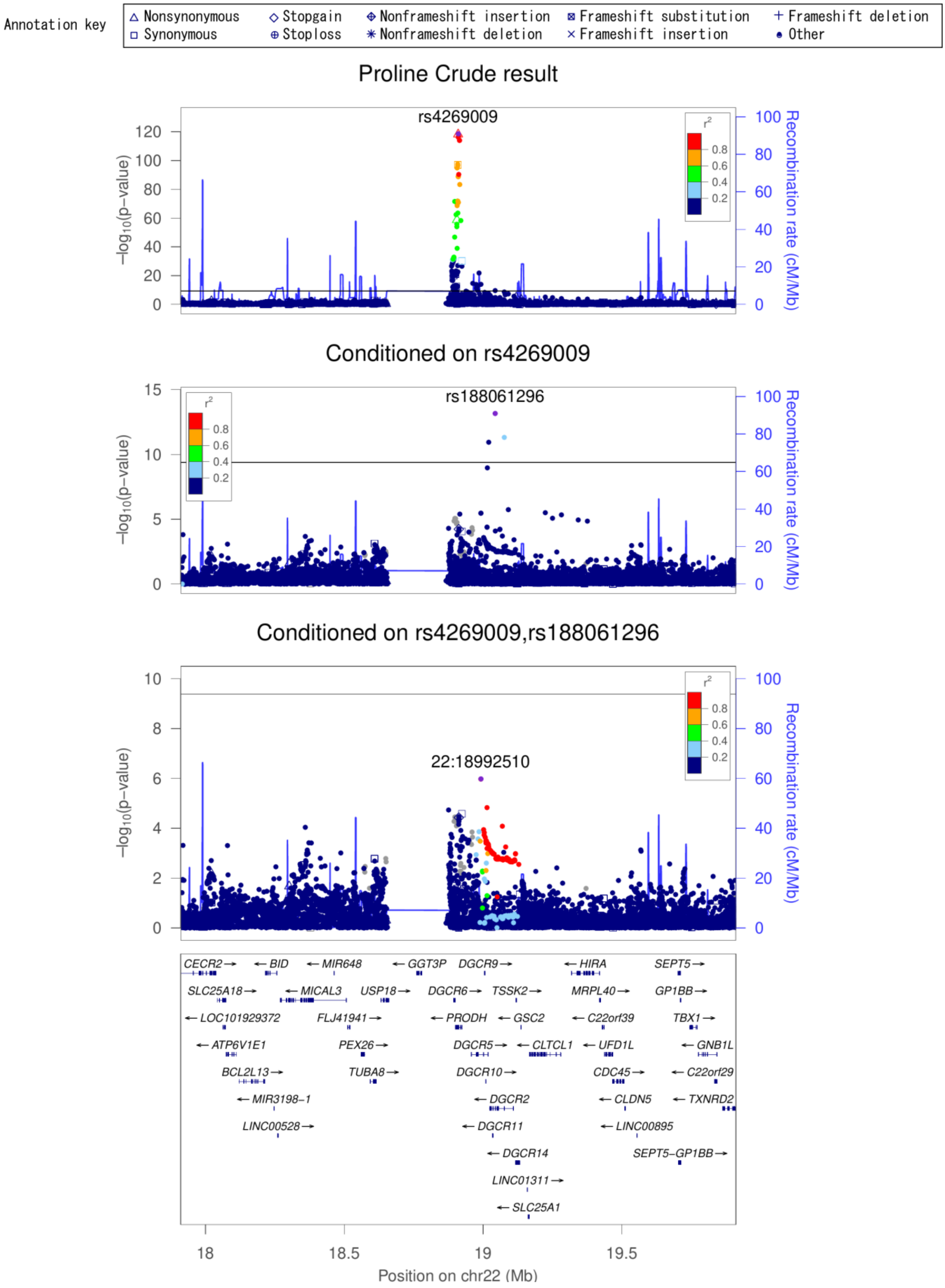

Figure S22. Association between blood metabolite levels and the sum of genotype dosage of the lead and additional SNPs in the same gene, Related to Table 1

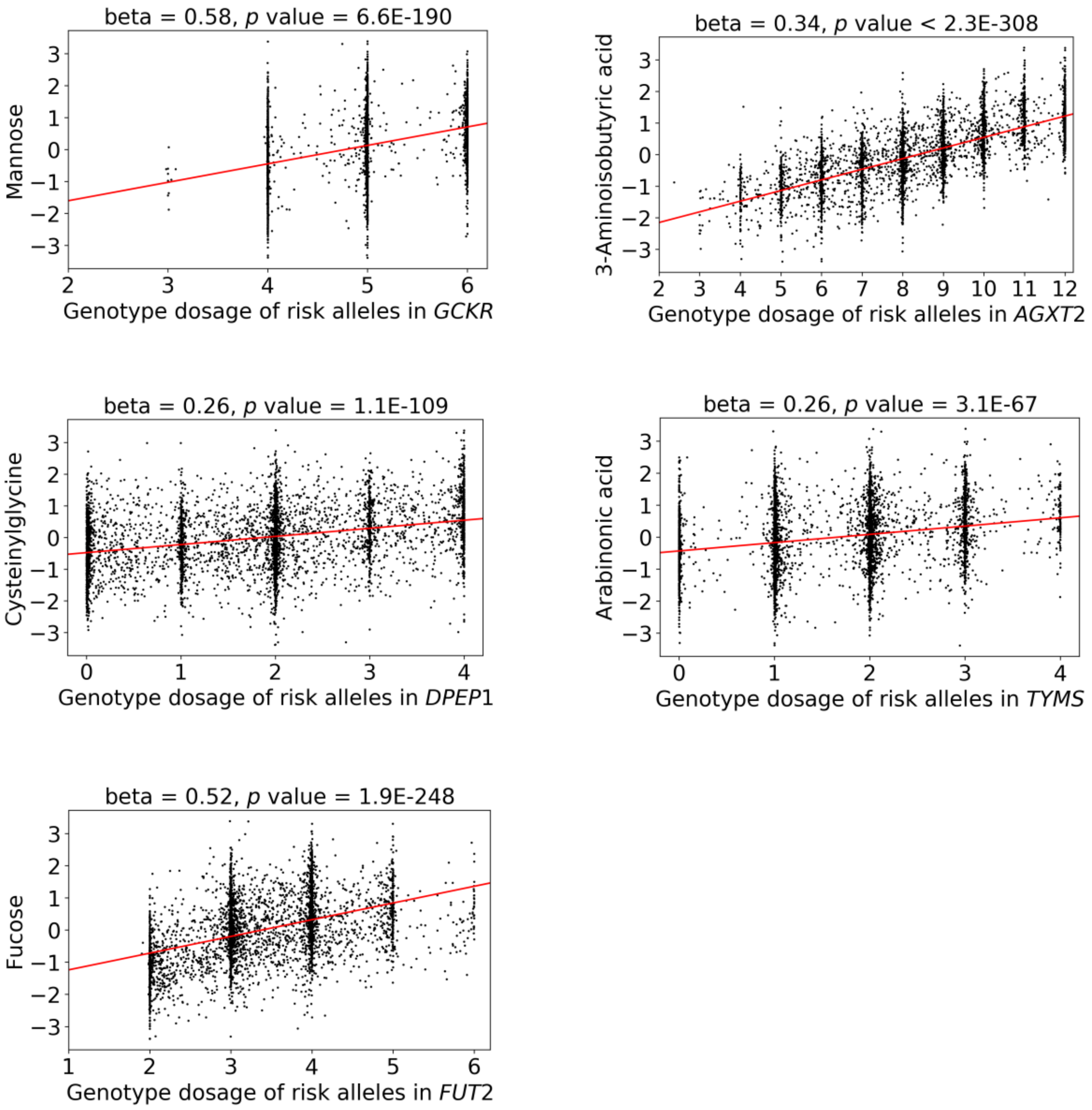

Association test was conducted by the least-squares regression.

Figure S23. The region plots of identified loci in the meta-analysis with Tohoku Medical Megabank Organization (ToMMo), Related to Table 1

The calculated r^2 with the lead SNP in the East Asian (EAS) population (1KG Phase3) are shown in color. The gray plots indicate the lead SNP or the corresponding SNP was a monomorphic variant.

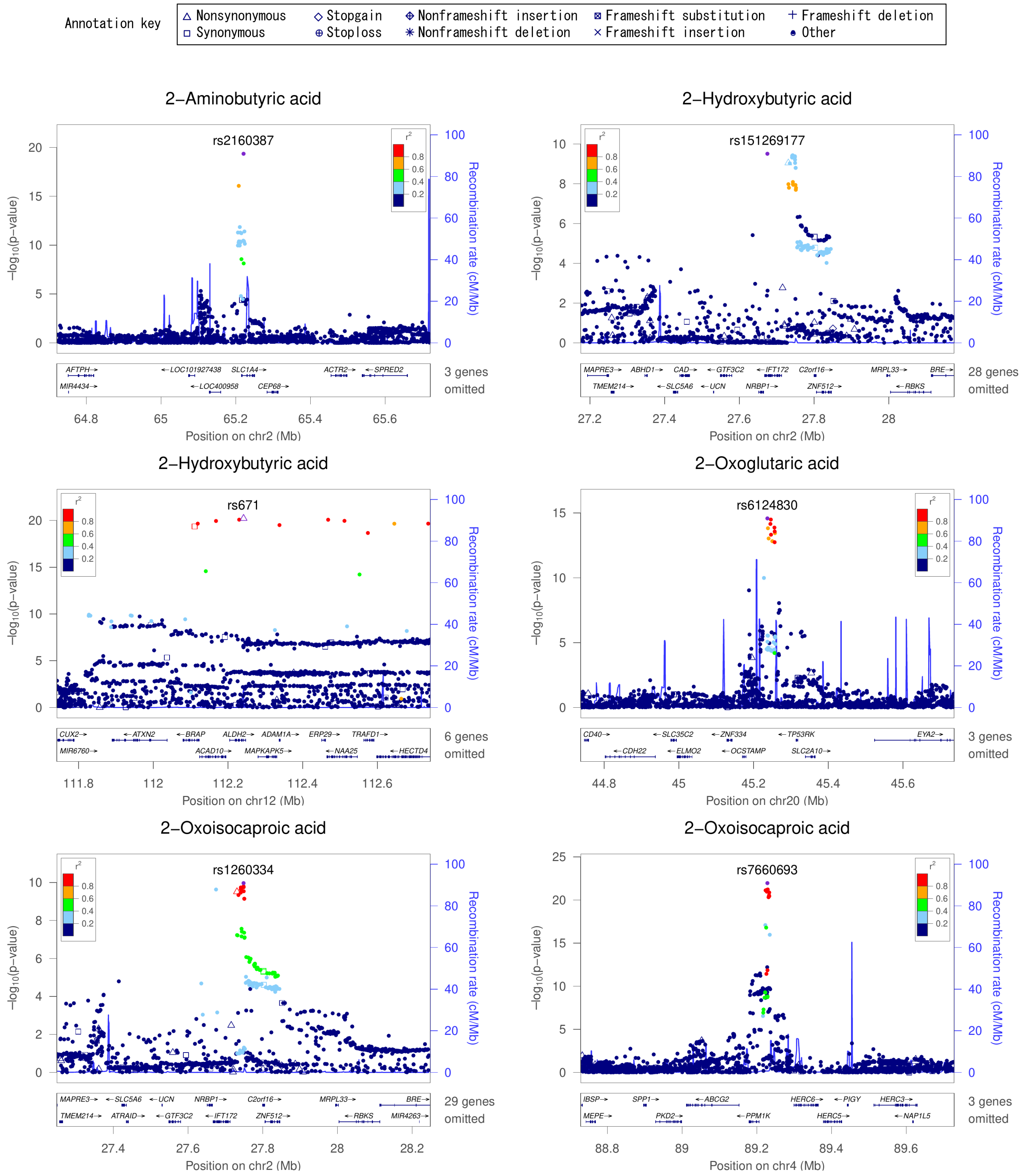

Figure S24. The region plots of identified loci in the meta-analysis with Tohoku Medical Megabank Organization (ToMMo), Related to Table 1

The calculated r^2 with the lead SNP in the East Asian (EAS) population (1KG Phase3) are shown in color. The gray plots indicate the lead SNP or the corresponding SNP was a monomorphic variant.

Annotation key					
	△ Nonsynonymous	◇ Stopgain	⊕ Nonframeshift insertion	⊠ Frameshift substitution	+ Frameshift deletion
	□ Synonymous	⊕ Stoploss	✳ Nonframeshift deletion	× Frameshift insertion	● Other

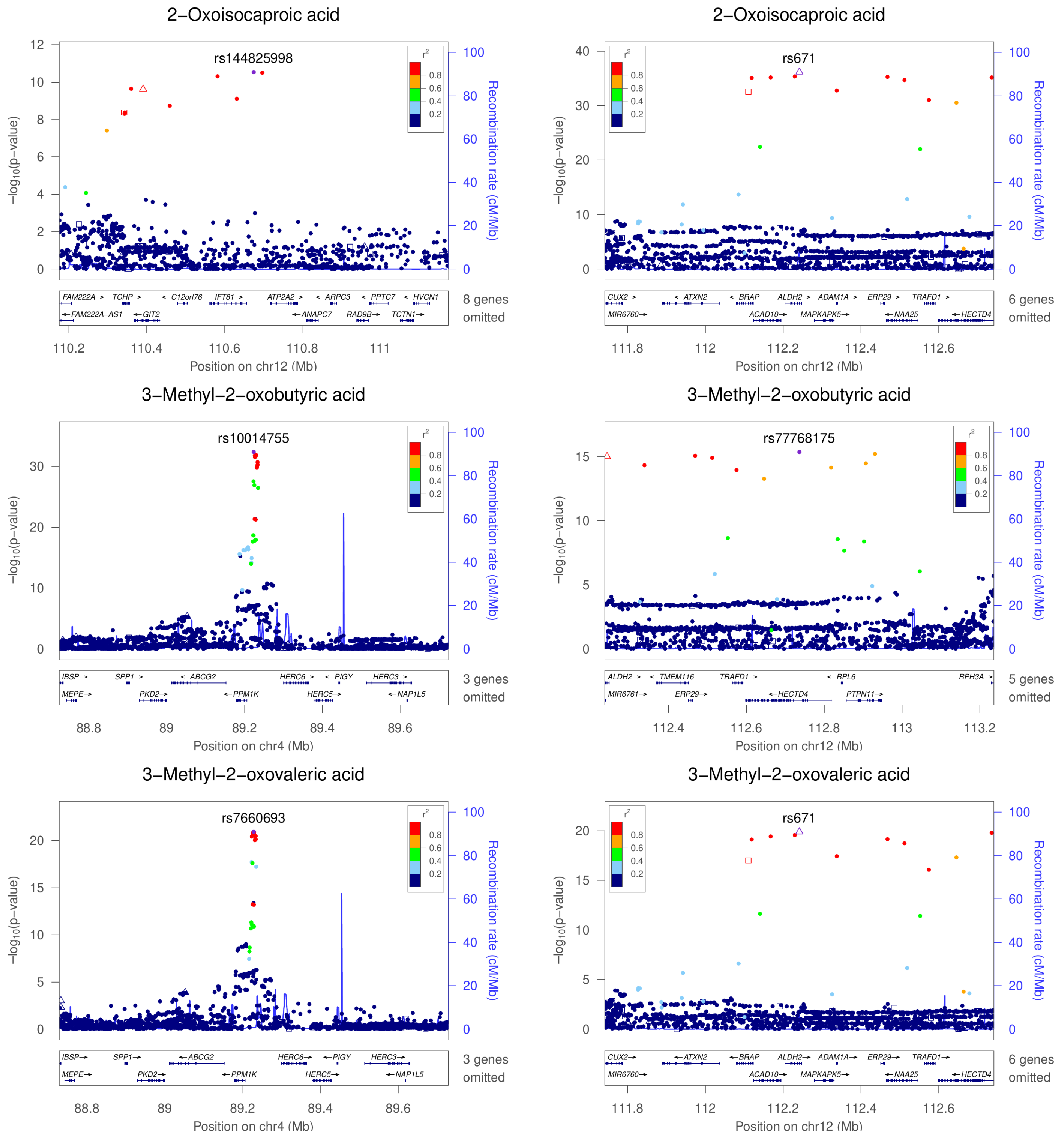

Figure S25. The region plots of identified loci in the meta-analysis with Tohoku Medical Megabank Organization (ToMMo), Related to Table 1

The calculated r^2 with the lead SNP in the East Asian (EAS) population (1KG Phase3) are shown in color. The gray plots indicate the lead SNP or the corresponding SNP was a monomorphic variant.

Annotation key: △ Nonsynonymous; □ Synonymous; ◇ Stopgain; ⊕ Stoploss; Nonframeshift insertion; ✳ Nonframeshift deletion; ⊠ Frameshift substitution; × Frameshift insertion; + Frameshift deletion; ● Other

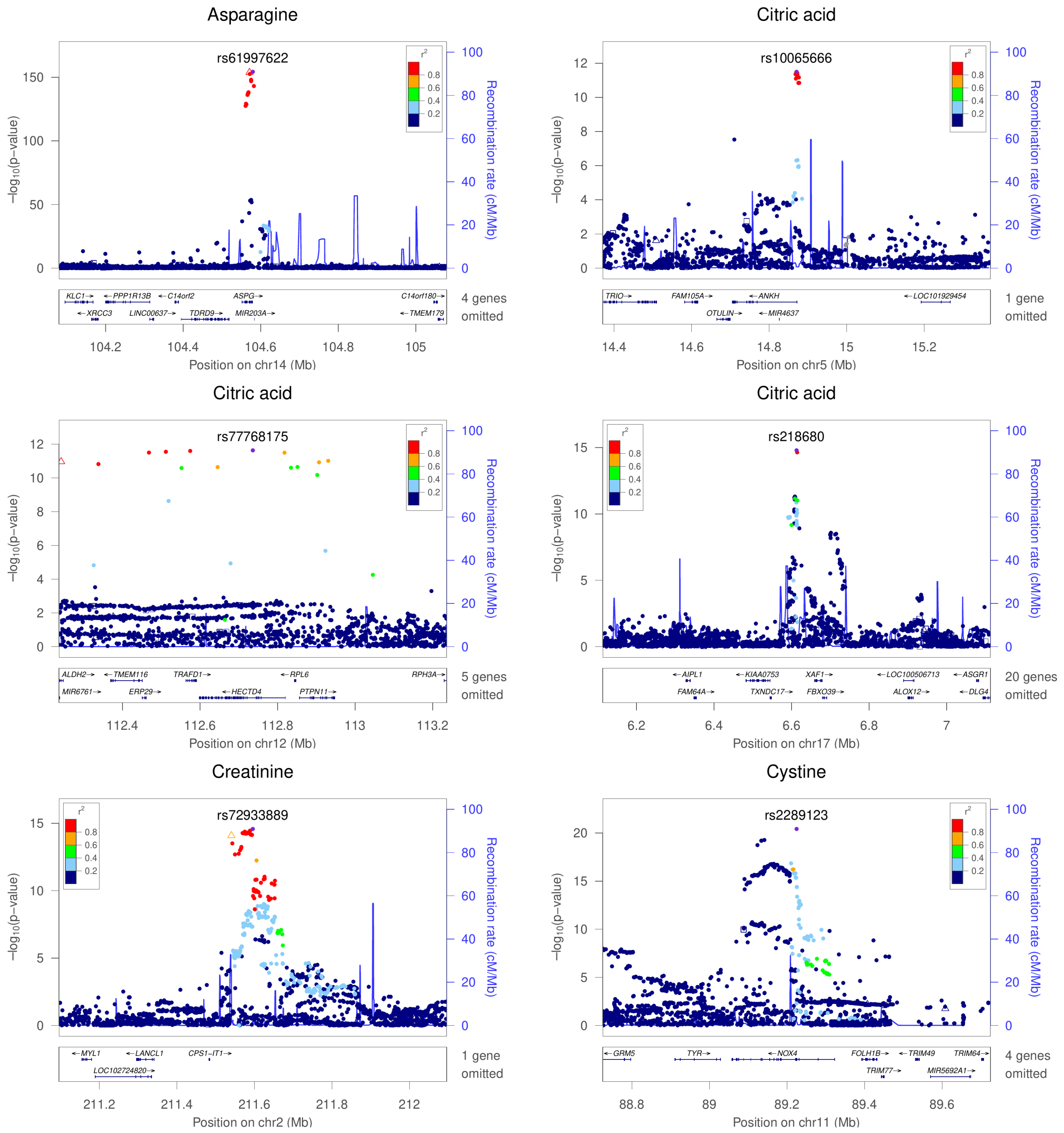

Figure S26. The region plots of identified loci in the meta-analysis with Tohoku Medical Megabank Organization (ToMMo), Related to Table 1

The calculated r^2 with the lead SNP in the East Asian (EAS) population (1KG Phase3) are shown in color. The gray plots indicate the lead SNP or the corresponding SNP was a monomorphic variant.

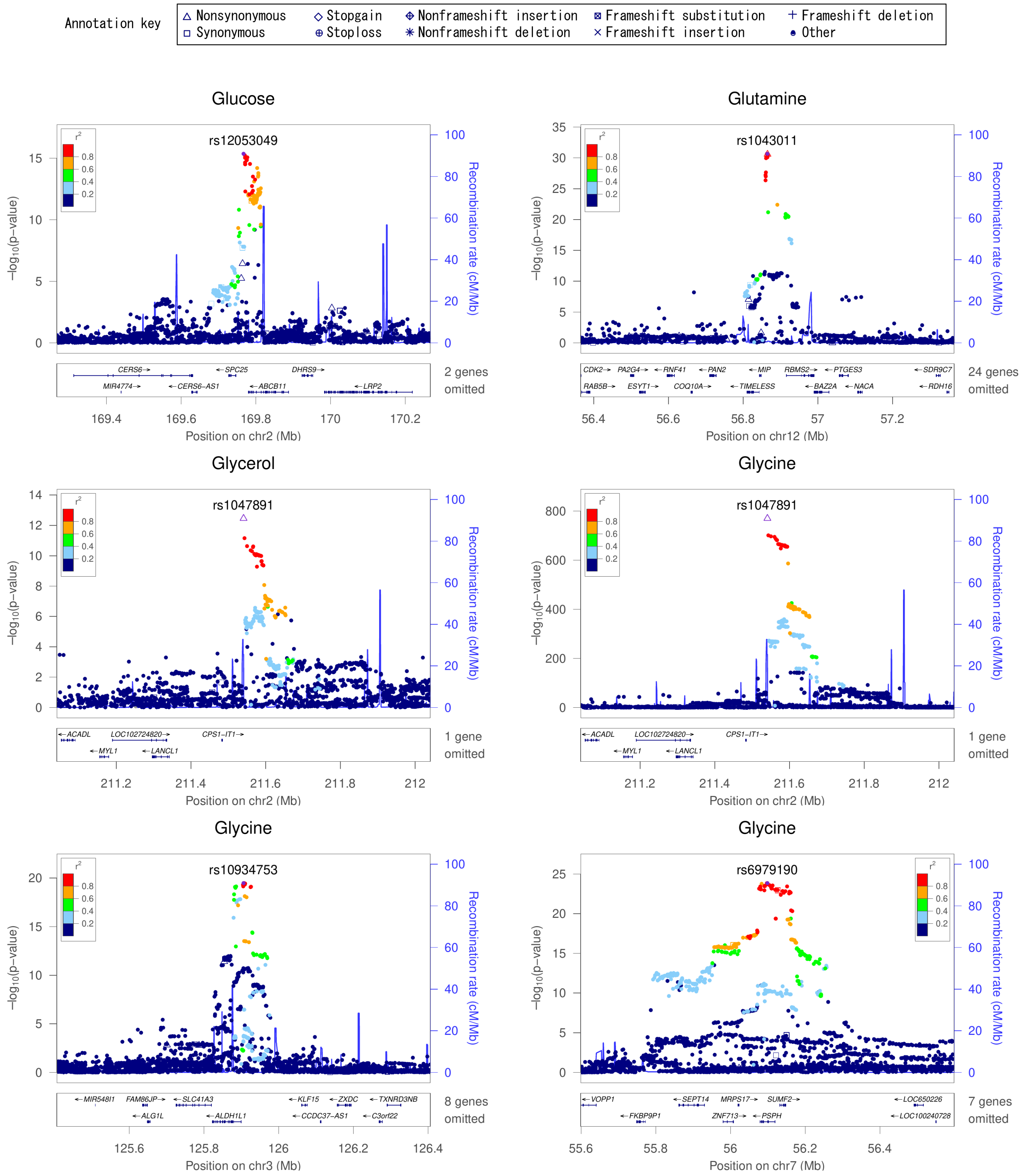

Figure S27. The region plots of identified loci in the meta-analysis with Tohoku Medical Megabank Organization (ToMMo), Related to Table 1

The calculated r^2 with the lead SNP in the East Asian (EAS) population (1KG Phase3) are shown in color. The gray plots indicate the lead SNP or the corresponding SNP was a monomorphic variant.

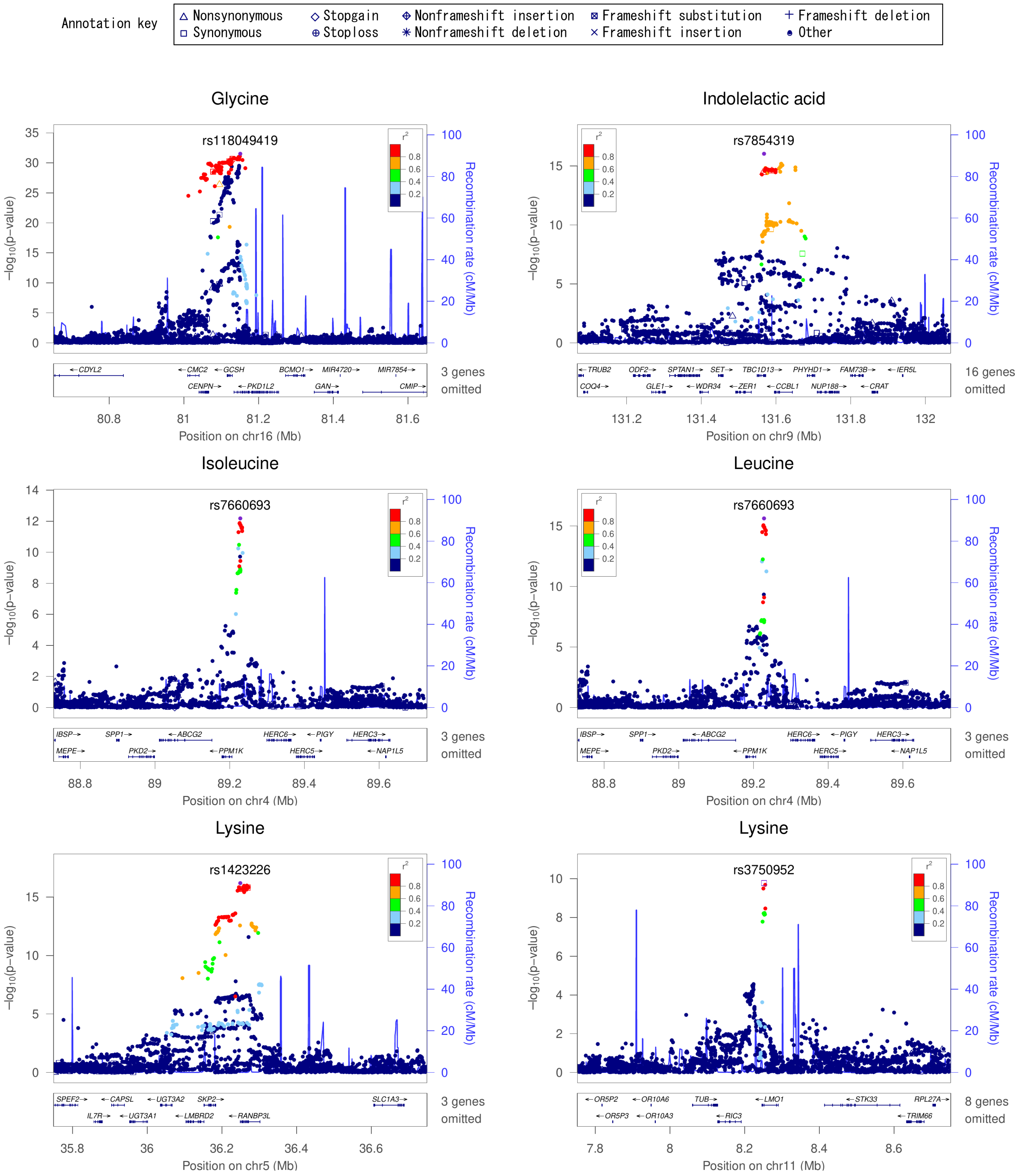

Figure S28. The region plots of identified loci in the meta-analysis with Tohoku Medical Megabank Organization (ToMMo), Related to Table 1

The calculated r^2 with the lead SNP in the East Asian (EAS) population (1KG Phase3) are shown in color. The gray plots indicate the lead SNP or the corresponding SNP was a monomorphic variant.

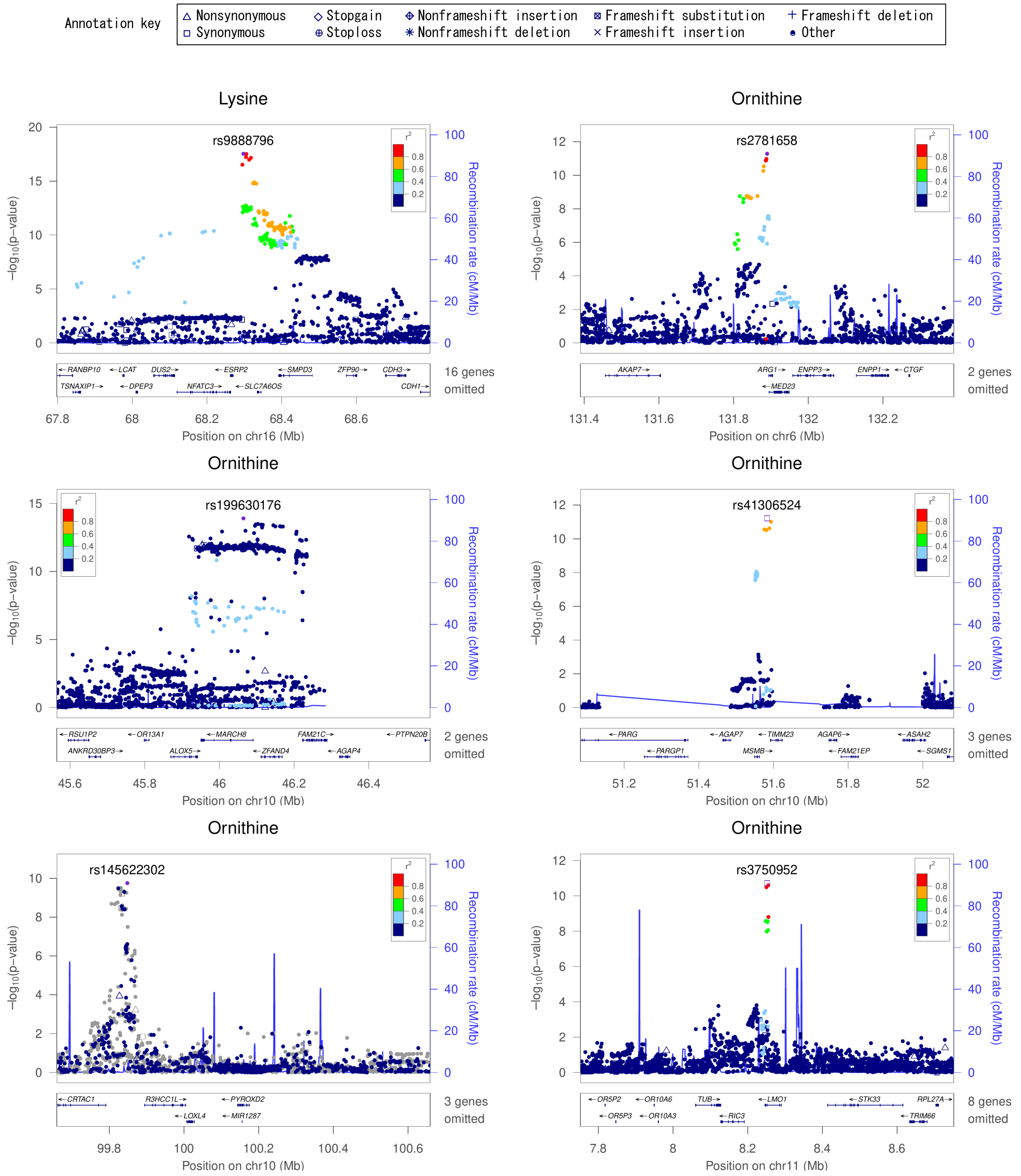

Figure S29. The region plots of identified loci in the meta-analysis with Tohoku Medical Megabank Organization (ToMMo), Related to Table 1

The calculated r^2 with the lead SNP in the East Asian (EAS) population (1KG Phase3) are shown in color. The gray plots indicate the lead SNP or the corresponding SNP was a monomorphic variant.

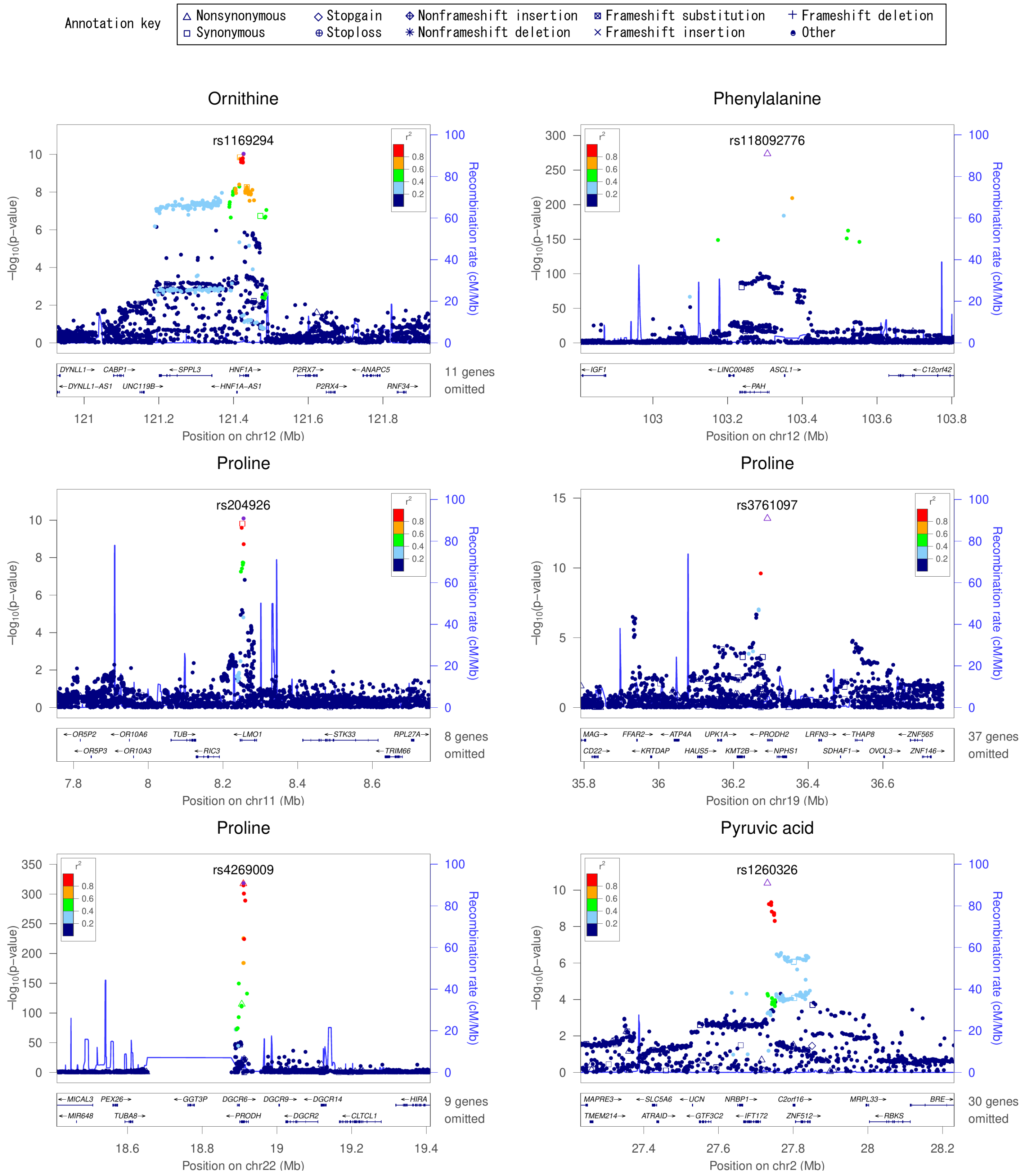

Figure S30. The region plots of identified loci in the meta-analysis with Tohoku Medical Megabank Organization (ToMMo), Related to Table 1

The calculated r^2 with the lead SNP in the East Asian (EAS) population (1KG Phase3) are shown in color. The gray plots indicate the lead SNP or the corresponding SNP was a monomorphic variant.

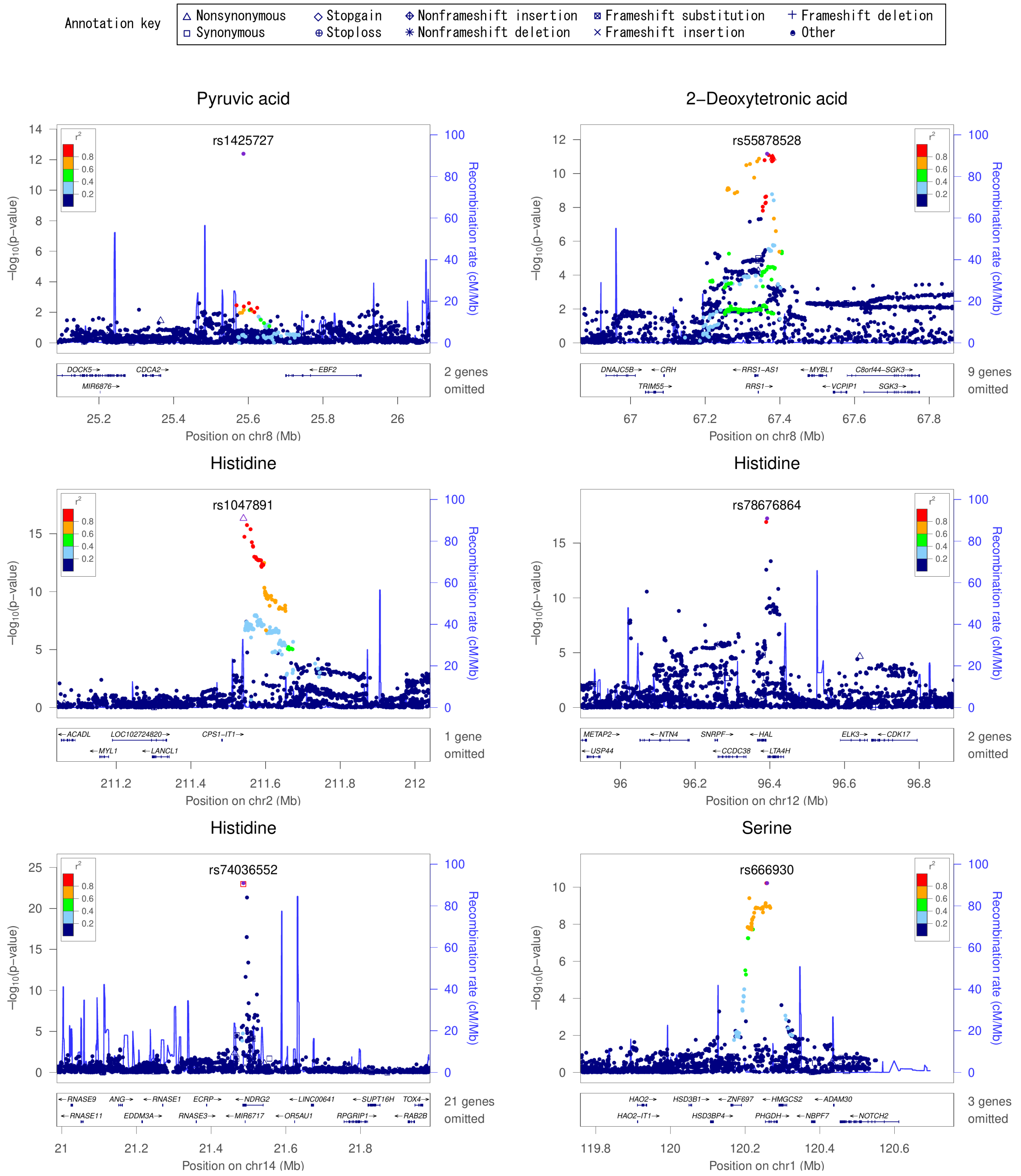

Figure S31. The region plots of identified loci in the meta-analysis with Tohoku Medical Megabank Organization (ToMMo), Related to Table 1

The calculated r^2 with the lead SNP in the East Asian (EAS) population (1KG Phase3) are shown in color. The gray plots indicate the lead SNP or the corresponding SNP was a monomorphic variant.

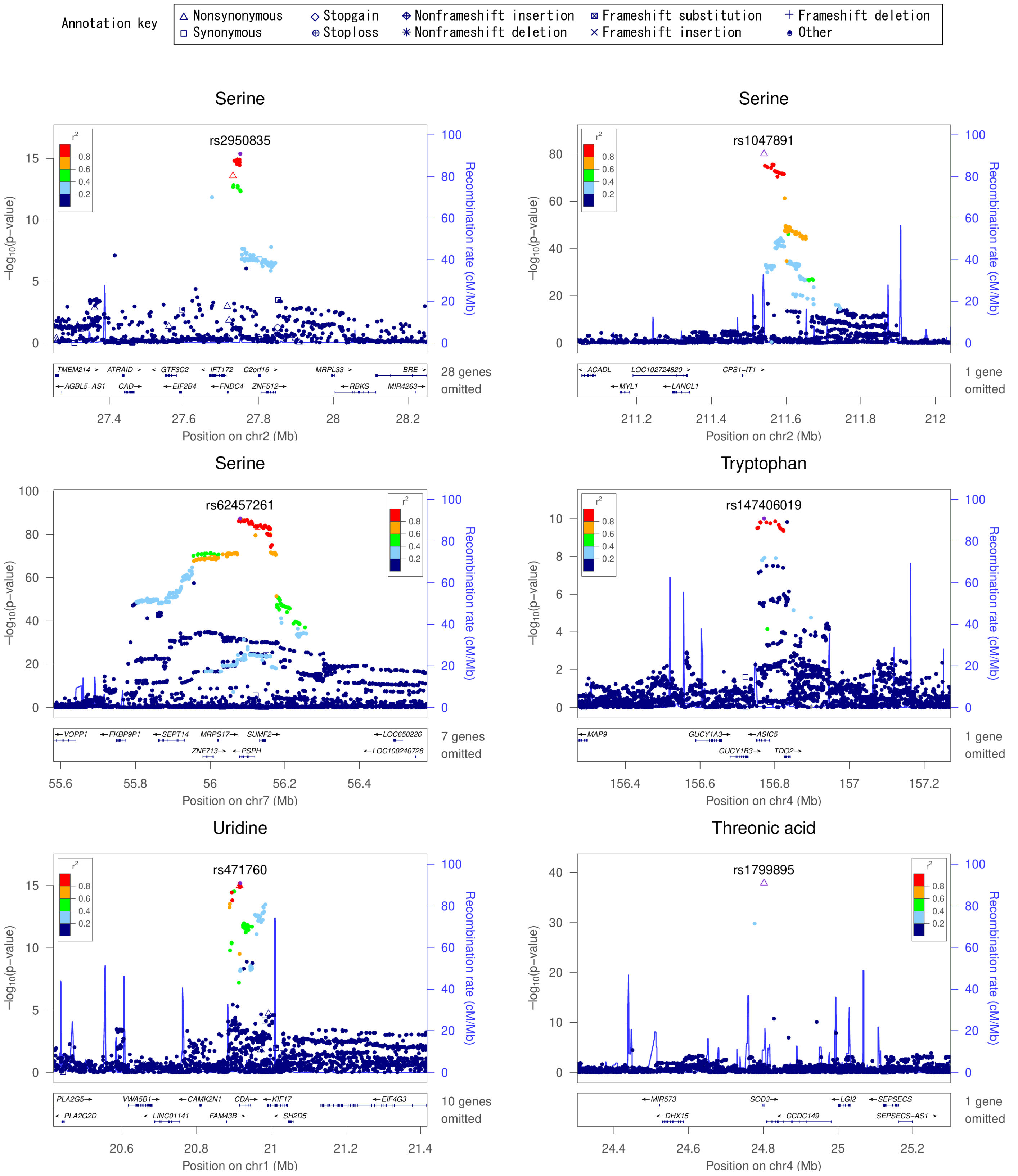

Figure S32. The region plots of identified loci in the meta-analysis with Tohoku Medical Megabank Organization (ToMMo), Related to Table 1

The calculated r^2 with the lead SNP in the East Asian (EAS) population (1KG Phase3) are shown in color. The gray plots indicate the lead SNP or the corresponding SNP was a monomorphic variant.

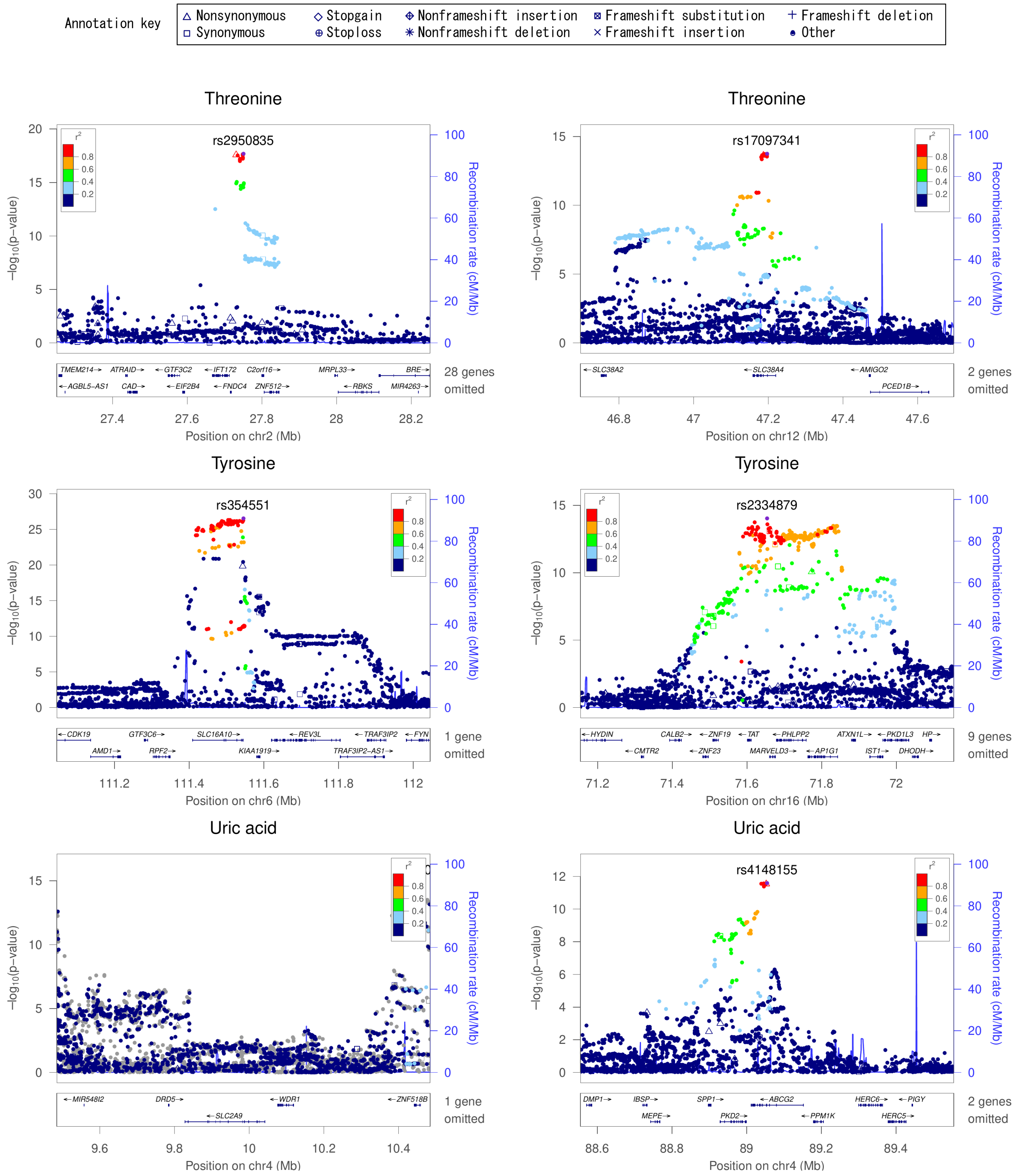

Figure S33. The region plots of identified loci in the meta-analysis with Tohoku Medical Megabank Organization (ToMMo), Related to Table 1

The calculated r^2 with the lead SNP in the East Asian (EAS) population (1KG Phase3) are shown in color. The gray plots indicate the lead SNP or the corresponding SNP was a monomorphic variant.

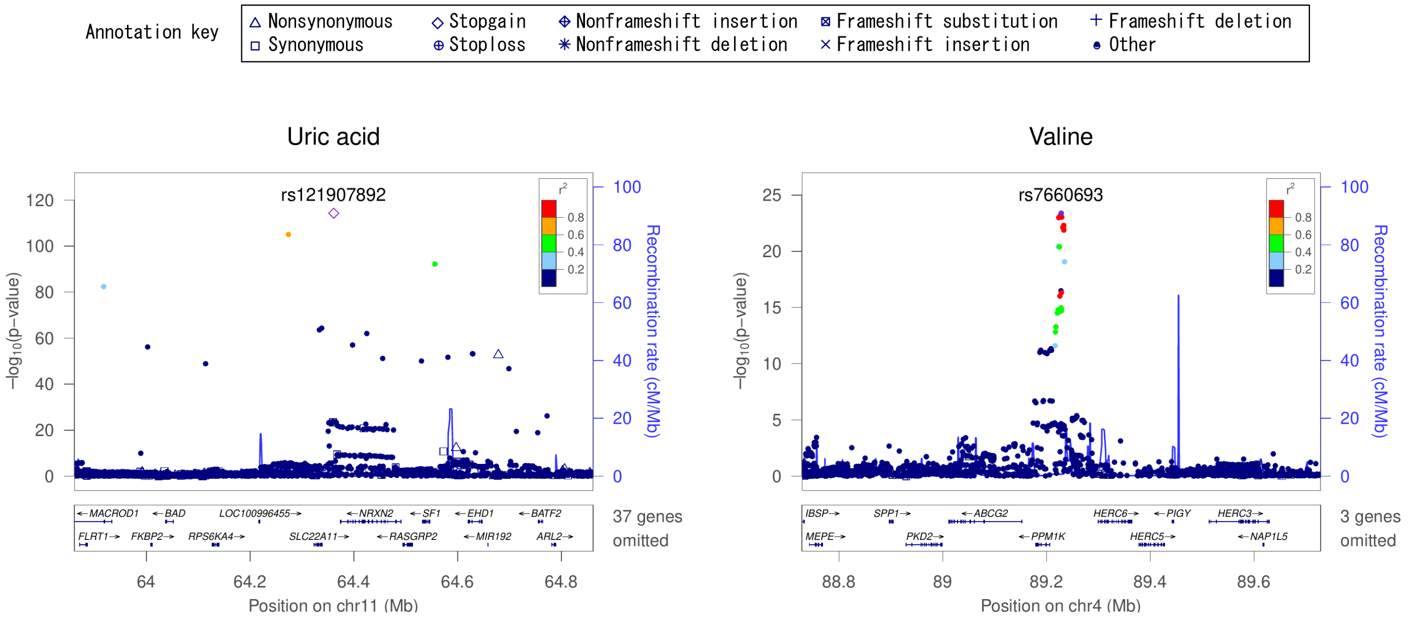

Figure S34. The region plots of identified loci in the multi-population meta-analysis, Related to Table 1

The calculated r^2 with the lead SNP in the East Asian (EAS) population (1KG Phase3) are shown in color. The gray plots indicate the lead SNP or the corresponding SNP was a monomorphic variant.

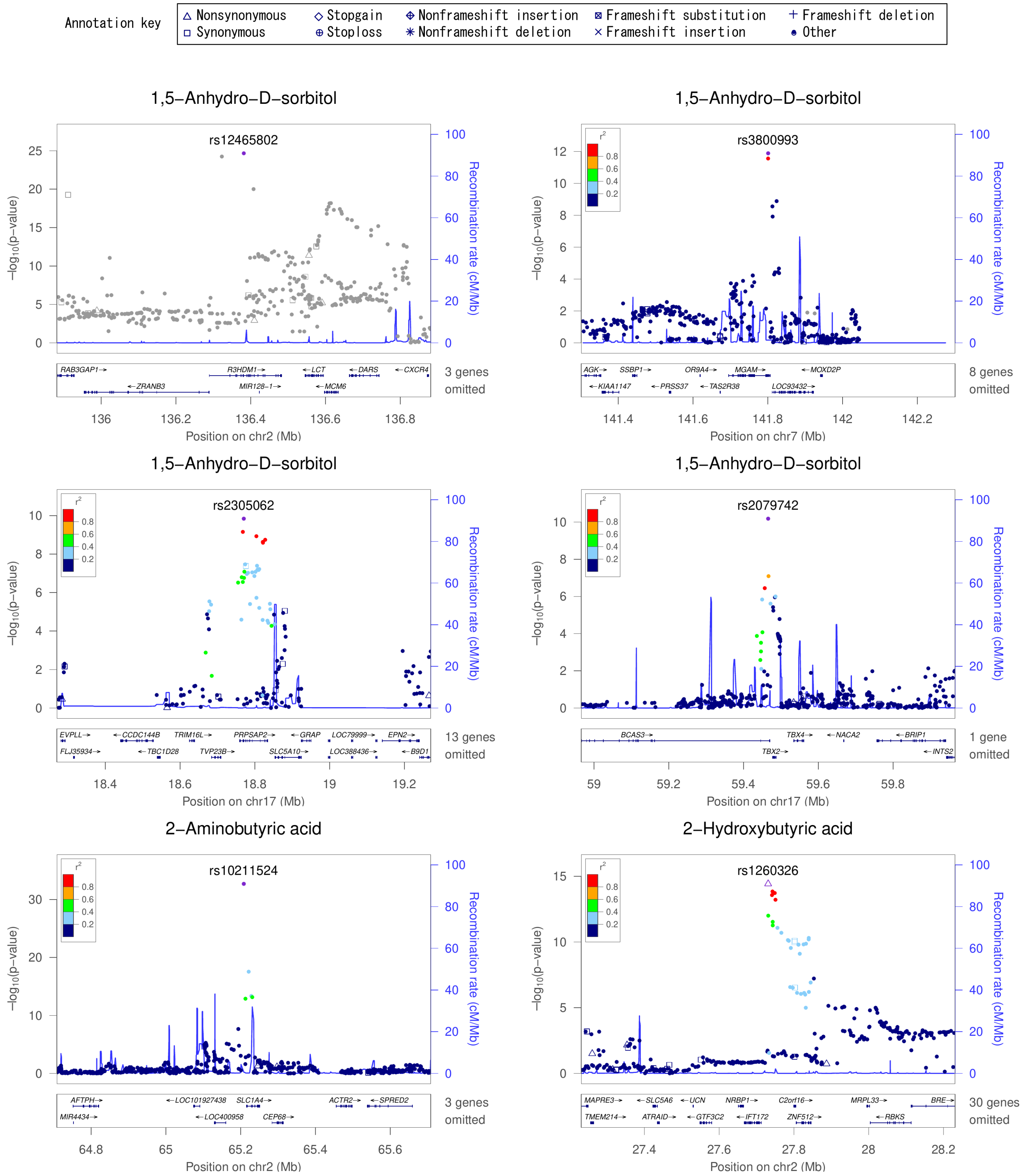

Figure S35. The region plots of identified loci in the multi-population meta-analysis, Related to Table 1

The calculated r^2 with the lead SNP in the East Asian (EAS) population (1KG Phase3) are shown in color. The gray plots indicate the lead SNP or the corresponding SNP was a monomorphic variant.

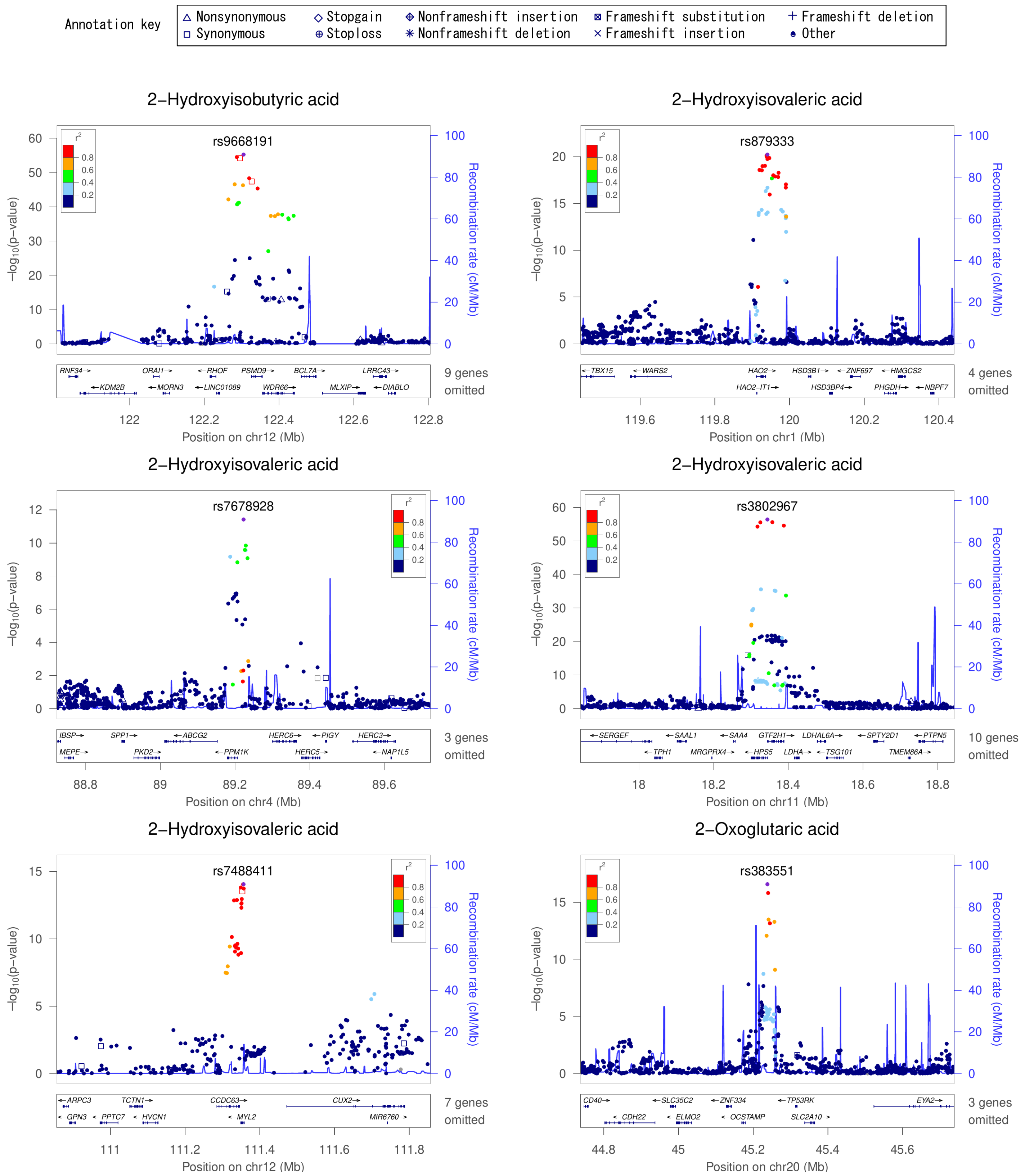

Figure S36. The region plots of identified loci in the multi-population meta-analysis, Related to Table 1

The calculated r^2 with the lead SNP in the East Asian (EAS) population (1KG Phase3) are shown in color. The gray plots indicate the lead SNP or the corresponding SNP was a monomorphic variant.

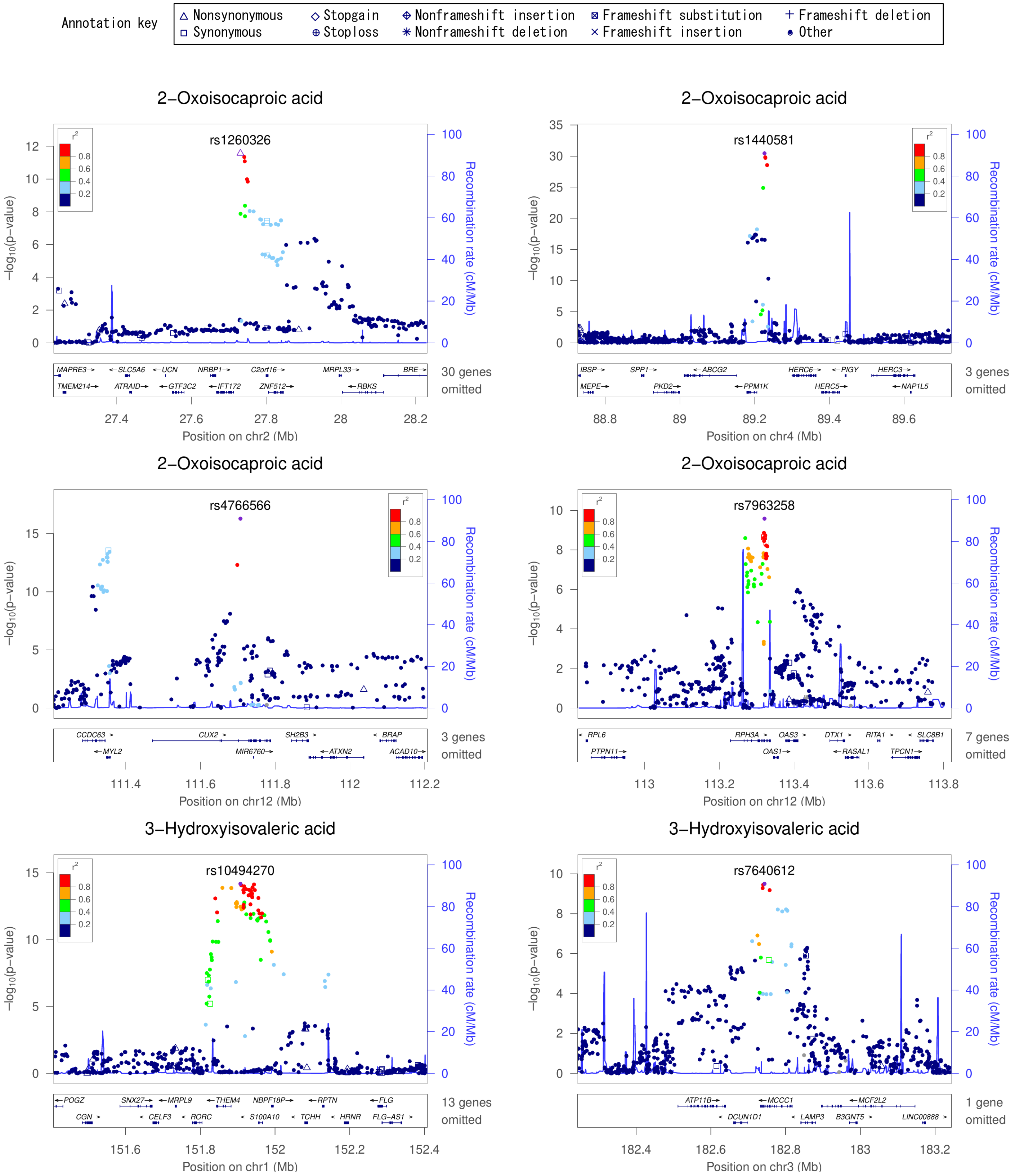

Figure S37. The region plots of identified loci in the multi-population meta-analysis, Related to Table 1

The calculated r^2 with the lead SNP in the East Asian (EAS) population (1KG Phase3) are shown in color. The gray plots indicate the lead SNP or the corresponding SNP was a monomorphic variant.

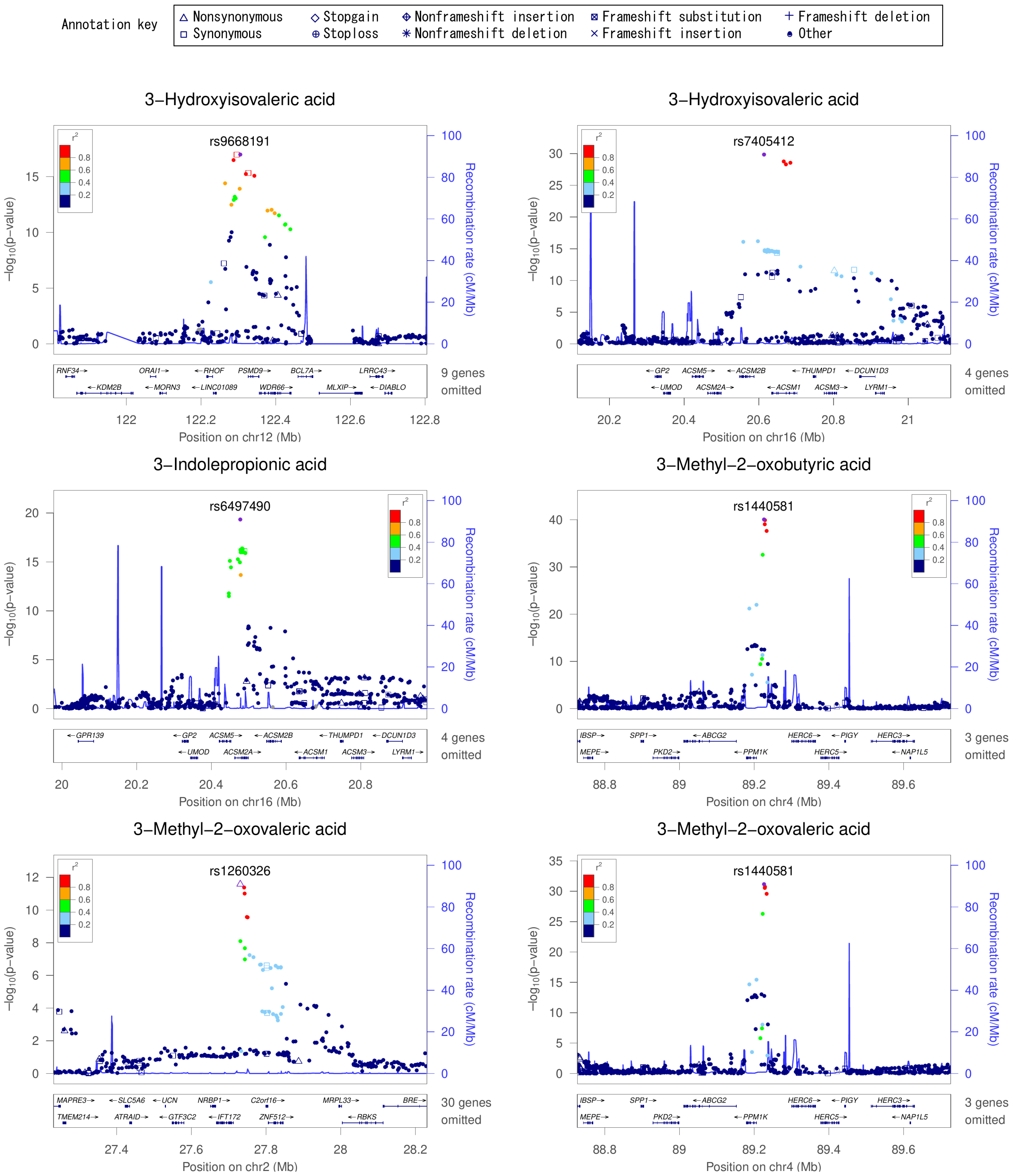

Figure S38. The region plots of identified loci in the multi-population meta-analysis, Related to Table 1

The calculated r^2 with the lead SNP in the East Asian (EAS) population (1KG Phase3) are shown in color. The gray plots indicate the lead SNP or the corresponding SNP was a monomorphic variant.

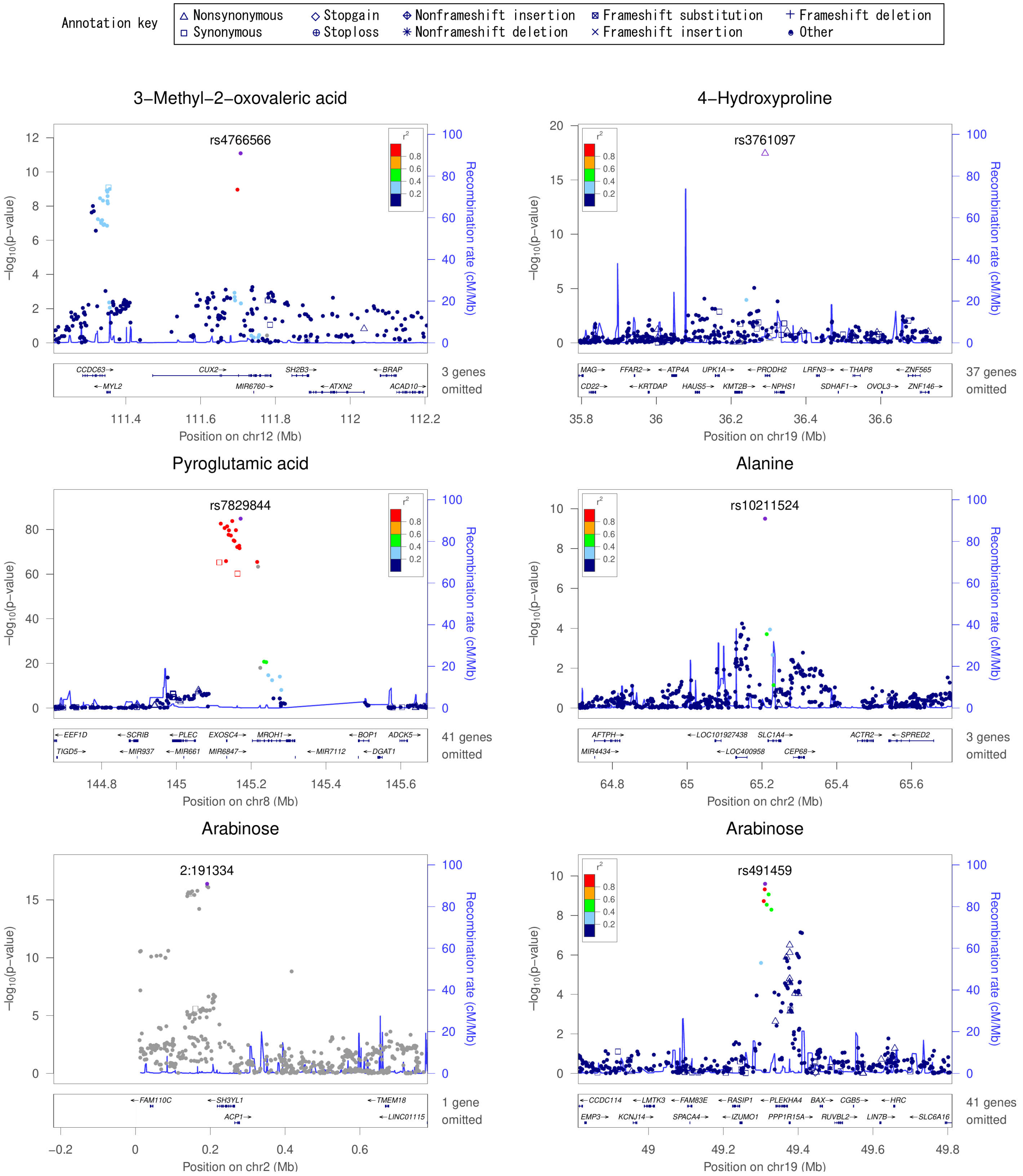

Figure S39. The region plots of identified loci in the multi-population meta-analysis, Related to Table 1

The calculated r^2 with the lead SNP in the East Asian (EAS) population (1KG Phase3) are shown in color. The gray plots indicate the lead SNP or the corresponding SNP was a monomorphic variant.

Annotation key: △ Nonsynonymous; □ Synonymous; ◇ Stopgain; ⊕ Stoploss; ◈ Nonframeshift insertion; ✳ Nonframeshift deletion; ⊠ Frameshift substitution; × Frameshift insertion; + Frameshift deletion; ● Other

Asparagine

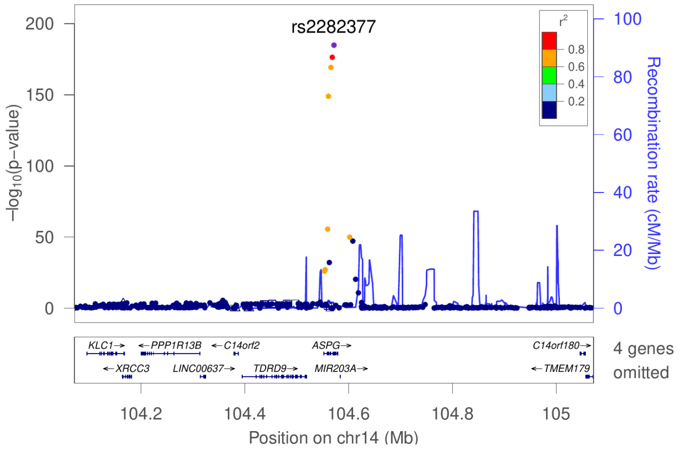

Aspartic acid

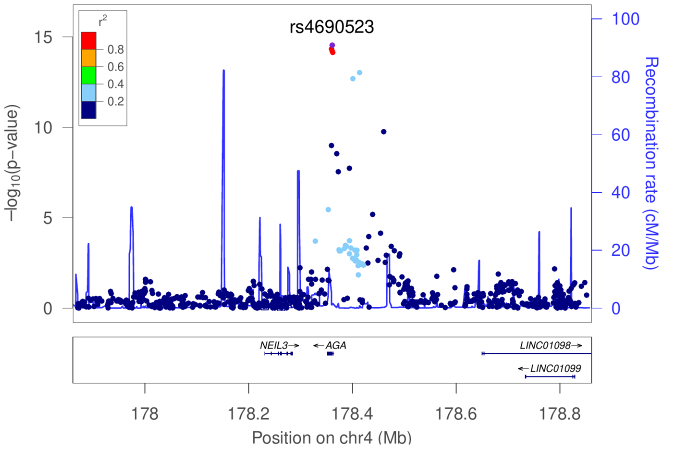

Citric acid

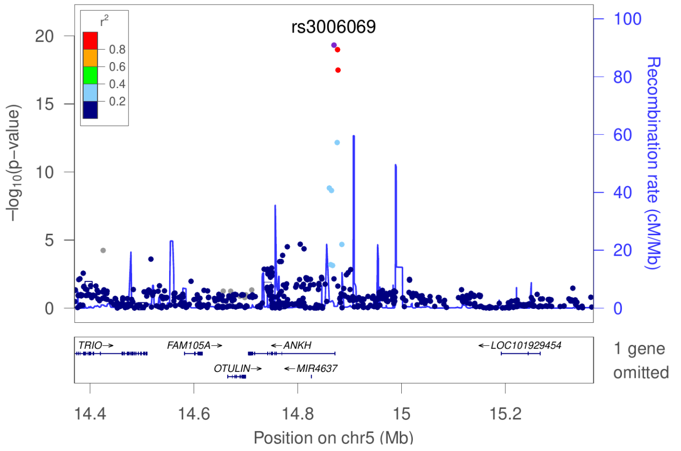

Citric acid

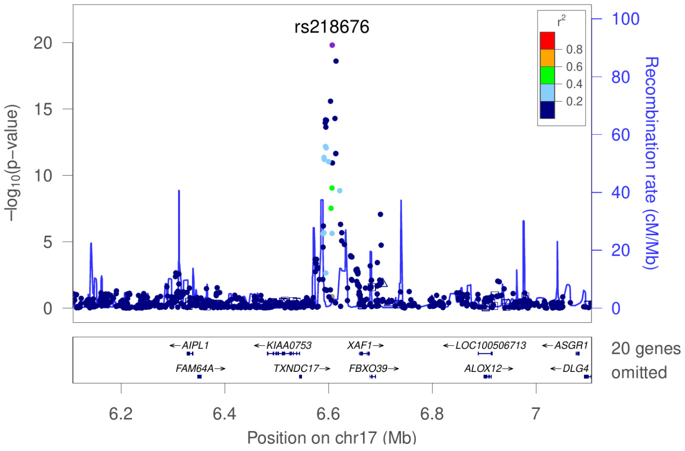

Citrulline

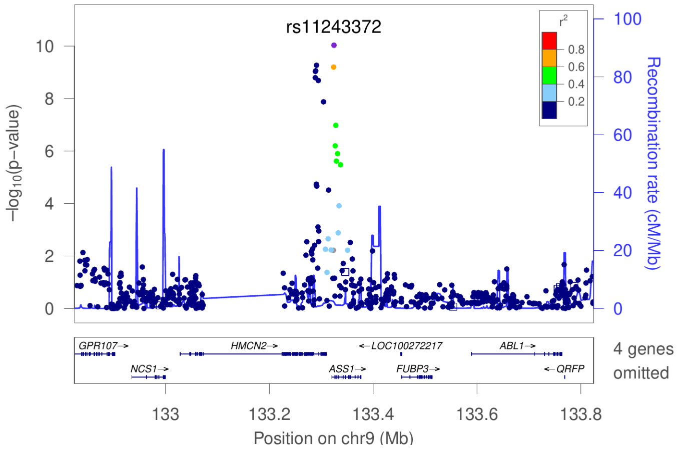

Creatinine

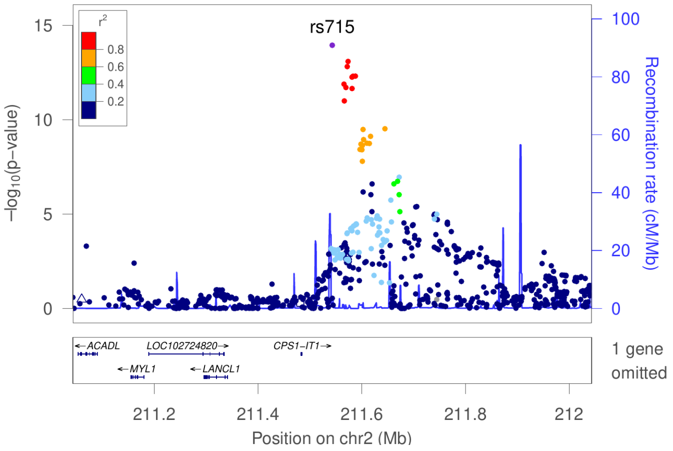

Figure S40. The region plots of identified loci in the multi-population meta-analysis, Related to Table 1

The calculated r^2 with the lead SNP in the East Asian (EAS) population (1KG Phase3) are shown in color. The gray plots indicate the lead SNP or the corresponding SNP was a monomorphic variant.

Annotation key					
	△ Nonsynonymous	◇ Stopgain	Nonframeshift insertion	⊠ Frameshift substitution	+ Frameshift deletion
	□ Synonymous	⊕ Stoploss	✳ Nonframeshift deletion	× Frameshift insertion	● Other

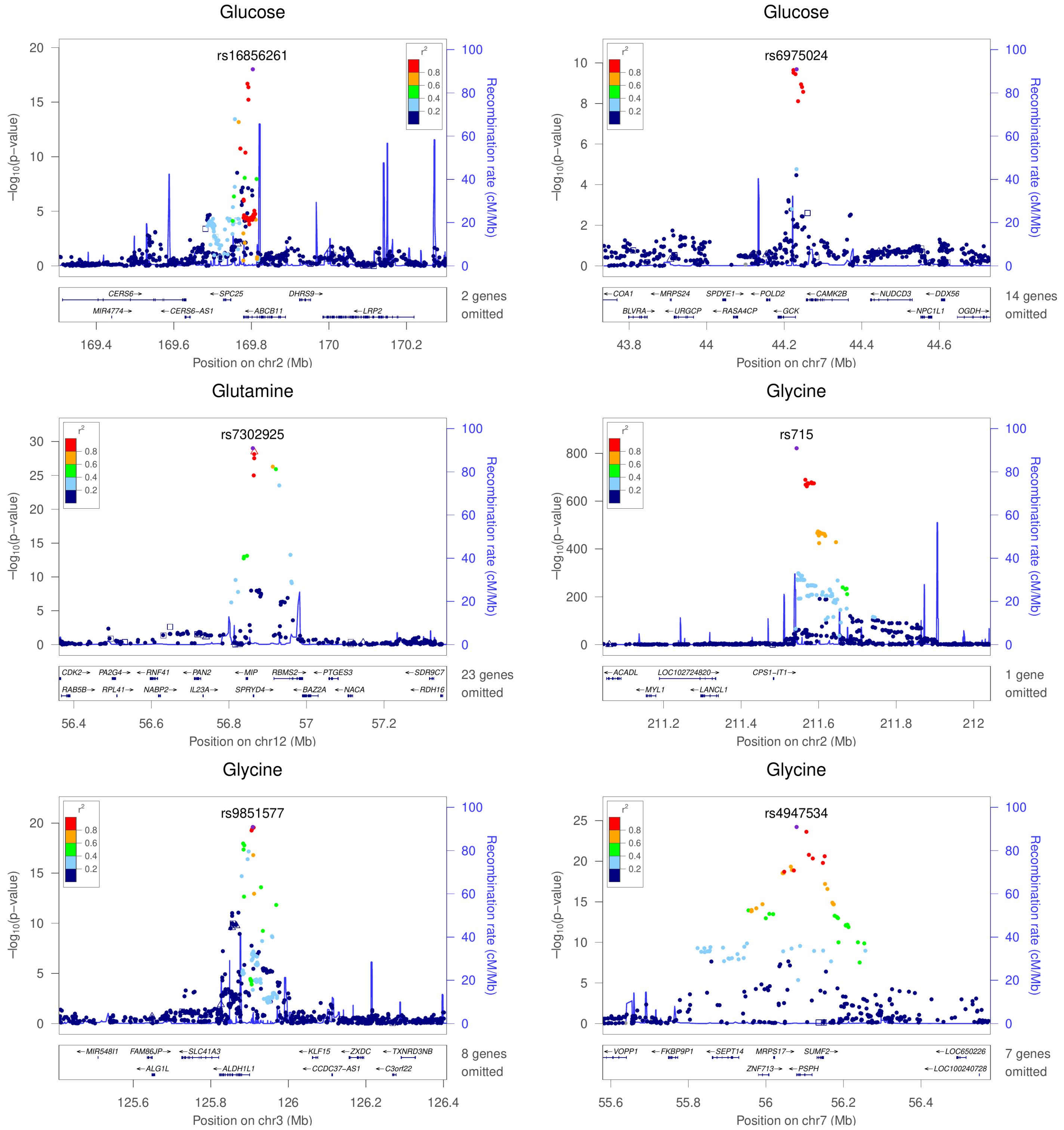

Figure S41. The region plots of identified loci in the multi-population meta-analysis, Related to Table 1

The calculated r^2 with the lead SNP in the East Asian (EAS) population (1KG Phase3) are shown in color. The gray plots indicate the lead SNP or the corresponding SNP was a monomorphic variant.

Annotation key: △ Nonsynonymous; □ Synonymous; ◇ Stopgain; ⊕ Stoploss; Nonframeshift insertion; ✳ Nonframeshift deletion; ⊠ Frameshift substitution; × Frameshift insertion; + Frameshift deletion; ● Other

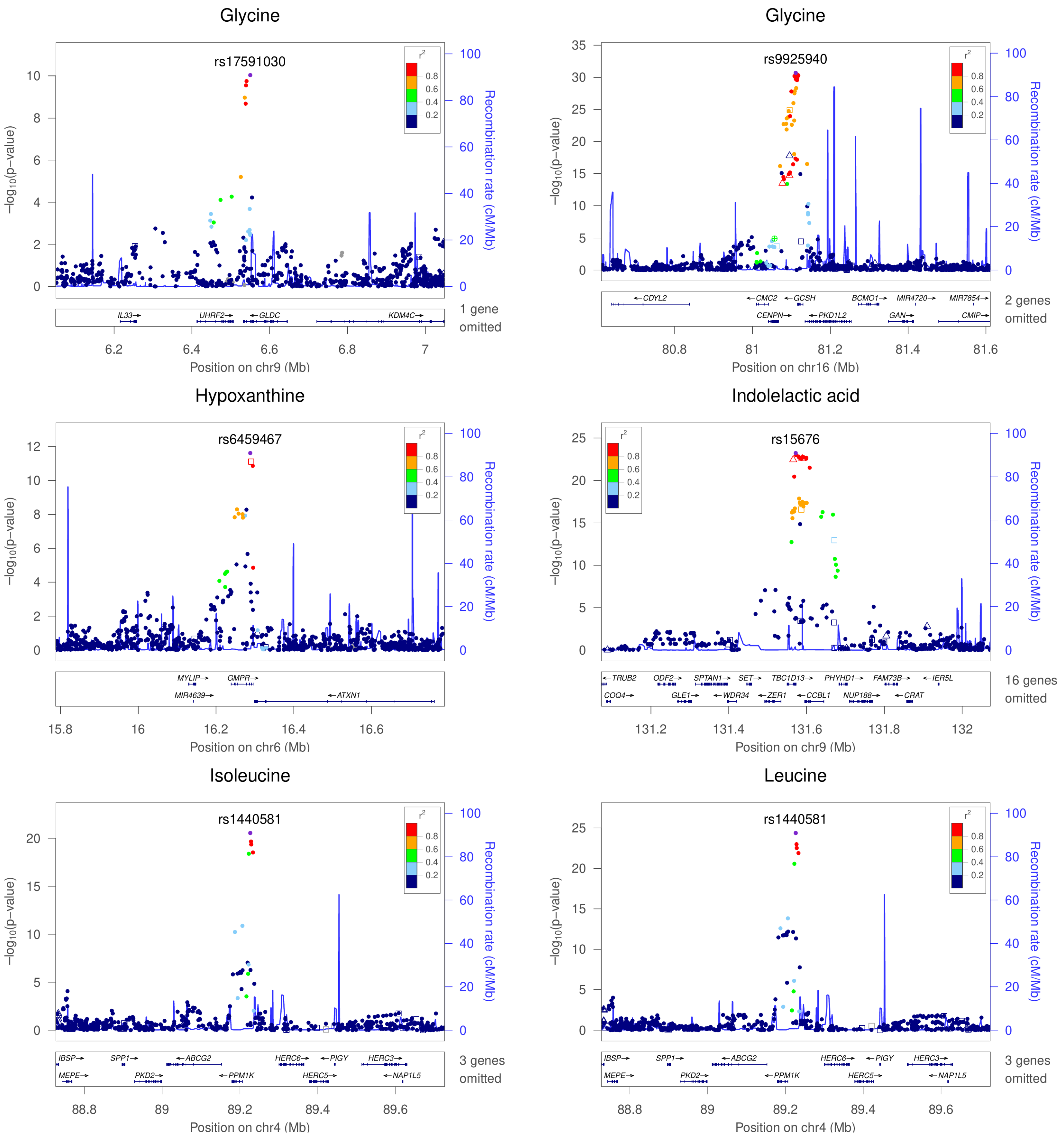

Figure S42. The region plots of identified loci in the multi-population meta-analysis, Related to Table 1

The calculated r^2 with the lead SNP in the East Asian (EAS) population (1KG Phase3) are shown in color. The gray plots indicate the lead SNP or the corresponding SNP was a monomorphic variant.

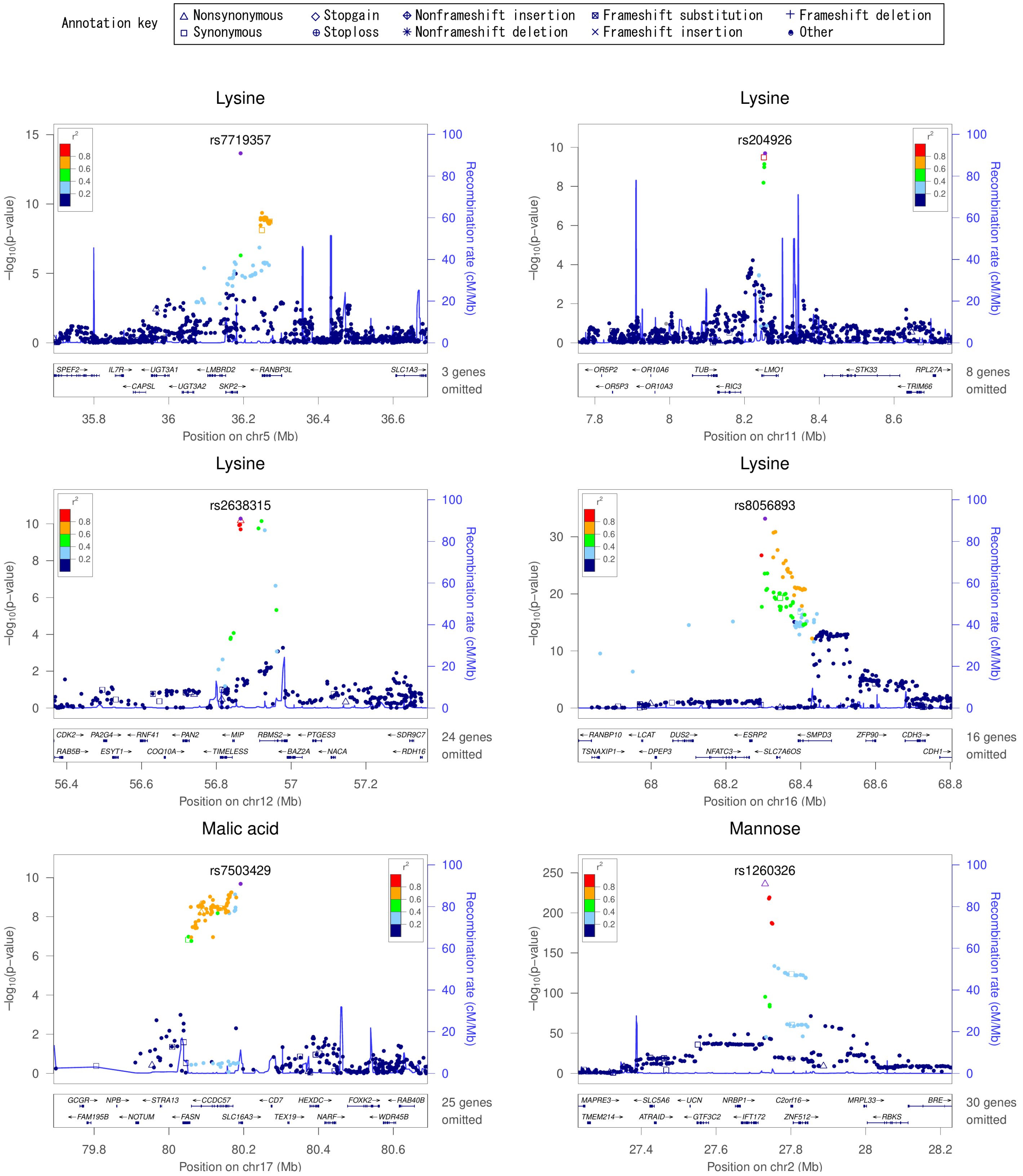

Figure S43. The region plots of identified loci in the multi-population meta-analysis, Related to Table 1

The calculated r^2 with the lead SNP in the East Asian (EAS) population (1KG Phase3) are shown in color. The gray plots indicate the lead SNP or the corresponding SNP was a monomorphic variant.

Annotation key: △ Nonsynonymous ◇ Stopgain ⊕ Nonframeshift insertion ⊠ Frameshift substitution + Frameshift deletion □ Synonymous ⊕ Stoploss ✳ Nonframeshift deletion × Frameshift insertion ● Other

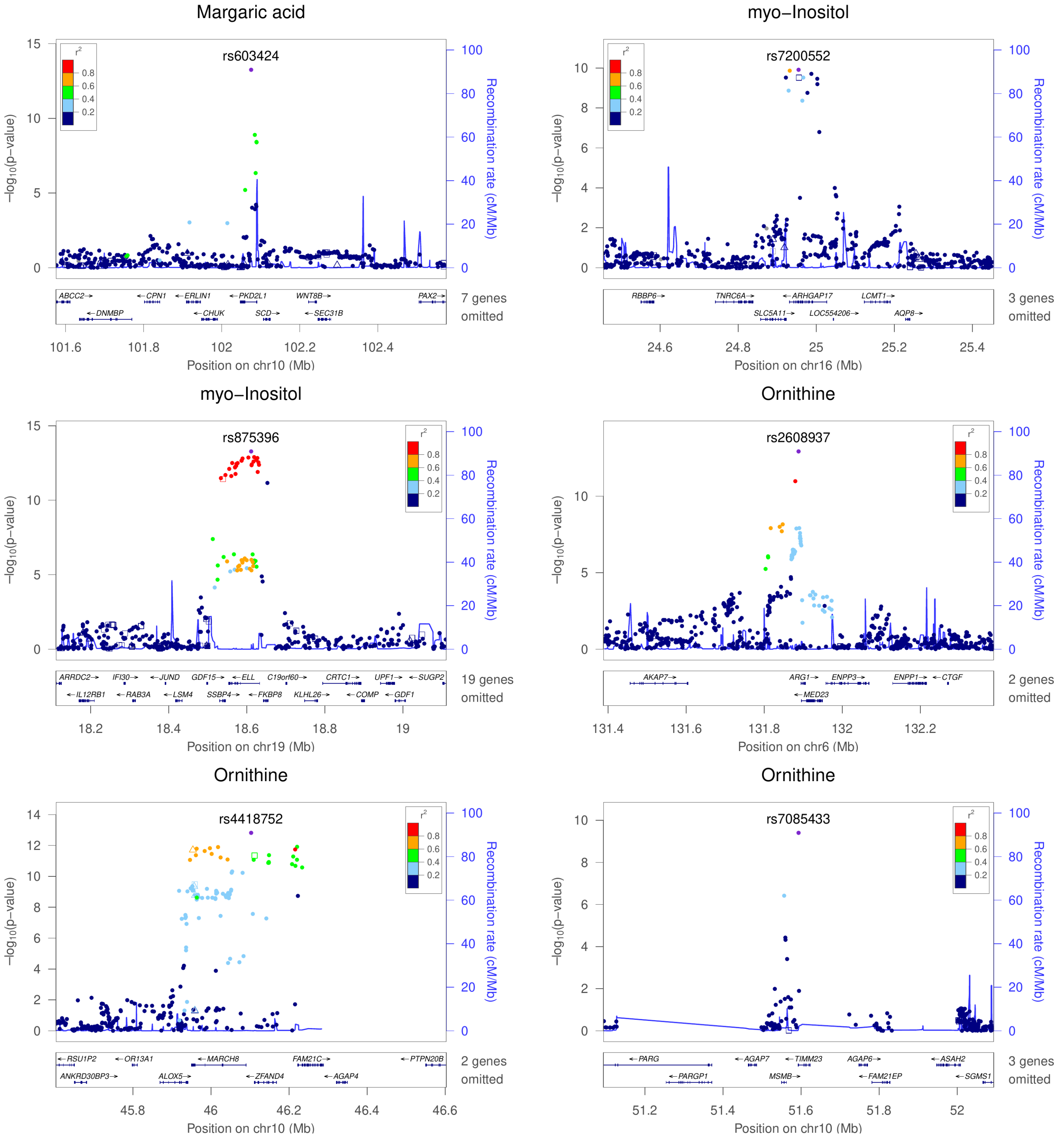

Figure S44. The region plots of identified loci in the multi-population meta-analysis, Related to Table 1

The calculated r^2 with the lead SNP in the East Asian (EAS) population (1KG Phase3) are shown in color. The gray plots indicate the lead SNP or the corresponding SNP was a monomorphic variant.

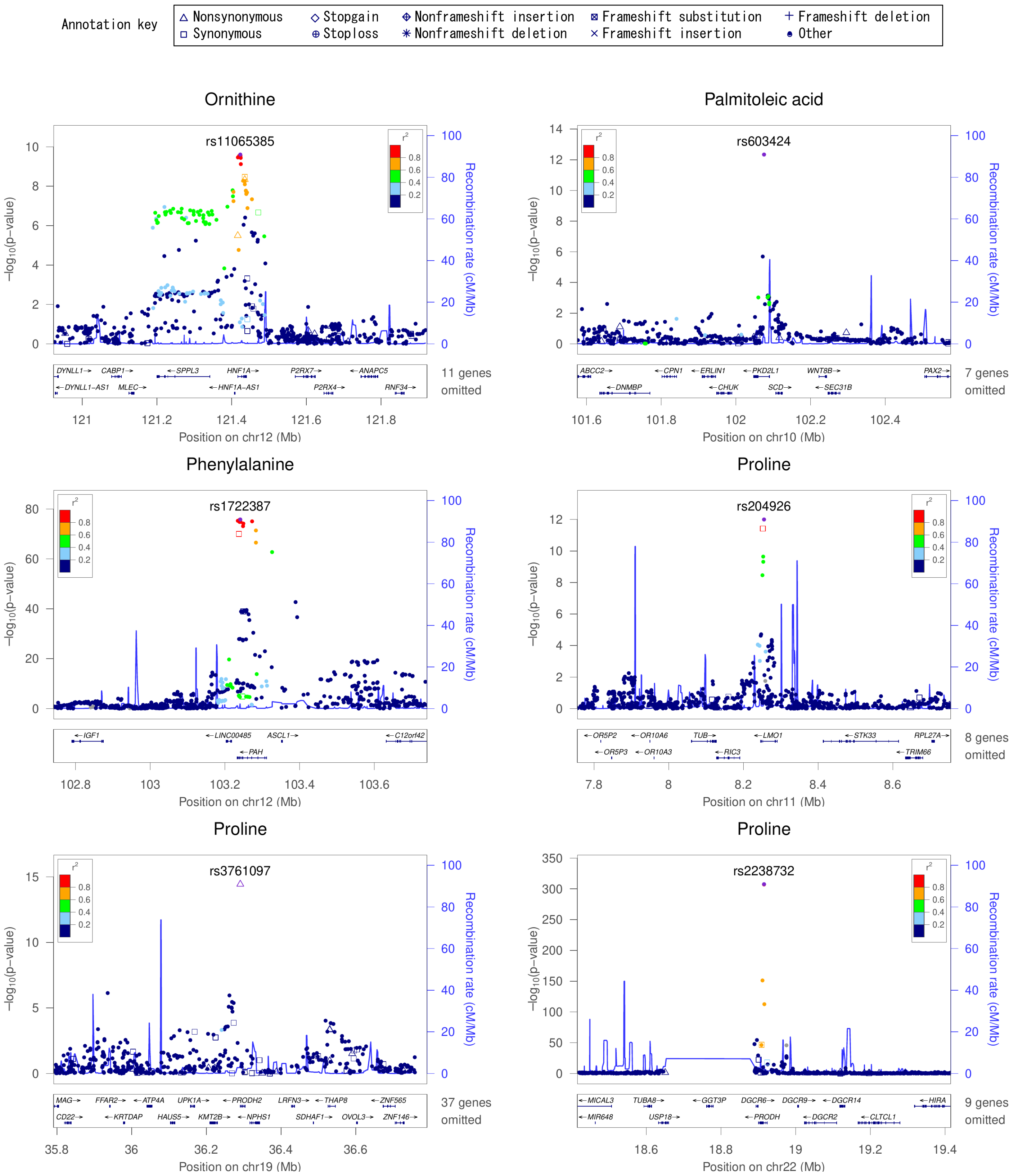

Figure S45. The region plots of identified loci in the multi-population meta-analysis, Related to Table 1

The calculated r^2 with the lead SNP in the East Asian (EAS) population (1KG Phase3) are shown in color. The gray plots indicate the lead SNP or the corresponding SNP was a monomorphic variant.

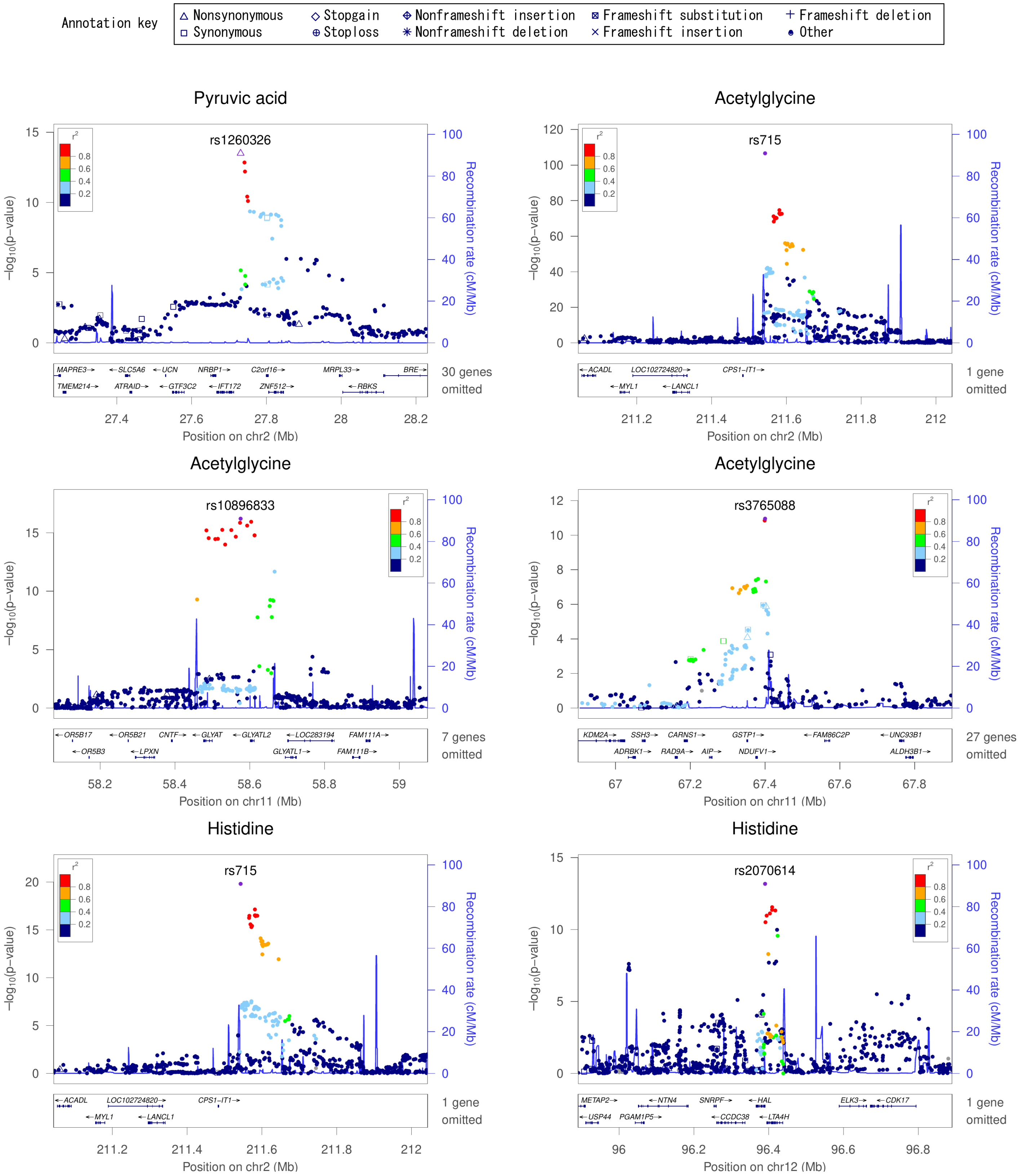

Figure S46. The region plots of identified loci in the multi-population meta-analysis, Related to Table 1

The calculated r^2 with the lead SNP in the East Asian (EAS) population (1KG Phase3) are shown in color. The gray plots indicate the lead SNP or the corresponding SNP was a monomorphic variant.

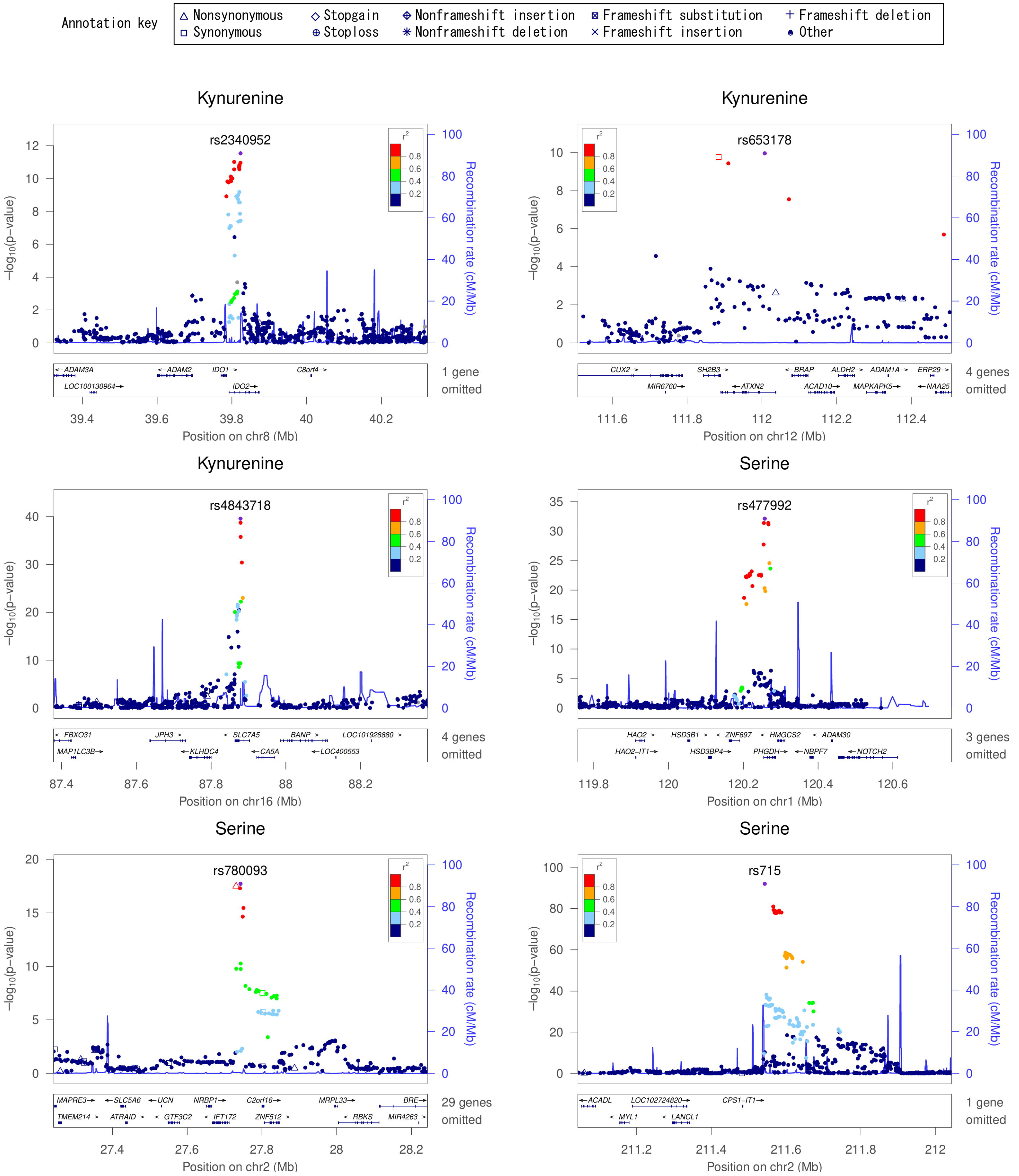

Figure S47. The region plots of identified loci in the multi-population meta-analysis, Related to Table 1

The calculated r^2 with the lead SNP in the East Asian (EAS) population (1KG Phase3) are shown in color. The gray plots indicate the lead SNP or the corresponding SNP was a monomorphic variant.

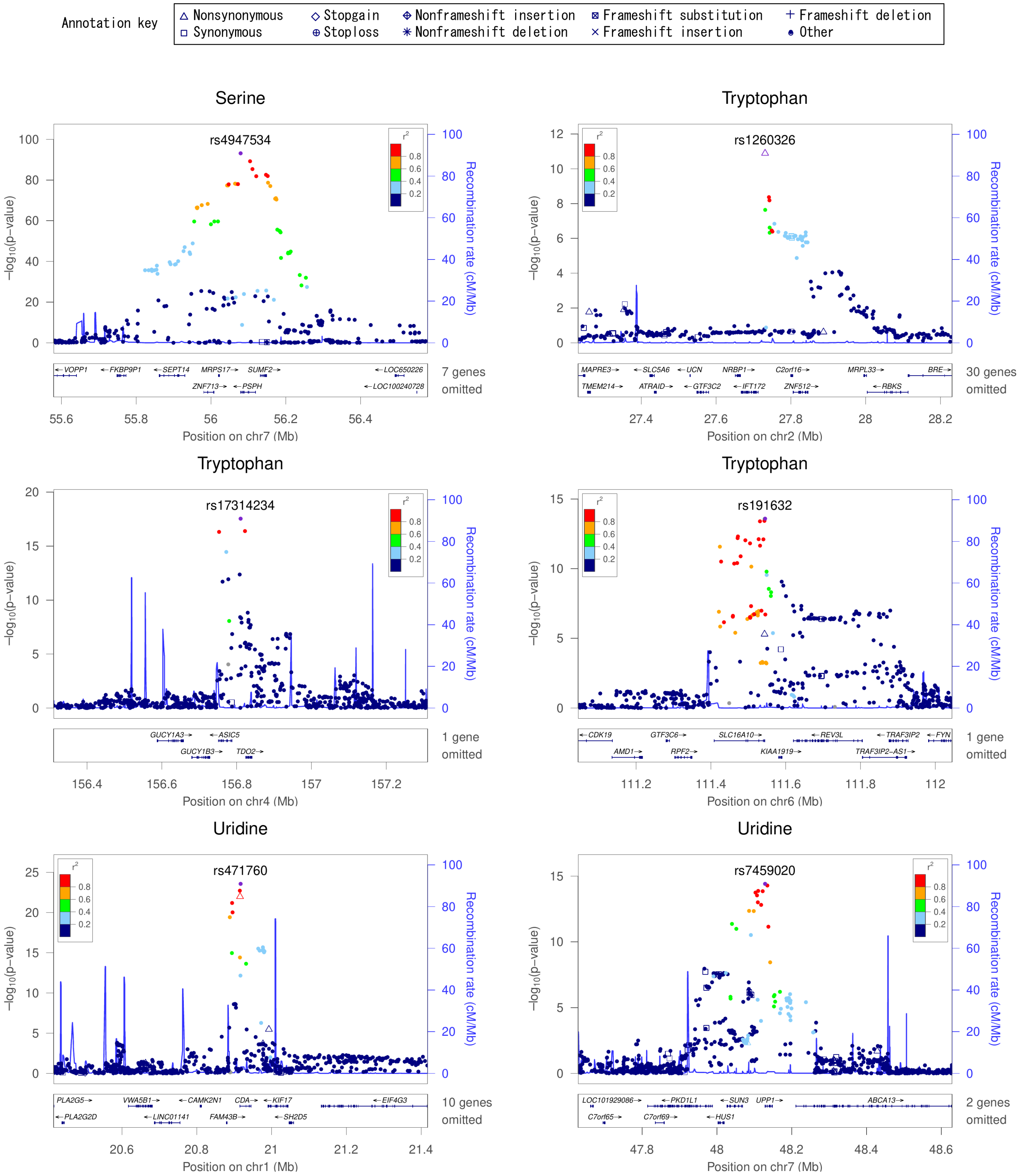

Figure S48. The region plots of identified loci in the multi-population meta-analysis, Related to Table 1

The calculated r^2 with the lead SNP in the East Asian (EAS) population (1KG Phase3) are shown in color. The gray plots indicate the lead SNP or the corresponding SNP was a monomorphic variant.

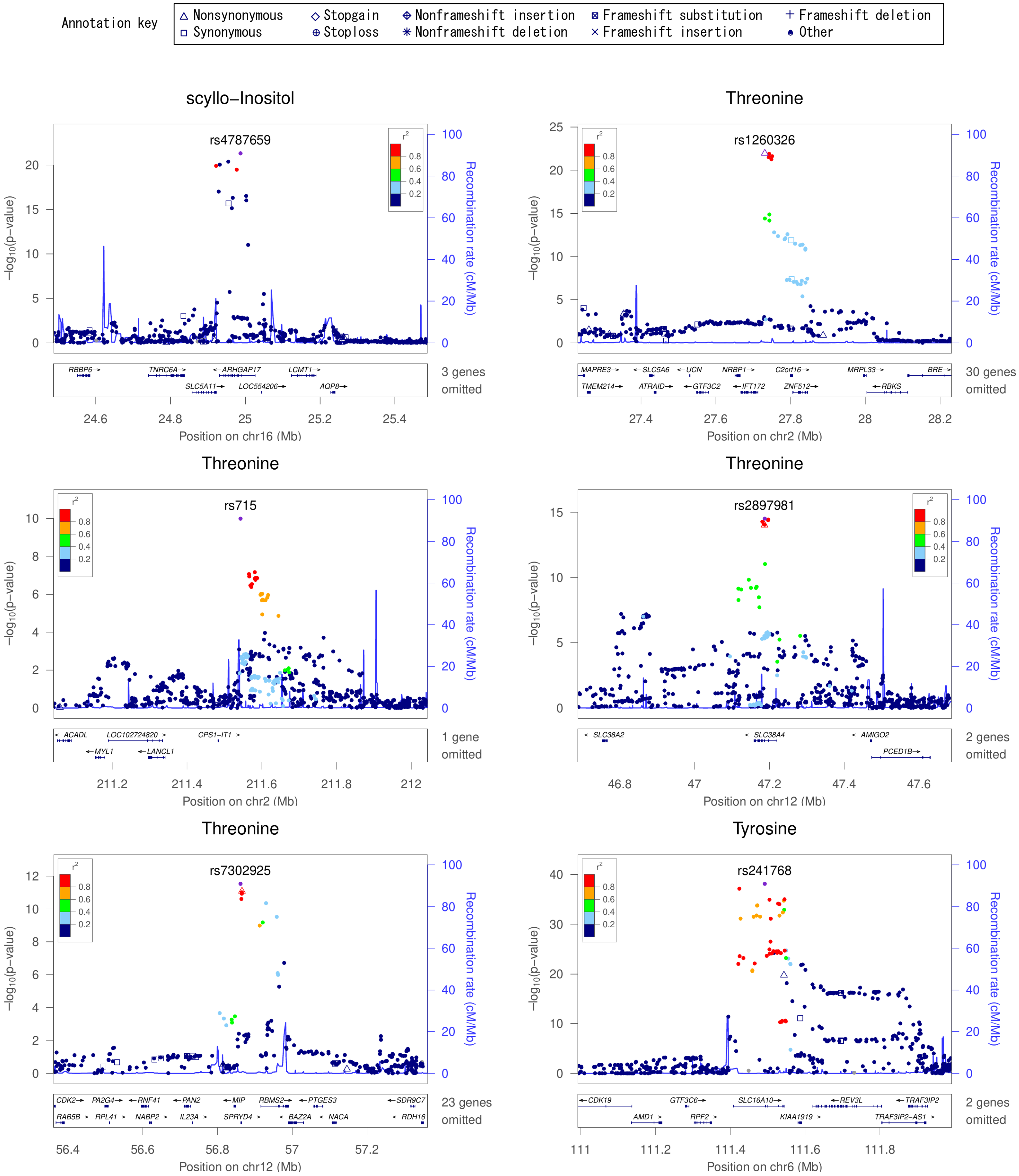

Figure S49. The region plots of identified loci in the multi-population meta-analysis, Related to Table 1

The calculated r^2 with the lead SNP in the East Asian (EAS) population (1KG Phase3) are shown in color. The gray plots indicate the lead SNP or the corresponding SNP was a monomorphic variant.

Annotation key: △ Nonsynonymous, □ Synonymous, ◇ Stopgain, ⊕ Stoploss, Nonframeshift insertion, ✳ Nonframeshift deletion, ⊠ Frameshift substitution, × Frameshift insertion, + Frameshift deletion, ● Other

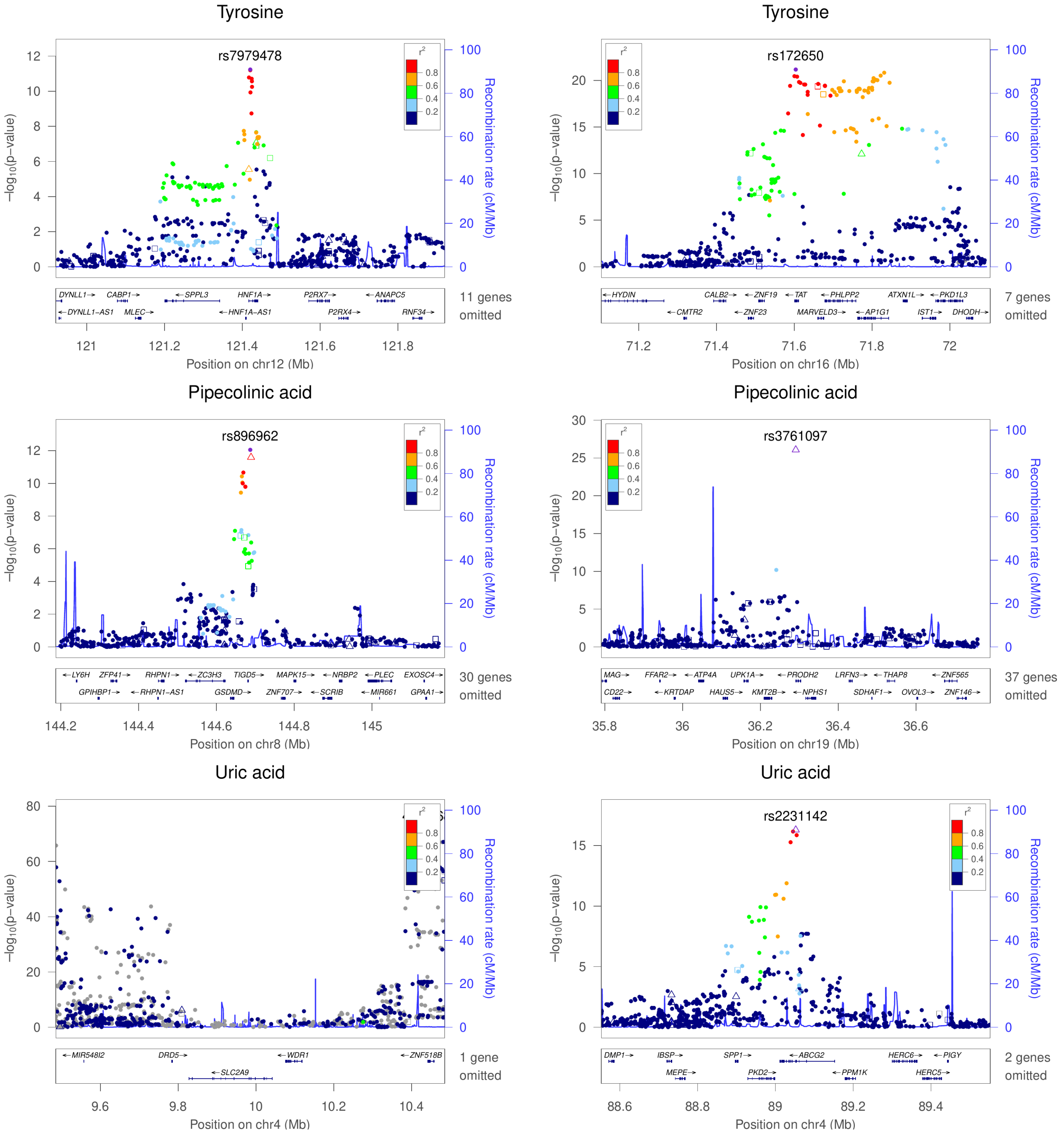

Figure S50. The region plots of identified loci in the multi-population meta-analysis, Related to Table 1

The calculated r^2 with the lead SNP in the East Asian (EAS) population (1KG Phase3) are shown in color. The gray plots indicate the lead SNP or the corresponding SNP was a monomorphic variant.

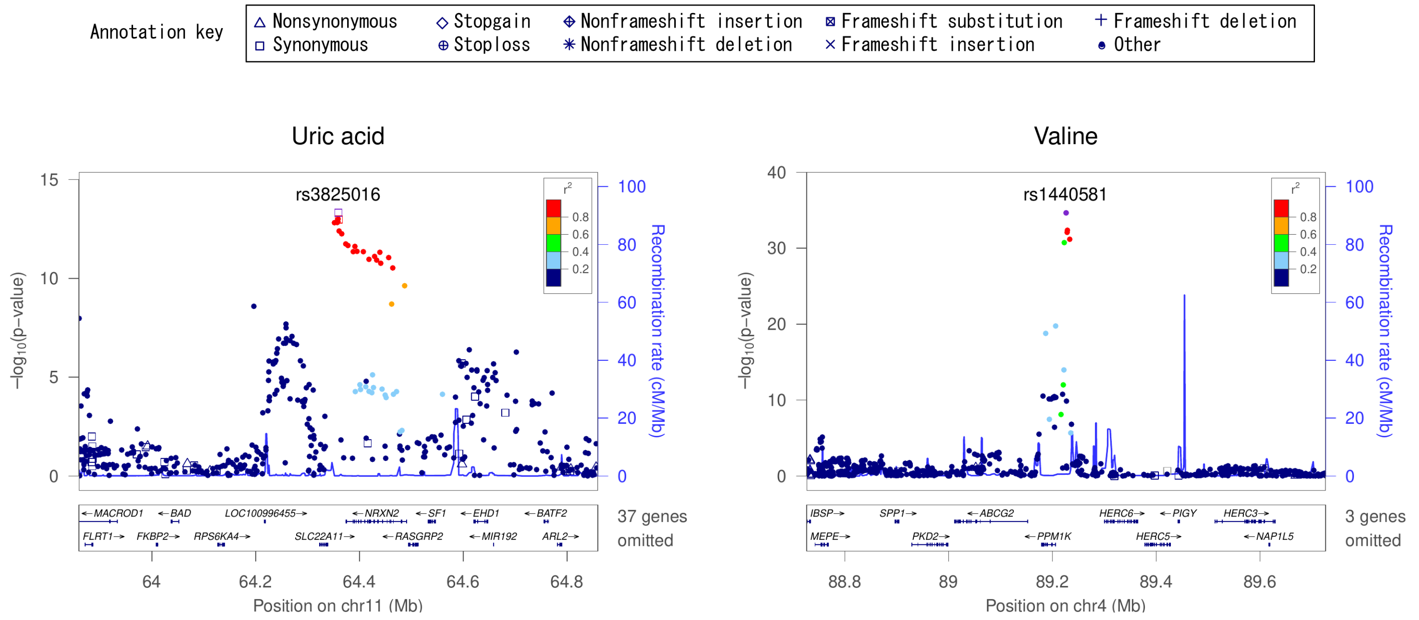

Figure S51. Selection signature and polygenicity for each metabolite estimated by BayesS model using genome-wide SNPs, Related to Figure 5

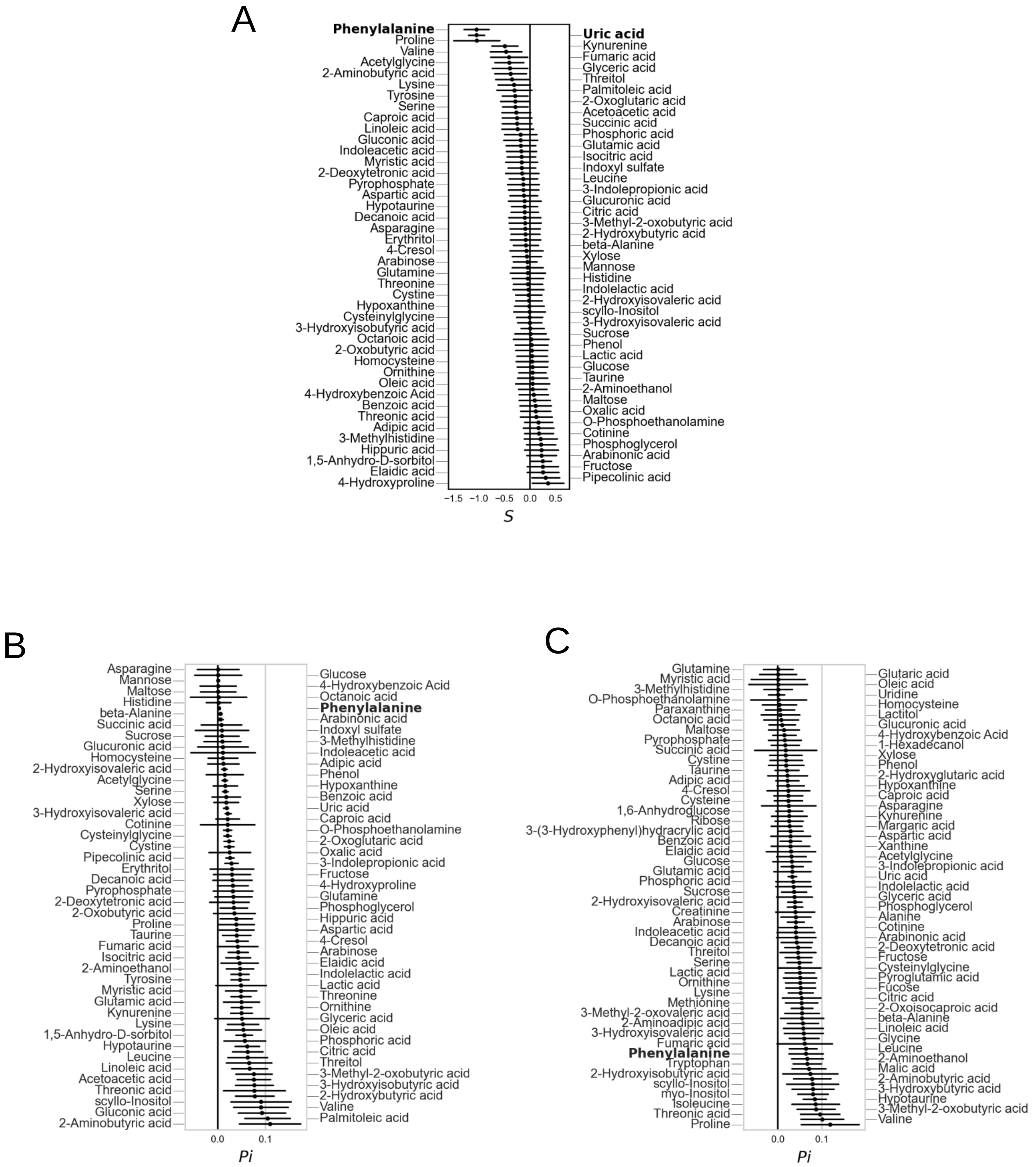

A. Selection signature for each metabolite estimated by BayesS model using genome-wide SNPs. The metabolite in bold font showed significant selection signature.
B. Polygenicity for each metabolite estimated by BayesS model using genome-wide SNPs.
C. Polygenicity for each metabolite estimated by BayesS model using genome-wide SNPs excluding the corresponding metabolites’ QTL and regions with pleiotrophic effect on multiple metabolites.

Figure S52. Validation of metabolite measurement, Related to STAR Methods

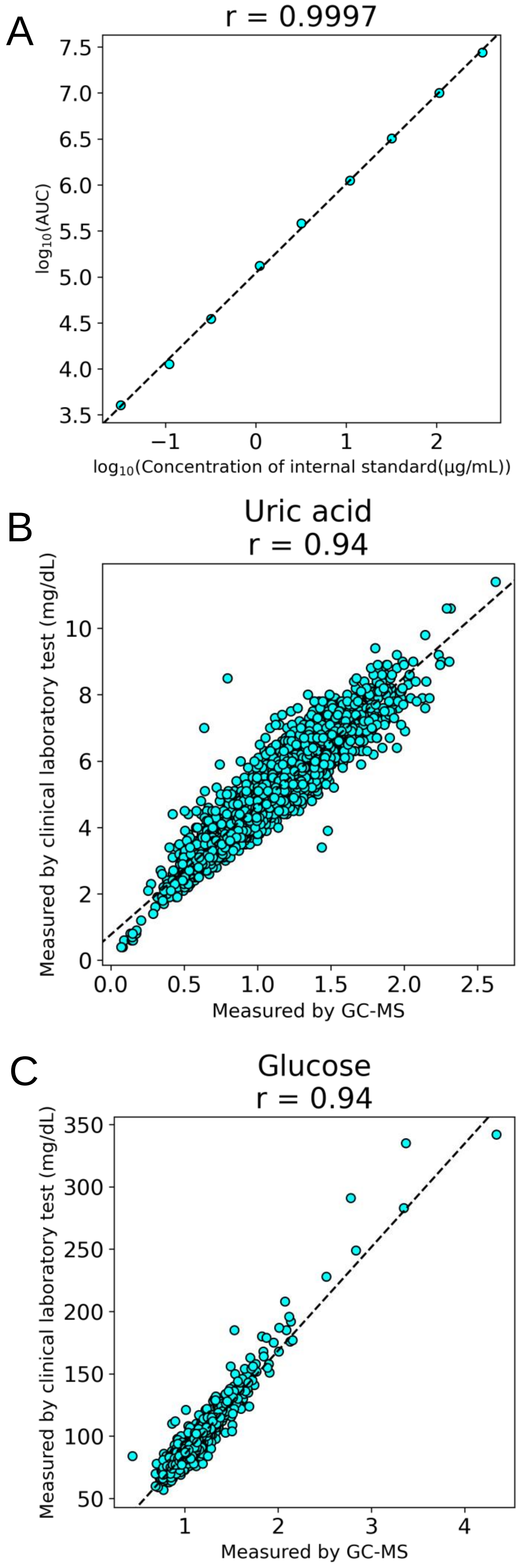

Figure S53. The PCA plots of metabolome data, Related to STAR Methods

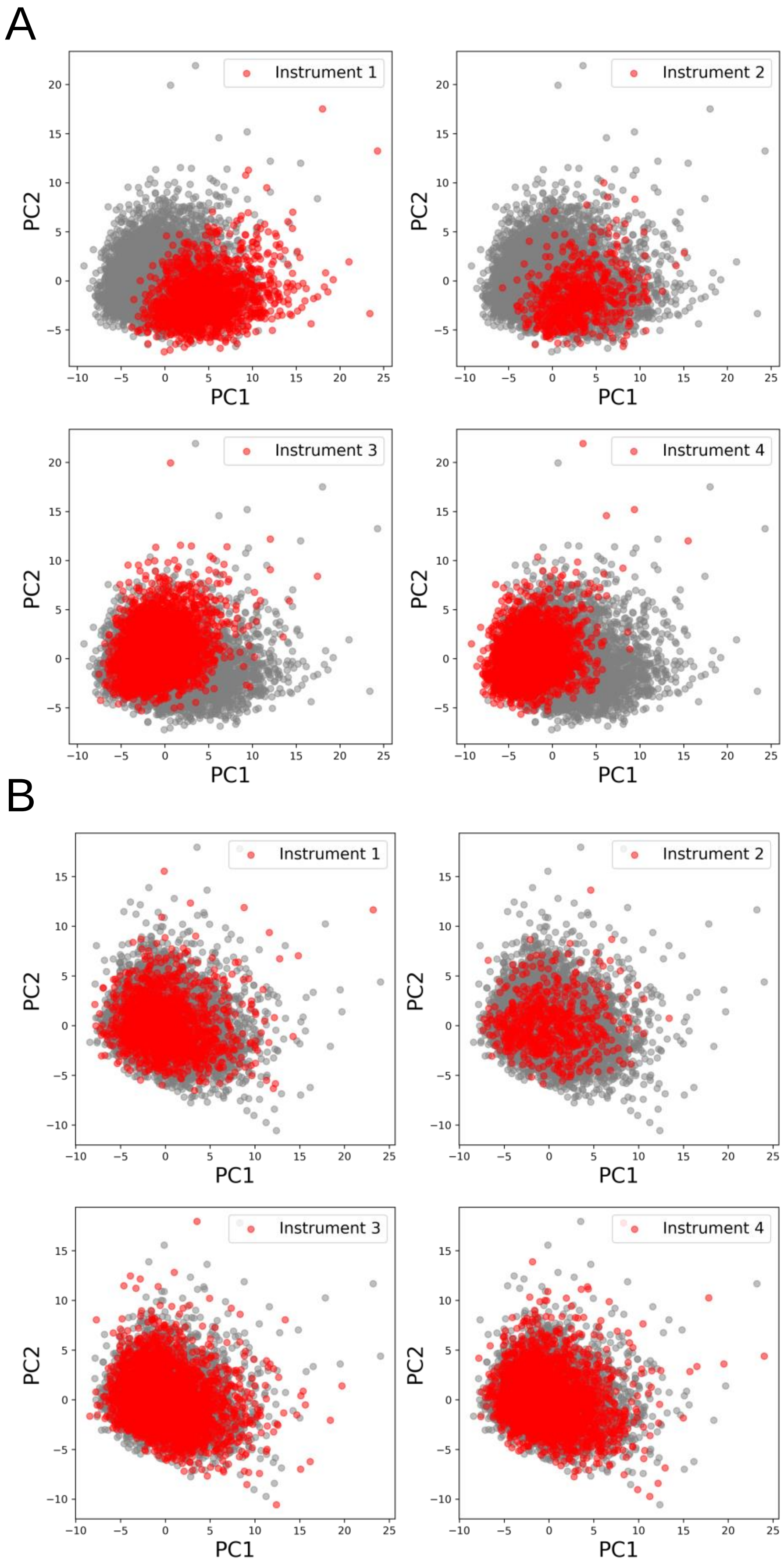

The PCA plots before (A) and after (B) normalization using 121 metabolite concentration data from 8,270 samples. For each instrument, the sample measured with it is indicated in red.

Figure S54. The flow of quality control of samples and SNPs, Related to STAR Methods

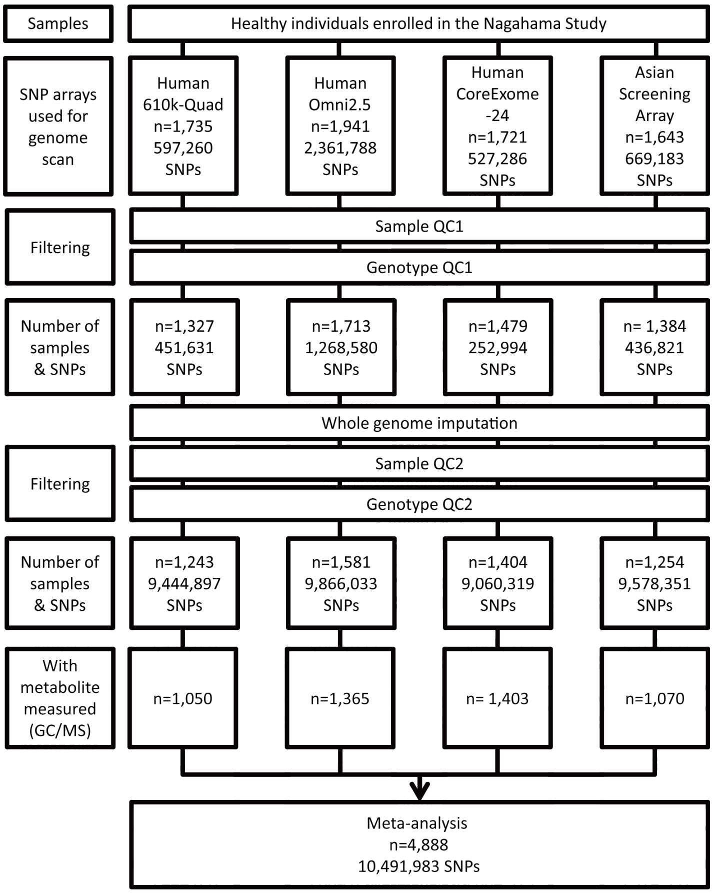

Sample QC 1 : Exclude samples showing one of the following criteria.

1. Having PI_HAT in PLINK software (ver1.9b, Purcell et al., 2007) greater than 0.1 with any other sample.
2. Outliers from the EAS population (1KG Phase 3) in the principal components of joint samples by the Nagahama and 1KG populations.
3. Success rate of SNPs is less than 95% in Human 610k-Quad (610k), Omni2.5, Asian Screening Array (ASA), less than 98% in Human CoreExome-24.

Genotype QC 1 : Exclude variants showing one of the following criteria

1. The MAF is less than 0.01 in 610k , ASA, less than 0.005 in Omni 2.5, HumanCoreExome-24
2. Hardy-Weinberg equilibrium (HWE) p-value is less than 1.0 $\times 10^{-6}$
3. Success rate of variant is less than 98%

Sample QC2 : Exclude samples showing the following criteria.

One of the top ten principal component scores of SNPs is out of range (–3 $\times$ IQR below the 25th percentile or 3 $\times$ IQR above the 75th percentile)

Genotype QC2 : We excluded variants showing one of the following criteria.

1. MAF is less than 0.005.
2. r^2 is less than 0.3.

Figure S55. The flow of quality control of studies and SNPs included in the summary of 40 metbolite GWAS, Related to STAR Methods

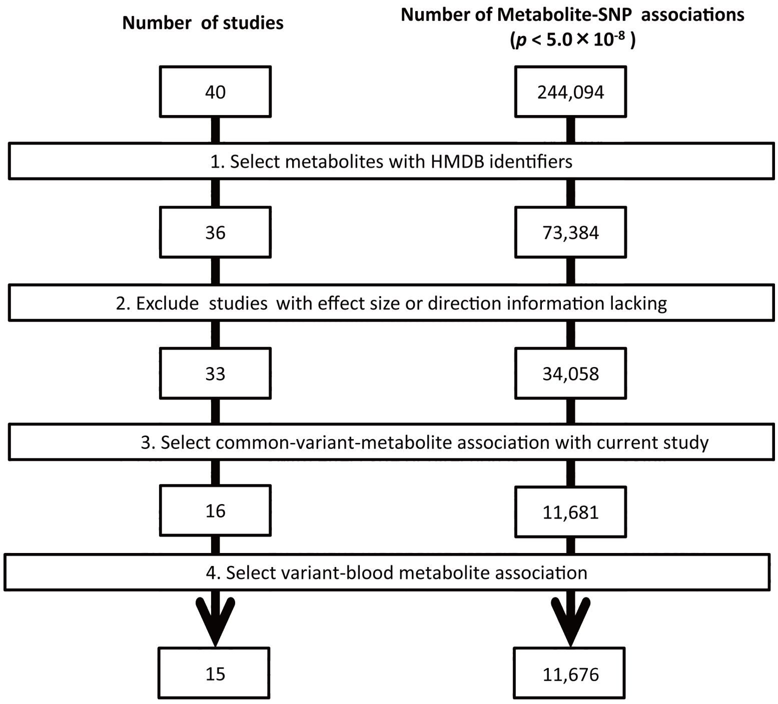

Figure S56. The flow of quality control of studies and SNPs included in the GWAS Catalog, Related to STAR Methods

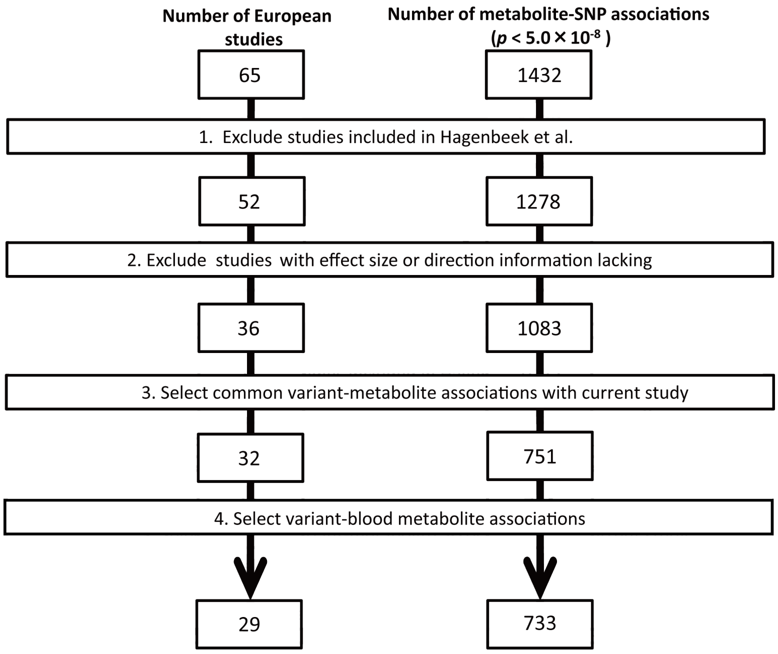

References for Table 1.

1. Schlosser, P., Li, Y., Sekula, P., Raffler, J., Grundner-Culemann, F., Pietzner, M., Cheng, Y., Wuttke, M., Steinbrenner, I., Schultheiss, U.T., et al. (2020). Genetic studies of urinary metabolites illuminate mechanisms of detoxification and excretion in humans. Nature genetics 52, 167-176. 10.1038/s41588-019-0567-8.
2. Sinnott-Armstrong, N., Tanigawa, Y., Amar, D., Mars, N., Benner, C., Aguirre, M., Venkataraman, G.R., Wainberg, M., Ollila, H.M., Kiiskinen, T., et al. (2021). Genetics of 35 blood and urine biomarkers in the UK Biobank. Nature genetics 53, 185-194. 10.1038/s41588-020-00757-z.
3. Yin, X., Chan, L.S., Bose, D., Jackson, A.U., VandeHaar, P., Locke, A.E., Fuchsberger, C., Stringham, H.M., Welch, R., Yu, K., et al. (2022). Genome-wide association studies of metabolites in Finnish men identify disease-relevant loci. Nature communications 13, 1644. 10.1038/s41467-022-29143-5.
4. Feofanova, E.V., Chen, H., Dai, Y., Jia, P., Grove, M.L., Morrison, A.C., Qi, Q., Daviglus, M., Cai, J., North, K.E., et al. (2020). A Genome-wide Association Study Discovers 46 Loci of the Human Metabolome in the Hispanic Community Health Study/Study of Latinos. American journal of human genetics 107, 849-863. 10.1016/j.ajhg.2020.09.003.
5. Shin, S.Y., Fauman, E.B., Petersen, A.K., Krumsiek, J., Santos, R., Huang, J., Arnold, M., Erte, I., Forgetta, V., Yang, T.P., et al. (2014). An atlas of genetic influences on human blood metabolites. Nature genetics 46, 543-550. 10.1038/ng.2982.
6. Long, T., Hicks, M., Yu, H.C., Biggs, W.H., Kirkness, E.F., Menni, C., Zierer, J., Small, K.S., Mangino, M., Messier, H., et al. (2017). Whole-genome sequencing identifies common-to-rare variants associated with human blood metabolites. Nature genetics 49, 568-578. 10.1038/ng.3809.
7. Korostishevsky, M., Steves, C.J., Malkin, I., Spector, T., Williams, F.M., and Livshits, G. (2016). Genomics and metabolomics of muscular mass in a community-based sample of UK females. European journal of human genetics : EJHG 24, 277-283. 10.1038/ejhg.2015.85.
8. Krumsiek, J., Suhre, K., Evans, A.M., Mitchell, M.W., Mohney, R.P., Milburn, M.V., Wagele, B., Romisch-Margl, W., Illig, T., Adamski, J., et al. (2012). Mining the unknown: a systems approach to metabolite identification combining genetic and metabolic information. PLoS Genet 8, e1003005. 10.1371/journal.pgen.1003005.
9. Yet, I., Menni, C., Shin, S.Y., Mangino, M., Soranzo, N., Adamski, J., Suhre, K., Spector, T.D., Kastenmuller, G., and Bell, J.T. (2016). Genetic Influences on Metabolite Levels: A Comparison across Metabolomic Platforms. PloS one 11, e0153672. 10.1371/journal.pone.0153672.
10. Feofanova, E.V., Chen, H., Dai, Y., Jia, P., Grove, M.L., Morrison, A.C., Qi, Q., Daviglus, M., Cai, J., North, K.E., et al. (2020). A Genome-wide Association Study Discovers 46 Loci of the Human Metabolome in the Hispanic Community Health Study/Study of Latinos. American journal of human genetics 107, 849-863. 10.1016/j.ajhg.2020.09.003.
11. Sakaue, S., Kanai, M., Tanigawa, Y., Karjalainen, J., Kurki, M., Koshiba, S., Narita, A., Konuma, T., Yamamoto, K., Akiyama, M., et al. (2021). A cross-population atlas of genetic associations for 220 human phenotypes. Nature genetics 53, 1415-1424. 10.1038/s41588-021-00931-x.
12. Lotta, L.A., Pietzner, M., Stewart, I.D., Wittemans, L.B.L., Li, C., Bonelli, R., Raffler, J., Biggs, E.K., Oliver-Williams, C., Auyeung, V.P.W., et al. (2021). A cross-platform approach identifies genetic regulators of human metabolism and health. Nature genetics 53, 54-64. 10.1038/s41588-020-00751-5.
13. Koshiba, S., Motoike, I.N., Saigusa, D., Inoue, J., Aoki, Y., Tadaka, S., Shirota, M., Katsuoka, F., Tamiya,

G., Minegishi, N., et al. (2020). Identification of critical genetic variants associated with metabolic phenotypes of the Japanese population. Communications biology 3, 662. 10.1038/s42003-020-01383-5.
14. Li, Y., Sekula, P., Wuttke, M., Wahrheit, J., Hausknecht, B., Schultheiss, U.T., Gronwald, W., Schlosser, P., Tucci, S., Ekici, A.B., et al. (2018). Genome-Wide Association Studies of Metabolites in Patients with CKD Identify Multiple Loci and Illuminate Tubular Transport Mechanisms. J Am Soc Nephrol 29, 1513-1524. 10.1681/ASN.2017101099.
15. Bar, N., Korem, T., Weissbrod, O., Zeevi, D., Rothschild, D., Leviatan, S., Kosower, N., Lotan-Pompan, M., Weinberger, A., Le Roy, C.I., et al. (2020). A reference map of potential determinants for the human serum metabolome. Nature 588, 135-140. 10.1038/s41586-020-2896-2.
16. Teslovich, T.M., Kim, D.S., Yin, X., Stancakova, A., Jackson, A.U., Wielscher, M., Naj, A., Perry, J.R.B., Huyghe, J.R., Stringham, H.M., et al. (2018). Identification of seven novel loci associated with amino acid levels using single-variant and gene-based tests in 8545 Finnish men from the METSIM study. Human molecular genetics 27, 1664-1674. 10.1093/hmg/ddy067.
17. Chai, J.F., Raichur, S., Khor, I.W., Torta, F., Chew, W.S., Herr, D.R., Ching, J., Kovalik, J.P., Khoo, C.M., Wenk, M.R., et al. (2020). Associations with metabolites in Chinese suggest new metabolic roles in Alzheimer's and Parkinson's diseases. Human molecular genetics 29, 189-201. 10.1093/hmg/ddz246.
18. Xie, W., Wood, A.R., Lyssenko, V., Weedon, M.N., Knowles, J.W., Alkayyali, S., Assimes, T.L., Quertermous, T., Abbasi, F., Paananen, J., et al. (2013). Genetic variants associated with glycine metabolism and their role in insulin sensitivity and type 2 diabetes. Diabetes 62, 2141-2150. 10.2337/db12-0876.
19. Illig, T., Gieger, C., Zhai, G., Romisch-Margl, W., Wang-Sattler, R., Prehn, C., Altmaier, E., Kastenmuller, G., Kato, B.S., Mewes, H.W., et al. (2010). A genome-wide perspective of genetic variation in human metabolism. Nature genetics 42, 137-141. 10.1038/ng.507.
20. Hartiala, J.A., Tang, W.H., Wang, Z., Crow, A.L., Stewart, A.F., Roberts, R., McPherson, R., Erdmann, J., Willenborg, C., Hazen, S.L., and Allayee, H. (2016). Genome-wide association study and targeted metabolomics identifies sex-specific association of CPS1 with coronary artery disease. Nature communications 7, 10558. 10.1038/ncomms10558.
21. Imaizumi, A., Adachi, Y., Kawaguchi, T., Higasa, K., Tabara, Y., Sonomura, K., Sato, T.A., Takahashi, M., Mizukoshi, T., Yoshida, H.O., et al. (2019). Genetic basis for plasma amino acid concentrations based on absolute quantification: a genome-wide association study in the Japanese population. European journal of human genetics : EJHG 27, 621-630. 10.1038/s41431-018-0296-y.
22. Rhee, E.P., Ho, J.E., Chen, M.H., Shen, D., Cheng, S., Larson, M.G., Ghorbani, A., Shi, X., Helenius, I.T., O'Donnell, C.J., et al. (2013). A genome-wide association study of the human metabolome in a community-based cohort. Cell metabolism 18, 130-143. 10.1016/j.cmet.2013.06.013.
23. Draisma, H.H.M., Pool, R., Kobl, M., Jansen, R., Petersen, A.K., Vaarhorst, A.A.M., Yet, I., Haller, T., Demirkan, A., Esko, T., et al. (2015). Genome-wide association study identifies novel genetic variants contributing to variation in blood metabolite levels. Nature communications 6, 7208. 10.1038/ncomms8208.
24. Yu, B., de Vries, P.S., Metcalf, G.A., Wang, Z., Feofanova, E.V., Liu, X., Muzny, D.M., Wagenknecht, L.E., Gibbs, R.A., Morrison, A.C., and Boerwinkle, E. (2016). Whole genome sequence analysis of serum amino acid levels. Genome biology 17, 237. 10.1186/s13059-016-1106-x.
25. Jia, Q., Han, Y., Huang, P., Woodward, N.C., Gukasyan, J., Kettunen, J., Ala-Korpela, M., Anufrieva, O., Wang, Q., Perola, M., et al. (2019). Genetic Determinants of Circulating Glycine Levels and Risk of Coronary Artery Disease. J Am Heart Assoc 8, e011922. 10.1161/JAHA.119.011922.

26. Kettunen, J., Demirkan, A., Wurtz, P., Draisma, H.H., Haller, T., Rawal, R., Vaarhorst, A., Kangas, A.J., Lyytikainen, L.P., Pirinen, M., et al. (2016). Genome-wide study for circulating metabolites identifies 62 loci and reveals novel systemic effects of LPA. Nature communications 7, 11122. 10.1038/ncomms11122.
27. Yu, B., Zheng, Y., Alexander, D., Morrison, A.C., Coresh, J., and Boerwinkle, E. (2014). Genetic determinants influencing human serum metabolome among African Americans. PLoS Genet 10, e1004212. 10.1371/journal.pgen.1004212.
28. Wittemans, L.B.L., Lotta, L.A., Oliver-Williams, C., Stewart, I.D., Surendran, P., Karthikeyan, S., Day, F.R., Koulman, A., Imamura, F., Zeng, L., et al. (2019). Assessing the causal association of glycine with risk of cardio-metabolic diseases. Nature communications 10, 1060. 10.1038/s41467-019-08936-1.
29. Suhre, K., Shin, S.Y., Petersen, A.K., Mohney, R.P., Meredith, D., Wagele, B., Altmaier, E., CardioGram, Deloukas, P., Erdmann, J., et al. (2011). Human metabolic individuality in biomedical and pharmaceutical research. Nature 477, 54-60. 10.1038/nature10354.
30. Demirkan, A., Henneman, P., Verhoeven, A., Dharuri, H., Amin, N., van Klinken, J.B., Karssen, L.C., de Vries, B., Meissner, A., Goraler, S., et al. (2015). Insight in genome-wide association of metabolite quantitative traits by exome sequence analyses. PLoS Genet 11, e1004835. 10.1371/journal.pgen.1004835.
31. Kanai, M., Akiyama, M., Takahashi, A., Matoba, N., Momozawa, Y., Ikeda, M., Iwata, N., Ikegawa, S., Hirata, M., Matsuda, K., et al. (2018). Genetic analysis of quantitative traits in the Japanese population links cell types to complex human diseases. Nature genetics 50, 390-400. 10.1038/s41588-018-0047-6.
32. Gorski, M., van der Most, P.J., Teumer, A., Chu, A.Y., Li, M., Mijatovic, V., Nolte, I.M., Cocca, M., Taliun, D., Gomez, F., et al. (2017). 1000 Genomes-based meta-analysis identifies 10 novel loci for kidney function. Scientific reports 7, 45040. 10.1038/srep45040.
33. Pattaro, C., Teumer, A., Gorski, M., Chu, A.Y., Li, M., Mijatovic, V., Garnaas, M., Tin, A., Sorice, R., Li, Y., et al. (2016). Genetic associations at 53 loci highlight cell types and biological pathways relevant for kidney function. Nature communications 7, 10023. 10.1038/ncomms10023.
34. Graham, S.E., Nielsen, J.B., Zawistowski, M., Zhou, W., Fritsche, L.G., Gabrielsen, M.E., Skogholt, A.H., Surakka, I., Hornsby, W.E., Fermin, D., et al. (2019). Sex-specific and pleiotropic effects underlying kidney function identified from GWAS meta-analysis. Nature communications 10, 1847. 10.1038/s41467-019-09861-z.
35. Middelberg, R.P., Ferreira, M.A., Henders, A.K., Heath, A.C., Madden, P.A., Montgomery, G.W., Martin, N.G., and Whitfield, J.B. (2011). Genetic variants in LPL, OASL and TOMM40/APOE-C1-C2-C4 genes are associated with multiple cardiovascular-related traits. BMC Med Genet 12, 123. 10.1186/1471-2350-12-123.
36. Giri, A.K., Banerjee, P., Chakraborty, S., Kauser, Y., Undru, A., Roy, S., Parekatt, V., Ghosh, S., Tandon, N., and Bharadwaj, D. (2016). Genome wide association study of uric acid in Indian population and interaction of identified variants with Type 2 diabetes. Scientific reports 6, 21440. 10.1038/srep21440.
37. Nakatochi, M., Kanai, M., Nakayama, A., Hishida, A., Kawamura, Y., Ichihara, S., Akiyama, M., Ikezaki, H., Furusyo, N., Shimizu, S., et al. (2019). Genome-wide meta-analysis identifies multiple novel loci associated with serum uric acid levels in Japanese individuals. Communications biology 2, 115. 10.1038/s42003-019-0339-0.
38. Zemunik, T., Boban, M., Lauc, G., Jankovic, S., Rotim, K., Vatavuk, Z., Bencic, G., Dogas, Z., Boraska, V., Torlak, V., et al. (2009). Genome-wide association study of biochemical traits in Korcula Island, Croatia. Croat Med J 50, 23-33. 10.3325/cmj.2009.50.23.
39. Sulem, P., Gudbjartsson, D.F., Walters, G.B., Helgadottir, H.T., Helgason, A., Gudjonsson, S.A., Zanon,

C., Besenbacher, S., Bjornsdottir, G., Magnusson, O.T., et al. (2011). Identification of low-frequency variants associated with gout and serum uric acid levels. Nature genetics 43, 1127-1130. 10.1038/ng.972.
40. Cho, S.K., Kim, B., Myung, W., Chang, Y., Ryu, S., Kim, H.N., Kim, H.L., Kuo, P.H., Winkler, C.A., and Won, H.H. (2020). Polygenic analysis of the effect of common and low-frequency genetic variants on serum uric acid levels in Korean individuals. Scientific reports 10, 9179. 10.1038/s41598-020-66064-z.
41. Yasukochi, Y., Sakuma, J., Takeuchi, I., Kato, K., Oguri, M., Fujimaki, T., Horibe, H., and Yamada, Y. (2018). Identification of CDC42BPG as a novel susceptibility locus for hyperuricemia in a Japanese population. Mol Genet Genomics 293, 371-379. 10.1007/s00438-017-1394-1.
42. Yang, B., Mo, Z., Wu, C., Yang, H., Yang, X., He, Y., Gui, L., Zhou, L., Guo, H., Zhang, X., et al. (2014). A genome-wide association study identifies common variants influencing serum uric acid concentrations in a Chinese population. BMC Med Genomics 7, 10. 10.1186/1755-8794-7-10.
43. Karns, R., Zhang, G., Sun, G., Rao Indugula, S., Cheng, H., Havas-Augustin, D., Novokmet, N., Rudan, D., Durakovic, Z., Missoni, S., et al. (2012). Genome-wide association of serum uric acid concentration: replication of sequence variants in an island population of the Adriatic coast of Croatia. Annals of human genetics 76, 121-127. 10.1111/j.1469-1809.2011.00698.x.
44. Macias-Kauffer, L.R., Villamil-Ramirez, H., Leon-Mimila, P., Jacobo-Albavera, L., Posadas-Romero, C., Posadas-Sanchez, R., Lopez-Contreras, B.E., Moran-Ramos, S., Romero-Hidalgo, S., Acuna-Alonzo, V., et al. (2019). Genetic contributors to serum uric acid levels in Mexicans and their effect on premature coronary artery disease. Int J Cardiol 279, 168-173. 10.1016/j.ijcard.2018.09.107.
45. McArdle, P.F., Parsa, A., Chang, Y.P., Weir, M.R., O'Connell, J.R., Mitchell, B.D., and Shuldiner, A.R. (2008). Association of a common nonsynonymous variant in GLUT9 with serum uric acid levels in old order amish. Arthritis and rheumatism 58, 2874-2881. 10.1002/art.23752.
46. Lee, J., Lee, Y., Park, B., Won, S., Han, J.S., and Heo, N.J. (2018). Genome-wide association analysis identifies multiple loci associated with kidney disease-related traits in Korean populations. PloS one 13, e0194044. 10.1371/journal.pone.0194044.
47. Charles, B.A., Shriner, D., Doumatey, A., Chen, G., Zhou, J., Huang, H., Herbert, A., Gerry, N.P., Christman, M.F., Adeyemo, A., and Rotimi, C.N. (2011). A genome-wide association study of serum uric acid in African Americans. BMC Med Genomics 4, 17. 10.1186/1755-8794-4-17.
48. Zhang, D., Yang, M., Zhou, D., Li, Z., Cai, L., Bao, Y., Li, H., Shan, Z., Liu, J., Lv, D., et al. (2018). The polymorphism rs671 at ALDH2 associated with serum uric acid levels in Chinese Han males: A genome-wide association study. Gene 651, 62-69. 10.1016/j.gene.2018.01.064.
49. Rivera-Paredez, B., Macias-Kauffer, L., Fernandez-Lopez, J.C., Villalobos-Comparan, M., Martinez-Aguilar, M.M., de la Cruz-Montoya, A., Ramirez-Salazar, E.G., Villamil-Ramirez, H., Quiterio, M., Ramirez-Palacios, P., et al. (2019). Influence of Genetic and Non-Genetic Risk Factors for Serum Uric Acid Levels and Hyperuricemia in Mexicans. Nutrients 11. 10.3390/nu11061336.
50. Kolz, M., Johnson, T., Sanna, S., Teumer, A., Vitart, V., Perola, M., Mangino, M., Albrecht, E., Wallace, C., Farrall, M., et al. (2009). Meta-analysis of 28,141 individuals identifies common variants within five new loci that influence uric acid concentrations. PLoS Genet 5, e1000504. 10.1371/journal.pgen.1000504.
51. Li, W.D., Jiao, H., Wang, K., Zhang, C.K., Glessner, J.T., Grant, S.F., Zhao, H., Hakonarson, H., and Arlen Price, R. (2013). A genome wide association study of plasma uric acid levels in obese cases and never-overweight controls. Obesity (Silver Spring) 21, E490-494. 10.1002/oby.20303.
52. Voruganti, V.S., Kent, J.W., Jr., Debnath, S., Cole, S.A., Haack, K., Goring, H.H., Carless, M.A., Curran,

J.E., Johnson, M.P., Almasy, L., et al. (2013). Genome-wide association analysis confirms and extends the association of SLC2A9 with serum uric acid levels to Mexican Americans. Front Genet 4, 279. 10.3389/fgene.2013.00279.
53. Chen, G., Shriner, D., Doumatey, A.P., Zhou, J., Bentley, A.R., Lei, L., Adeyemo, A., and Rotimi, C.N. (2020). Refining genome-wide associated loci for serum uric acid in individuals with African ancestry. Human molecular genetics 29, 506-514. 10.1093/hmg/ddz272.
54. Son, C.N., Bang, S.Y., Kim, S.H., Sung, Y.K., Bae, S.C., and Jun, J.B. (2017). ABCG2 Polymorphism Is Associated with Hyperuricemia in a Study of a Community-Based Korean Cohort. J Korean Med Sci 32, 1451-1459. 10.3346/jkms.2017.32.9.1451.
55. Kettunen, J., Tukiainen, T., Sarin, A.P., Ortega-Alonso, A., Tikkanen, E., Lyytikainen, L.P., Kangas, A.J., Soininen, P., Wurtz, P., Silander, K., et al. (2012). Genome-wide association study identifies multiple loci influencing human serum metabolite levels. Nature genetics 44, 269-276. 10.1038/ng.1073.
56. Lotta, L.A., Scott, R.A., Sharp, S.J., Burgess, S., Luan, J., Tillin, T., Schmidt, A.F., Imamura, F., Stewart, I.D., Perry, J.R., et al. (2016). Genetic Predisposition to an Impaired Metabolism of the Branched-Chain Amino Acids and Risk of Type 2 Diabetes: A Mendelian Randomisation Analysis. PLoS Med 13, e1002179. 10.1371/journal.pmed.1002179.
57. Al-Khelaifi, F., Diboun, I., Donati, F., Botre, F., Abraham, D., Hingorani, A., Albagha, O., Georgakopoulos, C., Suhre, K., Yousri, N.A., and Elrayess, M.A. (2019). Metabolic GWAS of elite athletes reveals novel genetically-influenced metabolites associated with athletic performance. Scientific reports 9, 19889. 10.1038/s41598-019-56496-7.
58. Yousri, N.A., Fakhro, K.A., Robay, A., Rodriguez-Flores, J.L., Mohney, R.P., Zeriri, H., Odeh, T., Kader, S.A., Aldous, E.K., Thareja, G., et al. (2018). Whole-exome sequencing identifies common and rare variant metabolic QTLs in a Middle Eastern population. Nature communications 9, 333. 10.1038/s41467-017-01972-9.
59. Suhre, K., Wallaschofski, H., Raffler, J., Friedrich, N., Haring, R., Michael, K., Wasner, C., Krebs, A., Kronenberg, F., Chang, D., et al. (2011). A genome-wide association study of metabolic traits in human urine. Nature genetics 43, 565-569. 10.1038/ng.837.
60. Raffler, J., Friedrich, N., Arnold, M., Kacprowski, T., Rueedi, R., Altmaier, E., Bergmann, S., Budde, K., Gieger, C., Homuth, G., et al. (2015). Genome-Wide Association Study with Targeted and Non-targeted NMR Metabolomics Identifies 15 Novel Loci of Urinary Human Metabolic Individuality. PLoS Genet 11, e1005487. 10.1371/journal.pgen.1005487.
61. Nicholson, G., Rantalainen, M., Li, J.V., Maher, A.D., Malmodin, D., Ahmadi, K.R., Faber, J.H., Barrett, A., Min, J.L., Rayner, N.W., et al. (2011). A genome-wide metabolic QTL analysis in Europeans implicates two loci shaped by recent positive selection. PLoS Genet 7, e1002270. 10.1371/journal.pgen.1002270.
62. Luo, S., Feofanova, E.V., Tin, A., Tung, S., Rhee, E.P., Coresh, J., Arking, D.E., Surapaneni, A., Schlosser, P., Li, Y., et al. (2021). Genome-wide association study of serum metabolites in the African American Study of Kidney Disease and Hypertension. Kidney Int 100, 430-439. 10.1016/j.kint.2021.03.026.
63. Fall, T., Salihovic, S., Brandmaier, S., Nowak, C., Ganna, A., Gustafsson, S., Broeckling, C.D., Prenni, J.E., Kastenmuller, G., Peters, A., et al. (2016). Non-targeted metabolomics combined with genetic analyses identifies bile acid synthesis and phospholipid metabolism as being associated with incident type 2 diabetes. Diabetologia 59, 2114-2124. 10.1007/s00125-016-4041-1.
64. Geidenstam, N., Hsu, Y.H., Astley, C.M., Mercader, J.M., Ridderstrale, M., Gonzalez, M.E., Gonzalez, C.,

Hirschhorn, J.N., and Salem, R.M. (2019). Using metabolite profiling to construct and validate a metabolite risk score for predicting future weight gain. PloS one 14, e0222445. 10.1371/journal.pone.0222445.
65. Rueedi, R., Ledda, M., Nicholls, A.W., Salek, R.M., Marques-Vidal, P., Morya, E., Sameshima, K., Montoliu, I., Da Silva, L., Collino, S., et al. (2014). Genome-wide association study of metabolic traits reveals novel gene-metabolite-disease links. PLoS Genet 10, e1004132. 10.1371/journal.pgen.1004132.
66. Wang, Z., Zhu, Q., Liu, Y., Chen, S., Zhang, Y., Ma, Q., Chen, X., Liu, C., Lei, H., Chen, H., et al. (2021). Genome-wide association study of metabolites in patients with coronary artery disease identified novel metabolite quantitative trait loci. Clin Transl Med 11, e290. 10.1002/ctm2.290.
67. Li, M., Maruthur, N.M., Loomis, S.J., Pietzner, M., North, K.E., Mei, H., Morrison, A.C., Friedrich, N., Pankow, J.S., Nauck, M., et al. (2017). Genome-wide association study of 1,5-anhydroglucitol identifies novel genetic loci linked to glucose metabolism. Scientific reports 7, 2812. 10.1038/s41598-017-02287-x.
68. Rhee, E.P., Yang, Q., Yu, B., Liu, X., Cheng, S., Deik, A., Pierce, K.A., Bullock, K., Ho, J.E., Levy, D., et al. (2016). An exome array study of the plasma metabolome. Nature communications 7, 12360. 10.1038/ncomms12360.

References for Supplementary Table 6.

[S1] Sakaue, S., Kanai, M., Tanigawa, Y., Karjalainen, J., Kurki, M., Koshiba, S., Narita, A., Konuma, T., Yamamoto, K., Akiyama, M., et al. (2021). A cross-population atlas of genetic associations for 220 human phenotypes. Nature genetics 53, 1415-1424. 10.1038/s41588-021-00931-x.

[S2] Shin, S.Y., Fauman, E.B., Petersen, A.K., Krumsiek, J., Santos, R., Huang, J., Arnold, M., Erte, I., Forgetta, V., Yang, T.P., et al. (2014). An atlas of genetic influences on human blood metabolites. Nature genetics 46, 543-550. 10.1038/ng.2982.

[S3] Yin, X., Chan, L.S., Bose, D., Jackson, A.U., VandeHaar, P., Locke, A.E., Fuchsberger, C., Stringham, H.M., Welch, R., Yu, K., et al. (2022). Genome-wide association studies of metabolites in Finnish men identify disease-relevant loci. Nature communications 13, 1644. 10.1038/s41467-022-29143-5.

[S4] Rhee, E.P., Ho, J.E., Chen, M.H., Shen, D., Cheng, S., Larson, M.G., Ghorbani, A., Shi, X., Helenius, I.T., O'Donnell, C.J., et al. (2013). A genome-wide association study of the human metabolome in a community-based cohort. Cell metabolism 18, 130-143. 10.1016/j.cmet.2013.06.013.

[S5] Draisma, H.H.M., Pool, R., Kobl, M., Jansen, R., Petersen, A.K., Vaarhorst, A.A.M., Yet, I., Haller, T., Demirkan, A., Esko, T., et al. (2015). Genome-wide association study identifies novel genetic variants contributing to variation in blood metabolite levels. Nature communications 6, 7208. 10.1038/ncomms8208.

[S6] Yu, B., de Vries, P.S., Metcalf, G.A., Wang, Z., Feofanova, E.V., Liu, X., Muzny, D.M., Wagenknecht, L.E., Gibbs, R.A., Morrison, A.C., and Boerwinkle, E. (2016). Whole genome sequence analysis of serum amino acid levels. Genome biology 17, 237. 10.1186/s13059-016-1106-x.

[S7] Long, T., Hicks, M., Yu, H.C., Biggs, W.H., Kirkness, E.F., Menni, C., Zierer, J., Small, K.S., Mangino, M., Messier, H., et al. (2017). Whole-genome sequencing identifies common-to-rare variants associated with human blood metabolites. Nature genetics 49, 568-578. 10.1038/ng.3809.

[S8] Suhre, K., Shin, S.Y., Petersen, A.K., Mohney, R.P., Meredith, D., Wagele, B., Altmaier, E., CardioGram, Deloukas, P., Erdmann, J., et al. (2011). Human metabolic individuality in biomedical and pharmaceutical research. Nature 477, 54-60. 10.1038/nature10354.

[S9] Feofanova, E.V., Chen, H., Dai, Y., Jia, P., Grove, M.L., Morrison, A.C., Qi, Q., Daviglus, M., Cai, J., North, K.E., et al. (2020). A Genome-wide Association Study Discovers 46 Loci of the Human Metabolome in the Hispanic Community Health Study/Study of Latinos. American journal of human genetics 107, 849-863. 10.1016/j.ajhg.2020.09.003.

[S10] Lotta, L.A., Pietzner, M., Stewart, I.D., Wittemans, L.B.L., Li, C., Bonelli, R., Raffler, J., Biggs, E.K., Oliver-Williams, C., Auyeung, V.P.W., et al. (2021). A cross-platform approach identifies genetic regulators of human metabolism and health. Nature genetics 53, 54-64. 10.1038/s41588-020-00751-5.

[S11] Kettunen, J., Demirkan, A., Wurtz, P., Draisma, H.H., Haller, T., Rawal, R., Vaarhorst, A., Kangas, A.J., Lyytikainen, L.P., Pirinen, M., et al. (2016). Genome-wide study for circulating metabolites identifies 62 loci and reveals novel systemic effects of LPA. Nature communications 7, 11122. 10.1038/ncomms11122.

[S12] Tukiainen, T., Kettunen, J., Kangas, A.J., Lyytikainen, L.P., Soininen, P., Sarin, A.P., Tikkanen, E., O'Reilly, P.F., Savolainen, M.J., Kaski, K., et al. (2012). Detailed metabolic and genetic characterization reveals new associations for 30 known lipid loci. Human molecular genetics 21, 1444-1455. 10.1093/hmg/ddr581.

[S13] Kettunen, J., Tukiainen, T., Sarin, A.P., Ortega-Alonso, A., Tikkanen, E., Lyytikainen, L.P., Kangas, A.J., Soininen, P., Wurtz, P., Silander, K., et al. (2012). Genome-wide association study identifies multiple loci

influencing human serum metabolite levels. Nature genetics 44, 269-276. 10.1038/ng.1073.
[S14] Feofanova, E.V., Chen, H., Dai, Y., Jia, P., Grove, M.L., Morrison, A.C., Qi, Q., Daviglus, M., Cai, J., North, K.E., et al. (2020). A Genome-wide Association Study Discovers 46 Loci of the Human Metabolome in the Hispanic Community Health Study/Study of Latinos. American journal of human genetics 107, 849-863. 10.1016/j.ajhg.2020.09.003.
[S15] Wessel, J., Chu, A.Y., Willems, S.M., Wang, S., Yaghootkar, H., Brody, J.A., Dauriz, M., Hivert, M.F., Raghavan, S., Lipovich, L., et al. (2015). Low-frequency and rare exome chip variants associate with fasting glucose and type 2 diabetes susceptibility. Nature communications 6, 5897. 10.1038/ncomms6897.
[S16] Sabatti, C., Service, S.K., Hartikainen, A.L., Pouta, A., Ripatti, S., Brodsky, J., Jones, C.G., Zaitlen, N.A., Varilo, T., Kaakinen, M., et al. (2009). Genome-wide association analysis of metabolic traits in a birth cohort from a founder population. Nature genetics 41, 35-46. 10.1038/ng.271.
[S17] Chen, J., Spracklen, C.N., Marenne, G., Varshney, A., Corbin, L.J., Luan, J., Willems, S.M., Wu, Y., Zhang, X., Horikoshi, M., et al. (2021). The trans-ancestral genomic architecture of glycemic traits. Nature genetics 53, 840-860. 10.1038/s41588-021-00852-9.
[S18] Hayes, M.G., Urbanek, M., Hivert, M.F., Armstrong, L.L., Morrison, J., Guo, C., Lowe, L.P., Scheftner, D.A., Pluzhnikov, A., Levine, D.M., et al. (2013). Identification of HKDC1 and BACE2 as genes influencing glycemic traits during pregnancy through genome-wide association studies. Diabetes 62, 3282-3291. 10.2337/db12-1692.
[S19] Kim, Y.J., Go, M.J., Hu, C., Hong, C.B., Kim, Y.K., Lee, J.Y., Hwang, J.Y., Oh, J.H., Kim, D.J., Kim, N.H., et al. (2011). Large-scale genome-wide association studies in East Asians identify new genetic loci influencing metabolic traits. Nature genetics 43, 990-995. 10.1038/ng.939.
[S20] Manning, A.K., Hivert, M.F., Scott, R.A., Grimsby, J.L., Bouatia-Naji, N., Chen, H., Rybin, D., Liu, C.T., Bielak, L.F., Prokopenko, I., et al. (2012). A genome-wide approach accounting for body mass index identifies genetic variants influencing fasting glycemic traits and insulin resistance. Nature genetics 44, 659-669. 10.1038/ng.2274.
[S21] Hwang, J.Y., Sim, X., Wu, Y., Liang, J., Tabara, Y., Hu, C., Hara, K., Tam, C.H., Cai, Q., Zhao, Q., et al. (2015). Genome-wide association meta-analysis identifies novel variants associated with fasting plasma glucose in East Asians. Diabetes 64, 291-298. 10.2337/db14-0563.
[S22] Kanai, M., Akiyama, M., Takahashi, A., Matoba, N., Momozawa, Y., Ikeda, M., Iwata, N., Ikegawa, S., Hirata, M., Matsuda, K., et al. (2018). Genetic analysis of quantitative traits in the Japanese population links cell types to complex human diseases. Nature genetics 50, 390-400. 10.1038/s41588-018-0047-6.
[S23] Bouatia-Naji, N., Rocheleau, G., Van Lommel, L., Lemaire, K., Schuit, F., Cavalcanti-Proenca, C., Marchand, M., Hartikainen, A.L., Sovio, U., De Graeve, F., et al. (2008). A polymorphism within the G6PC2 gene is associated with fasting plasma glucose levels. Science (New York, N.Y.) 320, 1085-1088. 10.1126/science.1156849.
[S24] Dupuis, J., Langenberg, C., Prokopenko, I., Saxena, R., Soranzo, N., Jackson, A.U., Wheeler, E., Glazer, N.L., Bouatia-Naji, N., Gloyn, A.L., et al. (2010). New genetic loci implicated in fasting glucose homeostasis and their impact on type 2 diabetes risk. Nature genetics 42, 105-116. 10.1038/ng.520.
[S25] Nagy, R., Boutin, T.S., Marten, J., Huffman, J.E., Kerr, S.M., Campbell, A., Evenden, L., Gibson, J., Amador, C., Howard, D.M., et al. (2017). Exploration of haplotype research consortium imputation for genome-wide association studies in 20,032 Generation Scotland participants. Genome Med 9, 23. 10.1186/s13073-017-0414-4.

[S26] Sinnott-Armstrong, N., Tanigawa, Y., Amar, D., Mars, N., Benner, C., Aguirre, M., Venkataraman, G.R., Wainberg, M., Ollila, H.M., Kiiskinen, T., et al. (2021). Genetics of 35 blood and urine biomarkers in the UK Biobank. Nature genetics 53, 185-194. 10.1038/s41588-020-00757-z.
[S27] Chung, R.H., Chiu, Y.F., Wang, W.C., Hwu, C.M., Hung, Y.J., Lee, I.T., Chuang, L.M., Quertermous, T., Rotter, J.I., Chen, Y.I., et al. (2021). Multi-omics analysis identifies CpGs near G6PC2 mediating the effects of genetic variants on fasting glucose. Diabetologia 64, 1613-1625. 10.1007/s00125-021-05449-9.
[S28] Chen, W.M., Erdos, M.R., Jackson, A.U., Saxena, R., Sanna, S., Silver, K.D., Timpson, N.J., Hansen, T., Orru, M., Grazia Piras, M., et al. (2008). Variations in the G6PC2/ABCB11 genomic region are associated with fasting glucose levels. The Journal of clinical investigation 118, 2620-2628. 10.1172/JCI34566.
[S29] Wojcik, G.L., Graff, M., Nishimura, K.K., Tao, R., Haessler, J., Gignoux, C.R., Highland, H.M., Patel, Y.M., Sorokin, E.P., Avery, C.L., et al. (2019). Genetic analyses of diverse populations improves discovery for complex traits. Nature 570, 514-518. 10.1038/s41586-019-1310-4.
[S30] Mahajan, A., Sim, X., Ng, H.J., Manning, A., Rivas, M.A., Highland, H.M., Locke, A.E., Grarup, N., Im, H.K., Cingolani, P., et al. (2015). Identification and functional characterization of G6PC2 coding variants influencing glycemic traits define an effector transcript at the G6PC2-ABCB11 locus. PLoS Genet 11, e1004876. 10.1371/journal.pgen.1004876.
[S31] Prokopenko, I., Langenberg, C., Florez, J.C., Saxena, R., Soranzo, N., Thorleifsson, G., Loos, R.J., Manning, A.K., Jackson, A.U., Aulchenko, Y., et al. (2009). Variants in MTNR1B influence fasting glucose levels. Nature genetics 41, 77-81. 10.1038/ng.290.
[S32] Lagou, V., Magi, R., Hottenga, J.J., Grallert, H., Perry, J.R.B., Bouatia-Naji, N., Marullo, L., Rybin, D., Jansen, R., Min, J.L., et al. (2021). Sex-dimorphic genetic effects and novel loci for fasting glucose and insulin variability. Nature communications 12, 24. 10.1038/s41467-020-19366-9.
[S33] Masotti, M., Guo, B., and Wu, B. (2019). Pleiotropy informed adaptive association test of multiple traits using genome-wide association study summary data. Biometrics 75, 1076-1085. 10.1111/biom.13076.
[S34] Rasmussen-Torvik, L.J., Guo, X., Bowden, D.W., Bertoni, A.G., Sale, M.M., Yao, J., Bluemke, D.A., Goodarzi, M.O., Chen, Y.I., Vaidya, D., et al. (2012). Fasting glucose GWAS candidate region analysis across ethnic groups in the Multiethnic Study of Atherosclerosis (MESA). Genetic epidemiology 36, 384-391. 10.1002/gepi.21632.
[S35] Spracklen, C.N., Shi, J., Vadlamudi, S., Wu, Y., Zou, M., Raulerson, C.K., Davis, J.P., Zeynalzadeh, M., Jackson, K., Yuan, W., et al. (2018). Identification and functional analysis of glycemic trait loci in the China Health and Nutrition Survey. PLoS Genet 14, e1007275. 10.1371/journal.pgen.1007275.
[S36] Li, Y., Sekula, P., Wuttke, M., Wahrheit, J., Hausknecht, B., Schultheiss, U.T., Gronwald, W., Schlosser, P., Tucci, S., Ekici, A.B., et al. (2018). Genome-Wide Association Studies of Metabolites in Patients with CKD Identify Multiple Loci and Illuminate Tubular Transport Mechanisms. J Am Soc Nephrol 29, 1513-1524. 10.1681/ASN.2017101099.
[S37] Bar, N., Korem, T., Weissbrod, O., Zeevi, D., Rothschild, D., Leviatan, S., Kosower, N., Lotan-Pompan, M., Weinberger, A., Le Roy, C.I., et al. (2020). A reference map of potential determinants for the human serum metabolome. Nature 588, 135-140. 10.1038/s41586-020-2896-2.
[S38] Teslovich, T.M., Kim, D.S., Yin, X., Stancakova, A., Jackson, A.U., Wielscher, M., Naj, A., Perry, J.R.B., Huyghe, J.R., Stringham, H.M., et al. (2018). Identification of seven novel loci associated with amino acid levels using single-variant and gene-based tests in 8545 Finnish men from the METSIM study. Human molecular genetics 27, 1664-1674. 10.1093/hmg/ddy067.

[S39] Krumsiek, J., Suhre, K., Evans, A.M., Mitchell, M.W., Mohney, R.P., Milburn, M.V., Wagele, B., Romisch-Margl, W., Illig, T., Adamski, J., et al. (2012). Mining the unknown: a systems approach to metabolite identification combining genetic and metabolic information. PLoS Genet 8, e1003005. 10.1371/journal.pgen.1003005.
[S40] Chai, J.F., Raichur, S., Khor, I.W., Torta, F., Chew, W.S., Herr, D.R., Ching, J., Kovalik, J.P., Khoo, C.M., Wenk, M.R., et al. (2020). Associations with metabolites in Chinese suggest new metabolic roles in Alzheimer's and Parkinson's diseases. Human molecular genetics 29, 189-201. 10.1093/hmg/ddz246.
[S41] Xie, W., Wood, A.R., Lyssenko, V., Weedon, M.N., Knowles, J.W., Alkayyali, S., Assimes, T.L., Quertermous, T., Abbasi, F., Paananen, J., et al. (2013). Genetic variants associated with glycine metabolism and their role in insulin sensitivity and type 2 diabetes. Diabetes 62, 2141-2150. 10.2337/db12-0876.
[S42] Illig, T., Gieger, C., Zhai, G., Romisch-Margl, W., Wang-Sattler, R., Prehn, C., Altmaier, E., Kastenmuller, G., Kato, B.S., Mewes, H.W., et al. (2010). A genome-wide perspective of genetic variation in human metabolism. Nature genetics 42, 137-141. 10.1038/ng.507.
[S43] Koshiba, S., Motoike, I.N., Saigusa, D., Inoue, J., Aoki, Y., Tadaka, S., Shirota, M., Katsuoka, F., Tamiya, G., Minegishi, N., et al. (2020). Identification of critical genetic variants associated with metabolic phenotypes of the Japanese population. Communications biology 3, 662. 10.1038/s42003-020-01383-5.
[S44] Hartiala, J.A., Tang, W.H., Wang, Z., Crow, A.L., Stewart, A.F., Roberts, R., McPherson, R., Erdmann, J., Willenborg, C., Hazen, S.L., and Allayee, H. (2016). Genome-wide association study and targeted metabolomics identifies sex-specific association of CPS1 with coronary artery disease. Nature communications 7, 10558. 10.1038/ncomms10558.
[S45] Imaizumi, A., Adachi, Y., Kawaguchi, T., Higasa, K., Tabara, Y., Sonomura, K., Sato, T.A., Takahashi, M., Mizukoshi, T., Yoshida, H.O., et al. (2019). Genetic basis for plasma amino acid concentrations based on absolute quantification: a genome-wide association study in the Japanese population. European journal of human genetics : EJHG 27, 621-630. 10.1038/s41431-018-0296-y.
[S46] Jia, Q., Han, Y., Huang, P., Woodward, N.C., Gukasyan, J., Kettunen, J., Ala-Korpela, M., Anufrieva, O., Wang, Q., Perola, M., et al. (2019). Genetic Determinants of Circulating Glycine Levels and Risk of Coronary Artery Disease. J Am Heart Assoc 8, e011922. 10.1161/JAHA.119.011922.
[S47] Yu, B., Zheng, Y., Alexander, D., Morrison, A.C., Coresh, J., and Boerwinkle, E. (2014). Genetic determinants influencing human serum metabolome among African Americans. PLoS Genet 10, e1004212. 10.1371/journal.pgen.1004212.
[S48] Wittemans, L.B.L., Lotta, L.A., Oliver-Williams, C., Stewart, I.D., Surendran, P., Karthikeyan, S., Day, F.R., Koulman, A., Imamura, F., Zeng, L., et al. (2019). Assessing the causal association of glycine with risk of cardio-metabolic diseases. Nature communications 10, 1060. 10.1038/s41467-019-08936-1.
[S49] Demirkan, A., Henneman, P., Verhoeven, A., Dharuri, H., Amin, N., van Klinken, J.B., Karssen, L.C., de Vries, B., Meissner, A., Goraler, S., et al. (2015). Insight in genome-wide association of metabolite quantitative traits by exome sequence analyses. PLoS Genet 11, e1004835. 10.1371/journal.pgen.1004835.
[S50] Yet, I., Menni, C., Shin, S.Y., Mangino, M., Soranzo, N., Adamski, J., Suhre, K., Spector, T.D., Kastenmuller, G., and Bell, J.T. (2016). Genetic Influences on Metabolite Levels: A Comparison across Metabolomic Platforms. PloS one 11, e0153672. 10.1371/journal.pone.0153672.
[S51] Gorski, M., van der Most, P.J., Teumer, A., Chu, A.Y., Li, M., Mijatovic, V., Nolte, I.M., Cocca, M., Taliun, D., Gomez, F., et al. (2017). 1000 Genomes-based meta-analysis identifies 10 novel loci for kidney function. Scientific reports 7, 45040. 10.1038/srep45040.

[S52] Pattaro, C., Teumer, A., Gorski, M., Chu, A.Y., Li, M., Mijatovic, V., Garnaas, M., Tin, A., Sorice, R., Li, Y., et al. (2016). Genetic associations at 53 loci highlight cell types and biological pathways relevant for kidney function. Nature communications 7, 10023. 10.1038/ncomms10023.
[S53] Graham, S.E., Nielsen, J.B., Zawistowski, M., Zhou, W., Fritsche, L.G., Gabrielsen, M.E., Skogholt, A.H., Surakka, I., Hornsby, W.E., Fermin, D., et al. (2019). Sex-specific and pleiotropic effects underlying kidney function identified from GWAS meta-analysis. Nature communications 10, 1847. 10.1038/s41467-019-09861-z.
[S54] Middelberg, R.P., Ferreira, M.A., Henders, A.K., Heath, A.C., Madden, P.A., Montgomery, G.W., Martin, N.G., and Whitfield, J.B. (2011). Genetic variants in LPL, OASL and TOMM40/APOE-C1-C2-C4 genes are associated with multiple cardiovascular-related traits. BMC Med Genet 12, 123. 10.1186/1471-2350-12-123.
[S55] Giri, A.K., Banerjee, P., Chakraborty, S., Kauser, Y., Undru, A., Roy, S., Parekatt, V., Ghosh, S., Tandon, N., and Bharadwaj, D. (2016). Genome wide association study of uric acid in Indian population and interaction of identified variants with Type 2 diabetes. Scientific reports 6, 21440. 10.1038/srep21440.
[S56] Nakatochi, M., Kanai, M., Nakayama, A., Hishida, A., Kawamura, Y., Ichihara, S., Akiyama, M., Ikezaki, H., Furusyo, N., Shimizu, S., et al. (2019). Genome-wide meta-analysis identifies multiple novel loci associated with serum uric acid levels in Japanese individuals. Communications biology 2, 115. 10.1038/s42003-019-0339-0.
[S57] Zemunik, T., Boban, M., Lauc, G., Jankovic, S., Rotim, K., Vatavuk, Z., Bencic, G., Dogas, Z., Boraska, V., Torlak, V., et al. (2009). Genome-wide association study of biochemical traits in Korcula Island, Croatia. Croat Med J 50, 23-33. 10.3325/cmj.2009.50.23.
[S58] Sulem, P., Gudbjartsson, D.F., Walters, G.B., Helgadottir, H.T., Helgason, A., Gudjonsson, S.A., Zanon, C., Besenbacher, S., Bjornsdottir, G., Magnusson, O.T., et al. (2011). Identification of low-frequency variants associated with gout and serum uric acid levels. Nature genetics 43, 1127-1130. 10.1038/ng.972.
[S59] Cho, S.K., Kim, B., Myung, W., Chang, Y., Ryu, S., Kim, H.N., Kim, H.L., Kuo, P.H., Winkler, C.A., and Won, H.H. (2020). Polygenic analysis of the effect of common and low-frequency genetic variants on serum uric acid levels in Korean individuals. Scientific reports 10, 9179. 10.1038/s41598-020-66064-z.
[S60] Yasukochi, Y., Sakuma, J., Takeuchi, I., Kato, K., Oguri, M., Fujimaki, T., Horibe, H., and Yamada, Y. (2018). Identification of CDC42BPG as a novel susceptibility locus for hyperuricemia in a Japanese population. Mol Genet Genomics 293, 371-379. 10.1007/s00438-017-1394-1.
[S61] Yang, B., Mo, Z., Wu, C., Yang, H., Yang, X., He, Y., Gui, L., Zhou, L., Guo, H., Zhang, X., et al. (2014). A genome-wide association study identifies common variants influencing serum uric acid concentrations in a Chinese population. BMC Med Genomics 7, 10. 10.1186/1755-8794-7-10.
[S62] Karns, R., Zhang, G., Sun, G., Rao Indugula, S., Cheng, H., Havas-Augustin, D., Novokmet, N., Rudan, D., Durakovic, Z., Missoni, S., et al. (2012). Genome-wide association of serum uric acid concentration: replication of sequence variants in an island population of the Adriatic coast of Croatia. Annals of human genetics 76, 121-127. 10.1111/j.1469-1809.2011.00698.x.
[S63] Macias-Kauffer, L.R., Villamil-Ramirez, H., Leon-Mimila, P., Jacobo-Albavera, L., Posadas-Romero, C., Posadas-Sanchez, R., Lopez-Contreras, B.E., Moran-Ramos, S., Romero-Hidalgo, S., Acuna-Alonzo, V., et al. (2019). Genetic contributors to serum uric acid levels in Mexicans and their effect on premature coronary artery disease. Int J Cardiol 279, 168-173. 10.1016/j.ijcard.2018.09.107.
[S64] McArdle, P.F., Parsa, A., Chang, Y.P., Weir, M.R., O'Connell, J.R., Mitchell, B.D., and Shuldiner, A.R. (2008). Association of a common nonsynonymous variant in GLUT9 with serum uric acid levels in old order

amish. Arthritis and rheumatism 58, 2874-2881. 10.1002/art.23752.
[S65] Lee, J., Lee, Y., Park, B., Won, S., Han, J.S., and Heo, N.J. (2018). Genome-wide association analysis identifies multiple loci associated with kidney disease-related traits in Korean populations. PloS one 13, e0194044. 10.1371/journal.pone.0194044.
[S66] Charles, B.A., Shriner, D., Doumatey, A., Chen, G., Zhou, J., Huang, H., Herbert, A., Gerry, N.P., Christman, M.F., Adeyemo, A., and Rotimi, C.N. (2011). A genome-wide association study of serum uric acid in African Americans. BMC Med Genomics 4, 17. 10.1186/1755-8794-4-17.
[S67] Zhang, D., Yang, M., Zhou, D., Li, Z., Cai, L., Bao, Y., Li, H., Shan, Z., Liu, J., Lv, D., et al. (2018). The polymorphism rs671 at ALDH2 associated with serum uric acid levels in Chinese Han males: A genome-wide association study. Gene 651, 62-69. 10.1016/j.gene.2018.01.064.
[S68] Rivera-Paredez, B., Macias-Kauffer, L., Fernandez-Lopez, J.C., Villalobos-Comparan, M., Martinez-Aguilar, M.M., de la Cruz-Montoya, A., Ramirez-Salazar, E.G., Villamil-Ramirez, H., Quiterio, M., Ramirez-Palacios, P., et al. (2019). Influence of Genetic and Non-Genetic Risk Factors for Serum Uric Acid Levels and Hyperuricemia in Mexicans. Nutrients 11. 10.3390/nu11061336.
[S69] Kolz, M., Johnson, T., Sanna, S., Teumer, A., Vitart, V., Perola, M., Mangino, M., Albrecht, E., Wallace, C., Farrall, M., et al. (2009). Meta-analysis of 28,141 individuals identifies common variants within five new loci that influence uric acid concentrations. PLoS Genet 5, e1000504. 10.1371/journal.pgen.1000504.
[S70] Li, W.D., Jiao, H., Wang, K., Zhang, C.K., Glessner, J.T., Grant, S.F., Zhao, H., Hakonarson, H., and Arlen Price, R. (2013). A genome wide association study of plasma uric acid levels in obese cases and never-overweight controls. Obesity (Silver Spring) 21, E490-494. 10.1002/oby.20303.
[S71] Voruganti, V.S., Kent, J.W., Jr., Debnath, S., Cole, S.A., Haack, K., Goring, H.H., Carless, M.A., Curran, J.E., Johnson, M.P., Almasy, L., et al. (2013). Genome-wide association analysis confirms and extends the association of SLC2A9 with serum uric acid levels to Mexican Americans. Front Genet 4, 279. 10.3389/fgene.2013.00279.
[S72] Chen, G., Shriner, D., Doumatey, A.P., Zhou, J., Bentley, A.R., Lei, L., Adeyemo, A., and Rotimi, C.N. (2020). Refining genome-wide associated loci for serum uric acid in individuals with African ancestry. Human molecular genetics 29, 506-514. 10.1093/hmg/ddz272.
[S73] Son, C.N., Bang, S.Y., Kim, S.H., Sung, Y.K., Bae, S.C., and Jun, J.B. (2017). ABCG2 Polymorphism Is Associated with Hyperuricemia in a Study of a Community-Based Korean Cohort. J Korean Med Sci 32, 1451-1459. 10.3346/jkms.2017.32.9.1451.
[S74] Lotta, L.A., Scott, R.A., Sharp, S.J., Burgess, S., Luan, J., Tillin, T., Schmidt, A.F., Imamura, F., Stewart, I.D., Perry, J.R., et al. (2016). Genetic Predisposition to an Impaired Metabolism of the Branched-Chain Amino Acids and Risk of Type 2 Diabetes: A Mendelian Randomisation Analysis. PLoS Med 13, e1002179. 10.1371/journal.pmed.1002179.
[S75] Fall, T., Salihovic, S., Brandmaier, S., Nowak, C., Ganna, A., Gustafsson, S., Broeckling, C.D., Prenni, J.E., Kastenmuller, G., Peters, A., et al. (2016). Non-targeted metabolomics combined with genetic analyses identifies bile acid synthesis and phospholipid metabolism as being associated with incident type 2 diabetes. Diabetologia 59, 2114-2124. 10.1007/s00125-016-4041-1.
[S76] Geidenstam, N., Hsu, Y.H., Astley, C.M., Mercader, J.M., Ridderstrale, M., Gonzalez, M.E., Gonzalez, C., Hirschhorn, J.N., and Salem, R.M. (2019). Using metabolite profiling to construct and validate a metabolite risk score for predicting future weight gain. PloS one 14, e0222445. 10.1371/journal.pone.0222445.
[S77] Yu, B., Li, A.H., Muzny, D., Veeraraghavan, N., de Vries, P.S., Bis, J.C., Musani, S.K., Alexander, D.,

Morrison, A.C., Franco, O.H., et al. (2015). Association of Rare Loss-Of-Function Alleles in HAL, Serum Histidine: Levels and Incident Coronary Heart Disease. Circ Cardiovasc Genet 8, 351-355. 10.1161/CIRCGENETICS.114.000697.
[S78] Rhee, E.P., Yang, Q., Yu, B., Liu, X., Cheng, S., Deik, A., Pierce, K.A., Bullock, K., Ho, J.E., Levy, D., et al. (2016). An exome array study of the plasma metabolome. Nature communications 7, 12360. 10.1038/ncomms12360.

References for Supplementary Table 7.

[S1] Sakaue, S., Kanai, M., Tanigawa, Y., Karjalainen, J., Kurki, M., Koshiba, S., Narita, A., Konuma, T., Yamamoto, K., Akiyama, M., et al. (2021). A cross-population atlas of genetic associations for 220 human phenotypes. Nature genetics 53, 1415-1424. 10.1038/s41588-021-00931-x.

[S2] Shin, S.Y., Fauman, E.B., Petersen, A.K., Krumsiek, J., Santos, R., Huang, J., Arnold, M., Erte, I., Forgetta, V., Yang, T.P., et al. (2014). An atlas of genetic influences on human blood metabolites. Nature genetics 46, 543-550. 10.1038/ng.2982.

[S3] Yin, X., Chan, L.S., Bose, D., Jackson, A.U., VandeHaar, P., Locke, A.E., Fuchsberger, C., Stringham, H.M., Welch, R., Yu, K., et al. (2022). Genome-wide association studies of metabolites in Finnish men identify disease-relevant loci. Nature communications 13, 1644. 10.1038/s41467-022-29143-5.

[S4] Long, T., Hicks, M., Yu, H.C., Biggs, W.H., Kirkness, E.F., Menni, C., Zierer, J., Small, K.S., Mangino, M., Messier, H., et al. (2017). Whole-genome sequencing identifies common-to-rare variants associated with human blood metabolites. Nature genetics 49, 568-578. 10.1038/ng.3809.

[S5] Yu, B., de Vries, P.S., Metcalf, G.A., Wang, Z., Feofanova, E.V., Liu, X., Muzny, D.M., Wagenknecht, L.E., Gibbs, R.A., Morrison, A.C., and Boerwinkle, E. (2016). Whole genome sequence analysis of serum amino acid levels. Genome biology 17, 237. 10.1186/s13059-016-1106-x.

[S6] Feofanova, E.V., Chen, H., Dai, Y., Jia, P., Grove, M.L., Morrison, A.C., Qi, Q., Daviglus, M., Cai, J., North, K.E., et al. (2020). A Genome-wide Association Study Discovers 46 Loci of the Human Metabolome in the Hispanic Community Health Study/Study of Latinos. American journal of human genetics 107, 849-863. 10.1016/j.ajhg.2020.09.003.

[S7] Luo, S., Feofanova, E.V., Tin, A., Tung, S., Rhee, E.P., Coresh, J., Arking, D.E., Surapaneni, A., Schlosser, P., Li, Y., et al. (2021). Genome-wide association study of serum metabolites in the African American Study of Kidney Disease and Hypertension. Kidney Int 100, 430-439. 10.1016/j.kint.2021.03.026.

[S8] Rhee, E.P., Ho, J.E., Chen, M.H., Shen, D., Cheng, S., Larson, M.G., Ghorbani, A., Shi, X., Helenius, I.T., O'Donnell, C.J., et al. (2013). A genome-wide association study of the human metabolome in a community-based cohort. Cell metabolism 18, 130-143. 10.1016/j.cmet.2013.06.013.

[S9] Draisma, H.H.M., Pool, R., Kobl, M., Jansen, R., Petersen, A.K., Vaarhorst, A.A.M., Yet, I., Haller, T., Demirkan, A., Esko, T., et al. (2015). Genome-wide association study identifies novel genetic variants contributing to variation in blood metabolite levels. Nature communications 6, 7208. 10.1038/ncomms8208.

[S10] Suhre, K., Shin, S.Y., Petersen, A.K., Mohney, R.P., Meredith, D., Wagele, B., Altmaier, E., CardioGram, Deloukas, P., Erdmann, J., et al. (2011). Human metabolic individuality in biomedical and pharmaceutical research. Nature 477, 54-60. 10.1038/nature10354.

[S11] Lotta, L.A., Pietzner, M., Stewart, I.D., Wittemans, L.B.L., Li, C., Bonelli, R., Raffler, J., Biggs, E.K., Oliver-Williams, C., Auyeung, V.P.W., et al. (2021). A cross-platform approach identifies genetic regulators of human metabolism and health. Nature genetics 53, 54-64. 10.1038/s41588-020-00751-5.

[S12] Korostishevsky, M., Steves, C.J., Malkin, I., Spector, T., Williams, F.M., and Livshits, G. (2016). Genomics and metabolomics of muscular mass in a community-based sample of UK females. European journal of human genetics : EJHG 24, 277-283. 10.1038/ejhg.2015.85.

[S13] Krumsiek, J., Suhre, K., Evans, A.M., Mitchell, M.W., Mohney, R.P., Milburn, M.V., Wagele, B., Romisch-Margl, W., Illig, T., Adamski, J., et al. (2012). Mining the unknown: a systems approach to metabolite identification combining genetic and metabolic information. PLoS Genet 8, e1003005.

10.1371/journal.pgen.1003005.
[S14] Yet, I., Menni, C., Shin, S.Y., Mangino, M., Soranzo, N., Adamski, J., Suhre, K., Spector, T.D., Kastenmuller, G., and Bell, J.T. (2016). Genetic Influences on Metabolite Levels: A Comparison across Metabolomic Platforms. PloS one 11, e0153672. 10.1371/journal.pone.0153672.
[S15] Kettunen, J., Demirkan, A., Wurtz, P., Draisma, H.H., Haller, T., Rawal, R., Vaarhorst, A., Kangas, A.J., Lyytikainen, L.P., Pirinen, M., et al. (2016). Genome-wide study for circulating metabolites identifies 62 loci and reveals novel systemic effects of LPA. Nature communications 7, 11122. 10.1038/ncomms11122.
[S16] Tukiainen, T., Kettunen, J., Kangas, A.J., Lyytikainen, L.P., Soininen, P., Sarin, A.P., Tikkanen, E., O'Reilly, P.F., Savolainen, M.J., Kaski, K., et al. (2012). Detailed metabolic and genetic characterization reveals new associations for 30 known lipid loci. Human molecular genetics 21, 1444-1455. 10.1093/hmg/ddr581.
[S17] Kettunen, J., Tukiainen, T., Sarin, A.P., Ortega-Alonso, A., Tikkanen, E., Lyytikainen, L.P., Kangas, A.J., Soininen, P., Wurtz, P., Silander, K., et al. (2012). Genome-wide association study identifies multiple loci influencing human serum metabolite levels. Nature genetics 44, 269-276. 10.1038/ng.1073.
[S18] Feofanova, E.V., Chen, H., Dai, Y., Jia, P., Grove, M.L., Morrison, A.C., Qi, Q., Daviglus, M., Cai, J., North, K.E., et al. (2020). A Genome-wide Association Study Discovers 46 Loci of the Human Metabolome in the Hispanic Community Health Study/Study of Latinos. American journal of human genetics 107, 849-863. 10.1016/j.ajhg.2020.09.003.
[S19] Li, M., Maruthur, N.M., Loomis, S.J., Pietzner, M., North, K.E., Mei, H., Morrison, A.C., Friedrich, N., Pankow, J.S., Nauck, M., et al. (2017). Genome-wide association study of 1,5-anhydroglucitol identifies novel genetic loci linked to glucose metabolism. Scientific reports 7, 2812. 10.1038/s41598-017-02287-x.
[S20] Wessel, J., Chu, A.Y., Willems, S.M., Wang, S., Yaghootkar, H., Brody, J.A., Dauriz, M., Hivert, M.F., Raghavan, S., Lipovich, L., et al. (2015). Low-frequency and rare exome chip variants associate with fasting glucose and type 2 diabetes susceptibility. Nature communications 6, 5897. 10.1038/ncomms6897.
[S21] Sabatti, C., Service, S.K., Hartikainen, A.L., Pouta, A., Ripatti, S., Brodsky, J., Jones, C.G., Zaitlen, N.A., Varilo, T., Kaakinen, M., et al. (2009). Genome-wide association analysis of metabolic traits in a birth cohort from a founder population. Nature genetics 41, 35-46. 10.1038/ng.271.
[S22] Chen, J., Spracklen, C.N., Marenne, G., Varshney, A., Corbin, L.J., Luan, J., Willems, S.M., Wu, Y., Zhang, X., Horikoshi, M., et al. (2021). The trans-ancestral genomic architecture of glycemic traits. Nature genetics 53, 840-860. 10.1038/s41588-021-00852-9.
[S23] Hayes, M.G., Urbanek, M., Hivert, M.F., Armstrong, L.L., Morrison, J., Guo, C., Lowe, L.P., Scheftner, D.A., Pluzhnikov, A., Levine, D.M., et al. (2013). Identification of HKDC1 and BACE2 as genes influencing glycemic traits during pregnancy through genome-wide association studies. Diabetes 62, 3282-3291. 10.2337/db12-1692.
[S24] Kim, Y.J., Go, M.J., Hu, C., Hong, C.B., Kim, Y.K., Lee, J.Y., Hwang, J.Y., Oh, J.H., Kim, D.J., Kim, N.H., et al. (2011). Large-scale genome-wide association studies in East Asians identify new genetic loci influencing metabolic traits. Nature genetics 43, 990-995. 10.1038/ng.939.
[S25] Manning, A.K., Hivert, M.F., Scott, R.A., Grimsby, J.L., Bouatia-Naji, N., Chen, H., Rybin, D., Liu, C.T., Bielak, L.F., Prokopenko, I., et al. (2012). A genome-wide approach accounting for body mass index identifies genetic variants influencing fasting glycemic traits and insulin resistance. Nature genetics 44, 659-669. 10.1038/ng.2274.
[S26] Hwang, J.Y., Sim, X., Wu, Y., Liang, J., Tabara, Y., Hu, C., Hara, K., Tam, C.H., Cai, Q., Zhao, Q., et al.

(2015). Genome-wide association meta-analysis identifies novel variants associated with fasting plasma glucose in East Asians. Diabetes 64, 291-298. 10.2337/db14-0563.
[S27] Kanai, M., Akiyama, M., Takahashi, A., Matoba, N., Momozawa, Y., Ikeda, M., Iwata, N., Ikegawa, S., Hirata, M., Matsuda, K., et al. (2018). Genetic analysis of quantitative traits in the Japanese population links cell types to complex human diseases. Nature genetics 50, 390-400. 10.1038/s41588-018-0047-6.
[S28] Bouatia-Naji, N., Rocheleau, G., Van Lommel, L., Lemaire, K., Schuit, F., Cavalcanti-Proenca, C., Marchand, M., Hartikainen, A.L., Sovio, U., De Graeve, F., et al. (2008). A polymorphism within the G6PC2 gene is associated with fasting plasma glucose levels. Science (New York, N.Y.) 320, 1085-1088. 10.1126/science.1156849.
[S29] Dupuis, J., Langenberg, C., Prokopenko, I., Saxena, R., Soranzo, N., Jackson, A.U., Wheeler, E., Glazer, N.L., Bouatia-Naji, N., Gloyn, A.L., et al. (2010). New genetic loci implicated in fasting glucose homeostasis and their impact on type 2 diabetes risk. Nature genetics 42, 105-116. 10.1038/ng.520.
[S30] Nagy, R., Boutin, T.S., Marten, J., Huffman, J.E., Kerr, S.M., Campbell, A., Evenden, L., Gibson, J., Amador, C., Howard, D.M., et al. (2017). Exploration of haplotype research consortium imputation for genome-wide association studies in 20,032 Generation Scotland participants. Genome Med 9, 23. 10.1186/s13073-017-0414-4.
[S31] Sinnott-Armstrong, N., Tanigawa, Y., Amar, D., Mars, N., Benner, C., Aguirre, M., Venkataraman, G.R., Wainberg, M., Ollila, H.M., Kiiskinen, T., et al. (2021). Genetics of 35 blood and urine biomarkers in the UK Biobank. Nature genetics 53, 185-194. 10.1038/s41588-020-00757-z.
[S32] Chung, R.H., Chiu, Y.F., Wang, W.C., Hwu, C.M., Hung, Y.J., Lee, I.T., Chuang, L.M., Quertermous, T., Rotter, J.I., Chen, Y.I., et al. (2021). Multi-omics analysis identifies CpGs near G6PC2 mediating the effects of genetic variants on fasting glucose. Diabetologia 64, 1613-1625. 10.1007/s00125-021-05449-9.
[S33] Chen, W.M., Erdos, M.R., Jackson, A.U., Saxena, R., Sanna, S., Silver, K.D., Timpson, N.J., Hansen, T., Orru, M., Grazia Piras, M., et al. (2008). Variations in the G6PC2/ABCB11 genomic region are associated with fasting glucose levels. The Journal of clinical investigation 118, 2620-2628. 10.1172/JCI34566.
[S34] Wojcik, G.L., Graff, M., Nishimura, K.K., Tao, R., Haessler, J., Gignoux, C.R., Highland, H.M., Patel, Y.M., Sorokin, E.P., Avery, C.L., et al. (2019). Genetic analyses of diverse populations improves discovery for complex traits. Nature 570, 514-518. 10.1038/s41586-019-1310-4.
[S35] Mahajan, A., Sim, X., Ng, H.J., Manning, A., Rivas, M.A., Highland, H.M., Locke, A.E., Grarup, N., Im, H.K., Cingolani, P., et al. (2015). Identification and functional characterization of G6PC2 coding variants influencing glycemic traits define an effector transcript at the G6PC2-ABCB11 locus. PLoS Genet 11, e1004876. 10.1371/journal.pgen.1004876.
[S36] Prokopenko, I., Langenberg, C., Florez, J.C., Saxena, R., Soranzo, N., Thorleifsson, G., Loos, R.J., Manning, A.K., Jackson, A.U., Aulchenko, Y., et al. (2009). Variants in MTNR1B influence fasting glucose levels. Nature genetics 41, 77-81. 10.1038/ng.290.
[S37] Lagou, V., Magi, R., Hottenga, J.J., Grallert, H., Perry, J.R.B., Bouatia-Naji, N., Marullo, L., Rybin, D., Jansen, R., Min, J.L., et al. (2021). Sex-dimorphic genetic effects and novel loci for fasting glucose and insulin variability. Nature communications 12, 24. 10.1038/s41467-020-19366-9.
[S38] Masotti, M., Guo, B., and Wu, B. (2019). Pleiotropy informed adaptive association test of multiple traits using genome-wide association study summary data. Biometrics 75, 1076-1085. 10.1111/biom.13076.
[S39] Rasmussen-Torvik, L.J., Guo, X., Bowden, D.W., Bertoni, A.G., Sale, M.M., Yao, J., Bluemke, D.A., Goodarzi, M.O., Chen, Y.I., Vaidya, D., et al. (2012). Fasting glucose GWAS candidate region analysis across

ethnic groups in the Multiethnic Study of Atherosclerosis (MESA). Genetic epidemiology 36, 384-391. 10.1002/gepi.21632.
[S40] Spracklen, C.N., Shi, J., Vadlamudi, S., Wu, Y., Zou, M., Raulerson, C.K., Davis, J.P., Zeynalzadeh, M., Jackson, K., Yuan, W., et al. (2018). Identification and functional analysis of glycemic trait loci in the China Health and Nutrition Survey. PLoS Genet 14, e1007275. 10.1371/journal.pgen.1007275.
[S41] Gorski, M., van der Most, P.J., Teumer, A., Chu, A.Y., Li, M., Mijatovic, V., Nolte, I.M., Cocca, M., Taliun, D., Gomez, F., et al. (2017). 1000 Genomes-based meta-analysis identifies 10 novel loci for kidney function. Scientific reports 7, 45040. 10.1038/srep45040.
[S42] Pattaro, C., Teumer, A., Gorski, M., Chu, A.Y., Li, M., Mijatovic, V., Garnaas, M., Tin, A., Sorice, R., Li, Y., et al. (2016). Genetic associations at 53 loci highlight cell types and biological pathways relevant for kidney function. Nature communications 7, 10023. 10.1038/ncomms10023.
[S43] Graham, S.E., Nielsen, J.B., Zawistowski, M., Zhou, W., Fritsche, L.G., Gabrielsen, M.E., Skogholt, A.H., Surakka, I., Hornsby, W.E., Fermin, D., et al. (2019). Sex-specific and pleiotropic effects underlying kidney function identified from GWAS meta-analysis. Nature communications 10, 1847. 10.1038/s41467-019-09861-z.
[S44] Li, Y., Sekula, P., Wuttke, M., Wahrheit, J., Hausknecht, B., Schultheiss, U.T., Gronwald, W., Schlosser, P., Tucci, S., Ekici, A.B., et al. (2018). Genome-Wide Association Studies of Metabolites in Patients with CKD Identify Multiple Loci and Illuminate Tubular Transport Mechanisms. J Am Soc Nephrol 29, 1513-1524. 10.1681/ASN.2017101099.
[S45] Bar, N., Korem, T., Weissbrod, O., Zeevi, D., Rothschild, D., Leviatan, S., Kosower, N., Lotan-Pompan, M., Weinberger, A., Le Roy, C.I., et al. (2020). A reference map of potential determinants for the human serum metabolome. Nature 588, 135-140. 10.1038/s41586-020-2896-2.
[S46] Teslovich, T.M., Kim, D.S., Yin, X., Stancakova, A., Jackson, A.U., Wielscher, M., Naj, A., Perry, J.R.B., Huyghe, J.R., Stringham, H.M., et al. (2018). Identification of seven novel loci associated with amino acid levels using single-variant and gene-based tests in 8545 Finnish men from the METSIM study. Human molecular genetics 27, 1664-1674. 10.1093/hmg/ddy067.
[S47] Chai, J.F., Raichur, S., Khor, I.W., Torta, F., Chew, W.S., Herr, D.R., Ching, J., Kovalik, J.P., Khoo, C.M., Wenk, M.R., et al. (2020). Associations with metabolites in Chinese suggest new metabolic roles in Alzheimer's and Parkinson's diseases. Human molecular genetics 29, 189-201. 10.1093/hmg/ddz246.
[S48] Xie, W., Wood, A.R., Lyssenko, V., Weedon, M.N., Knowles, J.W., Alkayyali, S., Assimes, T.L., Quertermous, T., Abbasi, F., Paananen, J., et al. (2013). Genetic variants associated with glycine metabolism and their role in insulin sensitivity and type 2 diabetes. Diabetes 62, 2141-2150. 10.2337/db12-0876.
[S49] Illig, T., Gieger, C., Zhai, G., Romisch-Margl, W., Wang-Sattler, R., Prehn, C., Altmaier, E., Kastenmuller, G., Kato, B.S., Mewes, H.W., et al. (2010). A genome-wide perspective of genetic variation in human metabolism. Nature genetics 42, 137-141. 10.1038/ng.507.
[S50] Koshiba, S., Motoike, I.N., Saigusa, D., Inoue, J., Aoki, Y., Tadaka, S., Shirota, M., Katsuoka, F., Tamiya, G., Minegishi, N., et al. (2020). Identification of critical genetic variants associated with metabolic phenotypes of the Japanese population. Communications biology 3, 662. 10.1038/s42003-020-01383-5.
[S51] Hartiala, J.A., Tang, W.H., Wang, Z., Crow, A.L., Stewart, A.F., Roberts, R., McPherson, R., Erdmann, J., Willenborg, C., Hazen, S.L., and Allayee, H. (2016). Genome-wide association study and targeted metabolomics identifies sex-specific association of CPS1 with coronary artery disease. Nature communications 7, 10558. 10.1038/ncomms10558.

[S52] Imaizumi, A., Adachi, Y., Kawaguchi, T., Higasa, K., Tabara, Y., Sonomura, K., Sato, T.A., Takahashi, M., Mizukoshi, T., Yoshida, H.O., et al. (2019). Genetic basis for plasma amino acid concentrations based on absolute quantification: a genome-wide association study in the Japanese population. European journal of human genetics : EJHG 27, 621-630. 10.1038/s41431-018-0296-y.
[S53] Jia, Q., Han, Y., Huang, P., Woodward, N.C., Gukasyan, J., Kettunen, J., Ala-Korpela, M., Anufrieva, O., Wang, Q., Perola, M., et al. (2019). Genetic Determinants of Circulating Glycine Levels and Risk of Coronary Artery Disease. J Am Heart Assoc 8, e011922. 10.1161/JAHA.119.011922.
[S54] Yu, B., Zheng, Y., Alexander, D., Morrison, A.C., Coresh, J., and Boerwinkle, E. (2014). Genetic determinants influencing human serum metabolome among African Americans. PLoS Genet 10, e1004212. 10.1371/journal.pgen.1004212.
[S55] Wittemans, L.B.L., Lotta, L.A., Oliver-Williams, C., Stewart, I.D., Surendran, P., Karthikeyan, S., Day, F.R., Koulman, A., Imamura, F., Zeng, L., et al. (2019). Assessing the causal association of glycine with risk of cardio-metabolic diseases. Nature communications 10, 1060. 10.1038/s41467-019-08936-1.
[S56] Demirkan, A., Henneman, P., Verhoeven, A., Dharuri, H., Amin, N., van Klinken, J.B., Karssen, L.C., de Vries, B., Meissner, A., Goraler, S., et al. (2015). Insight in genome-wide association of metabolite quantitative traits by exome sequence analyses. PLoS Genet 11, e1004835. 10.1371/journal.pgen.1004835.
[S57] Middelberg, R.P., Ferreira, M.A., Henders, A.K., Heath, A.C., Madden, P.A., Montgomery, G.W., Martin, N.G., and Whitfield, J.B. (2011). Genetic variants in LPL, OASL and TOMM40/APOE-C1-C2-C4 genes are associated with multiple cardiovascular-related traits. BMC Med Genet 12, 123. 10.1186/1471-2350-12-123.
[S58] Giri, A.K., Banerjee, P., Chakraborty, S., Kauser, Y., Undru, A., Roy, S., Parekatt, V., Ghosh, S., Tandon, N., and Bharadwaj, D. (2016). Genome wide association study of uric acid in Indian population and interaction of identified variants with Type 2 diabetes. Scientific reports 6, 21440. 10.1038/srep21440.
[S59] Nakatochi, M., Kanai, M., Nakayama, A., Hishida, A., Kawamura, Y., Ichihara, S., Akiyama, M., Ikezaki, H., Furusyo, N., Shimizu, S., et al. (2019). Genome-wide meta-analysis identifies multiple novel loci associated with serum uric acid levels in Japanese individuals. Communications biology 2, 115. 10.1038/s42003-019-0339-0.
[S60] Zemunik, T., Boban, M., Lauc, G., Jankovic, S., Rotim, K., Vatavuk, Z., Bencic, G., Dogas, Z., Boraska, V., Torlak, V., et al. (2009). Genome-wide association study of biochemical traits in Korcula Island, Croatia. Croat Med J 50, 23-33. 10.3325/cmj.2009.50.23.
[S61] Sulem, P., Gudbjartsson, D.F., Walters, G.B., Helgadottir, H.T., Helgason, A., Gudjonsson, S.A., Zanon, C., Besenbacher, S., Bjornsdottir, G., Magnusson, O.T., et al. (2011). Identification of low-frequency variants associated with gout and serum uric acid levels. Nature genetics 43, 1127-1130. 10.1038/ng.972.
[S62] Cho, S.K., Kim, B., Myung, W., Chang, Y., Ryu, S., Kim, H.N., Kim, H.L., Kuo, P.H., Winkler, C.A., and Won, H.H. (2020). Polygenic analysis of the effect of common and low-frequency genetic variants on serum uric acid levels in Korean individuals. Scientific reports 10, 9179. 10.1038/s41598-020-66064-z.
[S63] Yasukochi, Y., Sakuma, J., Takeuchi, I., Kato, K., Oguri, M., Fujimaki, T., Horibe, H., and Yamada, Y. (2018). Identification of CDC42BPG as a novel susceptibility locus for hyperuricemia in a Japanese population. Mol Genet Genomics 293, 371-379. 10.1007/s00438-017-1394-1.
[S64] Yang, B., Mo, Z., Wu, C., Yang, H., Yang, X., He, Y., Gui, L., Zhou, L., Guo, H., Zhang, X., et al. (2014). A genome-wide association study identifies common variants influencing serum uric acid concentrations in a Chinese population. BMC Med Genomics 7, 10. 10.1186/1755-8794-7-10.
[S65] Karns, R., Zhang, G., Sun, G., Rao Indugula, S., Cheng, H., Havas-Augustin, D., Novokmet, N., Rudan,

D., Durakovic, Z., Missoni, S., et al. (2012). Genome-wide association of serum uric acid concentration: replication of sequence variants in an island population of the Adriatic coast of Croatia. Annals of human genetics 76, 121-127. 10.1111/j.1469-1809.2011.00698.x.
[S66] Macias-Kauffer, L.R., Villamil-Ramirez, H., Leon-Mimila, P., Jacobo-Albavera, L., Posadas-Romero, C., Posadas-Sanchez, R., Lopez-Contreras, B.E., Moran-Ramos, S., Romero-Hidalgo, S., Acuna-Alonzo, V., et al. (2019). Genetic contributors to serum uric acid levels in Mexicans and their effect on premature coronary artery disease. Int J Cardiol 279, 168-173. 10.1016/j.ijcard.2018.09.107.
[S67] McArdle, P.F., Parsa, A., Chang, Y.P., Weir, M.R., O'Connell, J.R., Mitchell, B.D., and Shuldiner, A.R. (2008). Association of a common nonsynonymous variant in GLUT9 with serum uric acid levels in old order amish. Arthritis and rheumatism 58, 2874-2881. 10.1002/art.23752.
[S68] Lee, J., Lee, Y., Park, B., Won, S., Han, J.S., and Heo, N.J. (2018). Genome-wide association analysis identifies multiple loci associated with kidney disease-related traits in Korean populations. PloS one 13, e0194044. 10.1371/journal.pone.0194044.
[S69] Charles, B.A., Shriner, D., Doumatey, A., Chen, G., Zhou, J., Huang, H., Herbert, A., Gerry, N.P., Christman, M.F., Adeyemo, A., and Rotimi, C.N. (2011). A genome-wide association study of serum uric acid in African Americans. BMC Med Genomics 4, 17. 10.1186/1755-8794-4-17.
[S70] Zhang, D., Yang, M., Zhou, D., Li, Z., Cai, L., Bao, Y., Li, H., Shan, Z., Liu, J., Lv, D., et al. (2018). The polymorphism rs671 at ALDH2 associated with serum uric acid levels in Chinese Han males: A genome-wide association study. Gene 651, 62-69. 10.1016/j.gene.2018.01.064.
[S71] Rivera-Paredez, B., Macias-Kauffer, L., Fernandez-Lopez, J.C., Villalobos-Comparan, M., Martinez-Aguilar, M.M., de la Cruz-Montoya, A., Ramirez-Salazar, E.G., Villamil-Ramirez, H., Quiterio, M., Ramirez-Palacios, P., et al. (2019). Influence of Genetic and Non-Genetic Risk Factors for Serum Uric Acid Levels and Hyperuricemia in Mexicans. Nutrients 11. 10.3390/nu11061336.
[S72] Kolz, M., Johnson, T., Sanna, S., Teumer, A., Vitart, V., Perola, M., Mangino, M., Albrecht, E., Wallace, C., Farrall, M., et al. (2009). Meta-analysis of 28,141 individuals identifies common variants within five new loci that influence uric acid concentrations. PLoS Genet 5, e1000504. 10.1371/journal.pgen.1000504.
[S73] Li, W.D., Jiao, H., Wang, K., Zhang, C.K., Glessner, J.T., Grant, S.F., Zhao, H., Hakonarson, H., and Arlen Price, R. (2013). A genome wide association study of plasma uric acid levels in obese cases and never-overweight controls. Obesity (Silver Spring) 21, E490-494. 10.1002/oby.20303.
[S74] Voruganti, V.S., Kent, J.W., Jr., Debnath, S., Cole, S.A., Haack, K., Goring, H.H., Carless, M.A., Curran, J.E., Johnson, M.P., Almasy, L., et al. (2013). Genome-wide association analysis confirms and extends the association of SLC2A9 with serum uric acid levels to Mexican Americans. Front Genet 4, 279. 10.3389/fgene.2013.00279.
[S75] Chen, G., Shriner, D., Doumatey, A.P., Zhou, J., Bentley, A.R., Lei, L., Adeyemo, A., and Rotimi, C.N. (2020). Refining genome-wide associated loci for serum uric acid in individuals with African ancestry. Human molecular genetics 29, 506-514. 10.1093/hmg/ddz272.
[S76] Son, C.N., Bang, S.Y., Kim, S.H., Sung, Y.K., Bae, S.C., and Jun, J.B. (2017). ABCG2 Polymorphism Is Associated with Hyperuricemia in a Study of a Community-Based Korean Cohort. J Korean Med Sci 32, 1451-1459. 10.3346/jkms.2017.32.9.1451.
[S77] Lotta, L.A., Scott, R.A., Sharp, S.J., Burgess, S., Luan, J., Tillin, T., Schmidt, A.F., Imamura, F., Stewart, I.D., Perry, J.R., et al. (2016). Genetic Predisposition to an Impaired Metabolism of the Branched-Chain Amino Acids and Risk of Type 2 Diabetes: A Mendelian Randomisation Analysis. PLoS Med 13, e1002179.

10.1371/journal.pmed.1002179.
[S78] Fall, T., Salihovic, S., Brandmaier, S., Nowak, C., Ganna, A., Gustafsson, S., Broeckling, C.D., Prenni, J.E., Kastenmuller, G., Peters, A., et al. (2016). Non-targeted metabolomics combined with genetic analyses identifies bile acid synthesis and phospholipid metabolism as being associated with incident type 2 diabetes. Diabetologia 59, 2114-2124. 10.1007/s00125-016-4041-1.
[S79] Geidenstam, N., Hsu, Y.H., Astley, C.M., Mercader, J.M., Ridderstrale, M., Gonzalez, M.E., Gonzalez, C., Hirschhorn, J.N., and Salem, R.M. (2019). Using metabolite profiling to construct and validate a metabolite risk score for predicting future weight gain. PloS one 14, e0222445. 10.1371/journal.pone.0222445.
[S80] Lind, L. (2020). Genetic Determinants of Clustering of Cardiometabolic Risk Factors in U.K. Biobank. Metabolic syndrome and related disorders 18, 121-127. 10.1089/met.2019.0096.
[S81] Li, D., Kang, H., Lee, S., and Won, S. (2020). Progressive effects of single-nucleotide polymorphisms on 16 phenotypic traits based on longitudinal data. Genes & genomics 42, 393-403. 10.1007/s13258-019-00902-x.
[S82] Kalsbeek, A., Veenstra, J., Westra, J., Disselkoen, C., Koch, K., McKenzie, K.A., O'Bott, J., Vander Woude, J., Fischer, K., Shearer, G.C., et al. (2018). A genome-wide association study of red-blood cell fatty acids and ratios incorporating dietary covariates: Framingham Heart Study Offspring Cohort. PloS one 13, e0194882. 10.1371/journal.pone.0194882.
[S83] Wu, J.H., Lemaitre, R.N., Manichaikul, A., Guan, W., Tanaka, T., Foy, M., Kabagambe, E.K., Djousse, L., Siscovick, D., Fretts, A.M., et al. (2013). Genome-wide association study identifies novel loci associated with concentrations of four plasma phospholipid fatty acids in the de novo lipogenesis pathway: results from the Cohorts for Heart and Aging Research in Genomic Epidemiology (CHARGE) consortium. Circ Cardiovasc Genet 6, 171-183. 10.1161/CIRCGENETICS.112.964619.
[S84] Feofanova, E.V., Yu, B., Metcalf, G.A., Liu, X., Muzny, D., Below, J.E., Wagenknecht, L.E., Gibbs, R.A., Morrison, A.C., and Boerwinkle, E. (2018). Sequence-Based Analysis of Lipid-Related Metabolites in a Multiethnic Study. Genetics 209, 607-616. 10.1534/genetics.118.300751.
[S85] Yu, B., Li, A.H., Muzny, D., Veeraraghavan, N., de Vries, P.S., Bis, J.C., Musani, S.K., Alexander, D., Morrison, A.C., Franco, O.H., et al. (2015). Association of Rare Loss-Of-Function Alleles in HAL, Serum Histidine: Levels and Incident Coronary Heart Disease. Circ Cardiovasc Genet 8, 351-355. 10.1161/CIRCGENETICS.114.000697.
[S86] Raffler, J., Friedrich, N., Arnold, M., Kacprowski, T., Rueedi, R., Altmaier, E., Bergmann, S., Budde, K., Gieger, C., Homuth, G., et al. (2015). Genome-Wide Association Study with Targeted and Non-targeted NMR Metabolomics Identifies 15 Novel Loci of Urinary Human Metabolic Individuality. PLoS Genet 11, e1005487. 10.1371/journal.pgen.1005487.
[S87] Rueedi, R., Ledda, M., Nicholls, A.W., Salek, R.M., Marques-Vidal, P., Morya, E., Sameshima, K., Montoliu, I., Da Silva, L., Collino, S., et al. (2014). Genome-wide association study of metabolic traits reveals novel gene-metabolite-disease links. PLoS Genet 10, e1004132. 10.1371/journal.pgen.1004132.
[S88] Suhre, K., Wallaschofski, H., Raffler, J., Friedrich, N., Haring, R., Michael, K., Wasner, C., Krebs, A., Kronenberg, F., Chang, D., et al. (2011). A genome-wide association study of metabolic traits in human urine. Nature genetics 43, 565-569. 10.1038/ng.837.
[S89] Wang, Z., Zhu, Q., Liu, Y., Chen, S., Zhang, Y., Ma, Q., Chen, X., Liu, C., Lei, H., Chen, H., et al. (2021). Genome-wide association study of metabolites in patients with coronary artery disease identified novel metabolite quantitative trait loci. Clin Transl Med 11, e290. 10.1002/ctm2.290.

[S90] Schlosser, P., Li, Y., Sekula, P., Raffler, J., Grundner-Culemann, F., Pietzner, M., Cheng, Y., Wuttke, M., Steinbrenner, I., Schultheiss, U.T., et al. (2020). Genetic studies of urinary metabolites illuminate mechanisms of detoxification and excretion in humans. Nature genetics 52, 167-176. 10.1038/s41588-019-0567-8.
[S91] Al-Khelaifi, F., Diboun, I., Donati, F., Botre, F., Abraham, D., Hingorani, A., Albagha, O., Georgakopoulos, C., Suhre, K., Yousri, N.A., and Elrayess, M.A. (2019). Metabolic GWAS of elite athletes reveals novel genetically-influenced metabolites associated with athletic performance. Scientific reports 9, 19889. 10.1038/s41598-019-56496-7.
[S92] Rhee, E.P., Yang, Q., Yu, B., Liu, X., Cheng, S., Deik, A., Pierce, K.A., Bullock, K., Ho, J.E., Levy, D., et al. (2016). An exome array study of the plasma metabolome. Nature communications 7, 12360. 10.1038/ncomms12360.
